# The hibernation-derived compound SUL-138 shifts the mitochondrial proteome towards fatty acid metabolism and prevents cognitive decline and amyloid plaque formation in an Alzheimer’s disease mouse model

**DOI:** 10.1186/s13195-022-01127-z

**Published:** 2022-12-09

**Authors:** Christina F. de Veij Mestdagh, Frank Koopmans, Jonathan C. Breiter, Jaap A. Timmerman, Pieter C. Vogelaar, Guido Krenning, Huibert D. Mansvelder, August B. Smit, Robert H. Henning, Ronald E. van Kesteren

**Affiliations:** 1 Department of Molecular and Cellular Neurobiology, Center for Neurogenomics and Cognitive Research, Amsterdam Neuroscience, Vrije Universiteit Amsterdam, Amsterdam, the Netherlands; 2 Department of Clinical Pharmacy and Pharmacology, University Medical Center Groningen, Groningen, the Netherlands; 3 Alzheimer Center Amsterdam, Vrije Universiteit Amsterdam and Amsterdam UMC location VUmc , Amsterdam, The Netherlands; 4 Department of Integrative Neurophysiology, Center for Neurogenomics and Cognitive Research, Amsterdam Neuroscience, Vrije Universiteit Amsterdam, Amsterdam, the Netherlands; 5 Sulfateq B.V., Groningen, The Netherlands; 6 Department of Pathology and Medical Biology, University Medical Center Groningen, Groningen, the Netherlands

**Keywords:** Alzheimer’s disease, APP/PS1 mice, Hibernation-derived compound, SUL-138, Chromanols, Long-term potentiation, Hippocampus-dependent contextual fear memory, Amyloid-beta plaques, Mass-spectrometry, Mitochondria, Fatty acid metabolism

## Abstract

**Background:**

Alzheimer’s disease (AD) is the most prevalent neurodegenerative disease worldwide and remains without effective cure. Increasing evidence is supporting the mitochondrial cascade hypothesis, proposing that loss of mitochondrial fitness and subsequent ROS and ATP imbalance are important contributors to AD pathophysiology.

**Methods:**

Here, we tested the effects of SUL-138, a small hibernation-derived molecule that supports mitochondrial bioenergetics via complex I/IV activation, on molecular, physiological, behavioral, and pathological outcomes in APP/PS1 and wildtype mice.

**Results:**

SUL-138 treatment rescued long-term potentiation and hippocampal memory impairments and decreased beta-amyloid plaque load in APP/PS1 mice. This was paralleled by a partial rescue of dysregulated protein expression in APP/PS1 mice as assessed by mass spectrometry-based proteomics. In-depth analysis of protein expression revealed a prominent effect of SUL-138 in APP/PS1 mice on mitochondrial protein expression. SUL-138 increased the levels of proteins involved in fatty acid metabolism in both wildtype and APP/PS1 mice. Additionally, in APP/PS1 mice only, SUL-138 increased the levels of proteins involved in glycolysis and amino acid metabolism pathways, indicating that SUL-138 rescues mitochondrial impairments that are typically observed in AD.

**Conclusion:**

Our study demonstrates a SUL-138-induced shift in metabolic input towards the electron transport chain in synaptic mitochondria, coinciding with increased synaptic plasticity and memory. In conclusion, targeting mitochondrial bioenergetics might provide a promising new way to treat cognitive impairments in AD and reduce disease progression.

**Supplementary Information:**

The online version contains supplementary material available at 10.1186/s13195-022-01127-z.

## Background

Alzheimer’s disease (AD) is the most prevalent neurodegenerative disease worldwide, with numbers only expected to rise as the population ages [1]. Up till now, no effective cure has been found. Increasing evidence supports the mitochondrial cascade hypothesis of AD, proposing a cumulative decrease in mitochondrial fitness with excess reactive oxygen species (ROS) and decreased ATP production as important contributors to pathophysiological protein aggregation, decreased synaptic plasticity, and cognitive decline [2, 3]. This hypothesis is corroborated by multiple studies in AD cell culture models, animal models, and human postmortem brain tissue that link reduced mitochondrial complex activity and abundance (particularly of complex I/IV) and bio-energetic imbalance (ROS/ATP) to AD [4–8]. In addition, altering metabolic input to mitochondria by caloric restriction, intermittent fasting, and ketogenic diets have shown mild beneficial effects in animal models of neurodegenerative disorders and in human clinical trials [9, 10]. For this reason, mitochondria should be seriously considered as pharmacotherapeutic targets in the treatment of AD. So far, research has primarily focused on small molecule compounds with antioxidant (i.e., ROS scavenging) functions, such as vitamin E and curcumin. This research has shown some promising results in vitro and in mouse models of AD but fail to restore cognitive function in clinical studies, possibly due to poor blood-brain-barrier permeability and the singular effect of ROS scavenging [11, 12].

Interestingly, hibernators show exceptional plasticity in the brain during bouts of hypo-metabolism named torpor. Mitochondria shut down during torpor whereas arousals restore mitochondrial activity. These changes coincide with extensive structural and functional neuronal plasticity in the hippocampus, ranging from changes in synaptic plasticity during daily torpor in mice to complete dendritic retraction and restoration in seasonal hibernators [13–18]. Inspired by the effects of hibernation on mitochondria, and the associated boost in neuronal plasticity, 6-chromanol derived small molecules were developed that mimic the action of endogenous mediators conferring organ protection in hibernation [19–21] by phenotypical screening of cooled and rewarmed cells [22]. Follow-up research demonstrated the 6-chromanol derivatives, including SUL-138, to preserve mitochondrial respiratory chain function by supporting complexes I and IV function, thereby preventing ROS formation while stimulating ATP production [23]. SUL-138 does so without affecting basal mitochondrial membrane potential or causing apparent mitochondrial toxicity. Moreover, 6-chromanols preclude organ damage in various preclinical models of conditions or diseases with impaired mitochondrial function, including whole body cooling [24], renal ischemia/reperfusion [22], COPD [25, 26], and diabetes [27]. Lastly, SUL-138 is able to pass the blood-brain-barrier.

Given its biological and pharmacological properties, we hypothesized that SUL-138 can counteract energetic imbalance in AD. In this study, we tested the effects of SUL-138 at the molecular, physiological, and behavioral level in an APP/PS1 mouse model of AD and in healthy wildtype controls. We show that 3 months of oral administration of SUL-138 increases synaptic transmission and memory performance in both APP/PS1 and wildtype controls. This was accompanied by a substantial decrease in amyloid plaque load and a partial rescue of AD-associated changes in protein expression in the brain of SUL-138-treated APP/PS1 mice. Treatment with SUL-138 induced a significant upregulation of mitochondrial metabolic proteins involved in fatty acid degradation and oxidation in particular, while having only a modest effect on synaptic protein levels. Collectively, these data suggest that SUL-138 confers long-term metabolic adaptations in mitochondria, resulting in increased synaptic transmission and memory performance. This illustrates that targeting mitochondrial bioenergetics is a promising strategy to prevent cognitive impairment in AD.

## Results

### SUL-138 rescues memory and LTP deficits and reduces Aβ plaque load in APP/PS1 mice

**Fig. 1:**
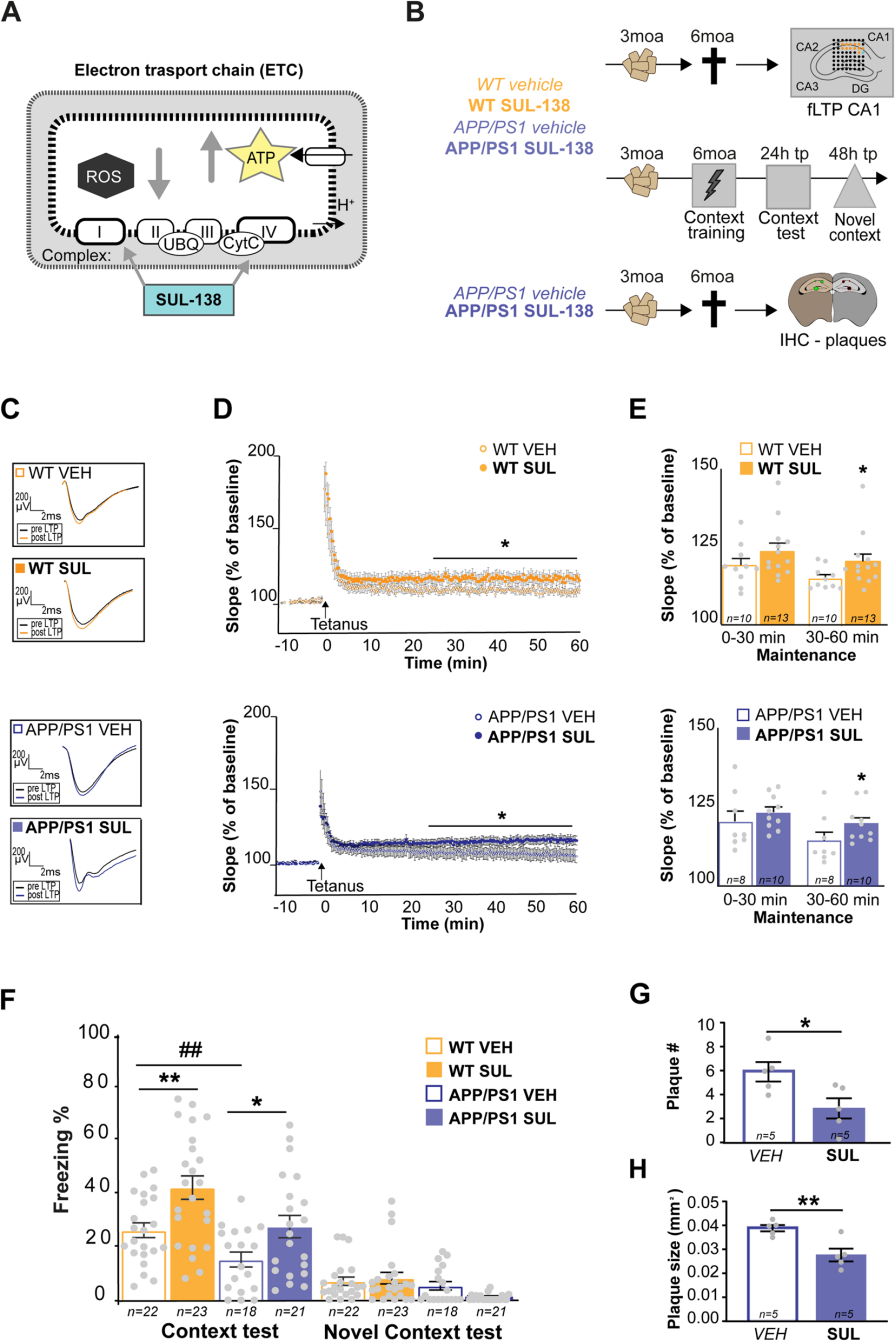
SUL-138 treatment rescues memory and LTP deficits in APP/PS1 mice. **A** SUL-138 increases mitochondrial electron transport chain complexes I (type I NADH dehydrogenase) and IV (cytochrome c oxidase) activity and efficiency, decreasing reactive oxygen species (ROS) and increasing ATP levels [23]. **B** Wildtype (WT) and APP/PS1 mice were treated with vehicle- or SUL-138-containing food pellets from 3 to 6 months of age after which conditioned fear memory or long-term potentiation (LTP) was measured and amyloid plaques were detected using immunohistochemistry (IHC) in APP/PS1 mice. **C** Representative pre- (black) and post-tetanus (orange/purple) fEPSP traces for control and SUL-138-treated WT and APP/PS1 mice. **D** LTP was measured as the fEPSP slope as percentage of baseline for control (open dot) and SUL-138-treated (solid dot) WT (orange) and APP/PS1 (purple) mice (Student’s *t*-test, *p ≤ 0.05). **E** LTP maintenance at 30–60 min after tetanus was significantly higher in SUL-138-treated compared to control mice in both WT (121.00 ± 4.67 vs 110.90 ± 2.24) and APP/PS1 (110.90 ± 5.74 vs 123.40 ± 3.99) mice (WT VEH *n* = 10, WT SUL *n* = 13, APP VEH *n* = 8 and APP SUL *n* = 10; Student’s *t*-test, *p ≤ 0.05). **F** Freezing levels after re-exposure to the context were significantly lower in vehicle-treated APP/PS1 mice compared to vehicle-treated WT mice (13.85 ± 2.54 vs 23.78 ± 2.49; WT VEH *n* = 22, APP VEH *n* = 18; one-way ANOVA, post hoc Fisher’s LSD; ^##^ *p* ≤ 0.01), confirming a memory impairment in APP/PS1 mice. SUL-138 treatment increased memory in WT mice (38.28 ± 3.98 vs 23.78 ± 2.49; WT SUL *n* = 23; one-way ANOVA, post-hoc Fisher’s LSD, **p ≤ 0.01) and rescued memory in APP/PS1 mice up to WT levels (13.85 ± 2.54 vs 25.04 ± 3.80; APP SUL *n* = 21; one-way ANOVA, post hoc Fisher’s LSD, *p ≤ 0.05). Little to no freezing was observed in the novel context (NC). Plaque number (**G**) and plaque size (**H**) in the hippocampus (2 slices per animal, mean of 4 hippocampi) were significantly lower in SUL-138-treated APP/PS1 mice compared to vehicle-treated APP/PS1 mice (*n* = 5; 2.800 ± 0.8456 vs 6.100 ± 1.719 and 0.028 ± 0.0028 vs 0.040 ± 0.0014 respectively; Student’s *t*-test, **p ≤ 0.01, *p ≤ 0.05)

We hypothesized that supporting mitochondrial bioenergetics is sufficient to restore synaptic plasticity in the hippocampus of APP/PS1 mice. To test this, we used the 6-chromanol SUL-138, which enhances the efficiency of mitochondria complex 1 (type I NADH dehydrogenase) and complex 4 (cytochrome c oxidase) of the electron transport chain, reducing ROS and increasing ATP production (Fig. 1A) [23]. APP/PS1 mice and wildtype littermates were treated orally with SUL-138 or vehicle for three months (between 3 and 6 months of age) and conditioned fear memory and hippocampal long-term potentiation (LTP) were subsequently measured (Fig. 1B). SUL-138 plasma concentrations demonstrated high oral uptake (Supplementary Table 1). SUL-138 treatment increased LTP in the CA1 region of the hippocampus, particularly during the maintenance phase at 30-60 min after LTP induction, both in wildtype (*p* = 0.0463, *t* = 1.762, df = 21, Student’s *t*-test) and APP/PS1 mice (*p* = 0.0421, *t* = 1.842, df = 16, Student’s *t*-test) (Fig. 1C–E, S1). Conditioned fear memory was impaired in APP/PS1 mice compared to wildtype at 6 months of age (Fig. 1F, *p* = 0.0453, *t* = 2.033, df = 80, one-way ANOVA, post hoc Fisher’s LSD), as observed in previous studies [28, 29]. In APP/PS1 mice, SUL-138 treatment rescued contextual fear memory to wildtype levels (*p* = 0.0261, *t* = 2.267, df = 80, one-way ANOVA, post hoc Fisher’s LSD). Interestingly, SUL-138 treatment also increased contextual fear memory in wildtype mice (*p* = 0.0022, *t* = 3.162, df = 80, one-way ANOVA, post hoc Fisher’s LSD). None of the groups showed substantial freezing in a novel context and basal locomotor activity was the same in SUL-138-treated and vehicle-treated wildtype and APP/PS1 mice (WT VEH vs WT SUL: *p* = 0.8922, t = 0.1359, df = 79 and *p* = 0.8918, t = 0.1365, df = 79; APP VEH vs APP SUL: *p* = 0.2462, t = 1.523, df = 79 and *p* = 0.2468, t = 1.522, df = 79 ; one-way ANOVA; Figure S2).

**Fig. 2:**
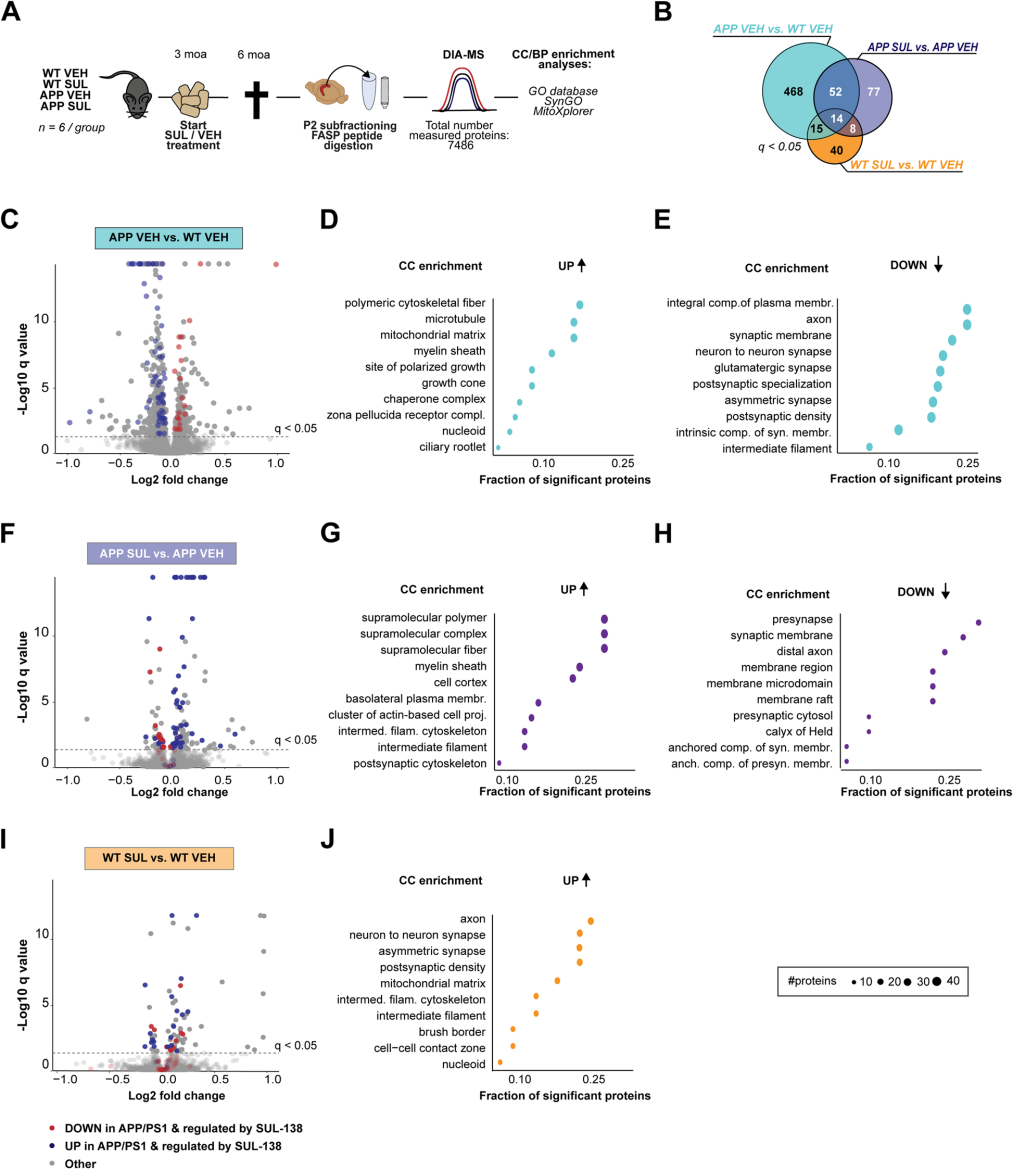
Proteomic analysis of SUL-138 treatment in APP/PS1 and wildtype mice. **A** Following 3 months of vehicle (VEH) or SUL-138 (SUL) treatment of wildtype (WT) and APP/PS1 mice (*n* = 6 per group), hippocampal P2 protein samples were prepared, FASP-digested, and used for quantitative DIA mass spectrometry. In total, 7486 proteins were measured. Data was further analyzed using gene ontology (GO) enrichment for cellular components (CC) and biological processes (BP) against the GO, SynGO, and MitoXplorer databases. **B** Differential protein expression was determined for three contrasts, APP VEH vs. WT VEH (blue), APP SUL vs. APP VEH (purple), and WT SUL vs. WT VEH (orange), resulting in the indicated numbers of significant differentially expressed proteins (FDR, *q* ≤ 0.05). **C** In the APP VEH vs. WT VEH comparison, most proteins are significantly downregulated (365 down vs. 184 up, FDR *q* ≤ 0.05) in APP/PS1 mice. Proteins that are dysregulated in APP/PS1 and are regulated in SUL treated APP/PS1 or WT are highlighted in red when they are upregulated and blue when they are downregulated in APP VEH vs. WT VEH. **F** In the APP VEH vs. APP SUL comparison, most proteins are significantly upregulated (109 up vs. 42 down, FDR *q* ≤ 0.05) after SUL-138 treatment. Proteins that are dysregulated in APP/PS1 are highlighted in red when they are upregulated and blue when they are downregulated in APP VEH vs. WT VEH to illustrate directionality of SUL-138 regulation. **I** In the WT VEH vs. WT SUL comparison, most proteins are significantly upregulated (60 up vs. 17 down, FDR *q* ≤ 0.05) after SUL-138 treatment. Proteins that are dysregulated in APP/PS1 and are regulated in SUL treated WT are highlighted in red (upregulated in APP VEH vs. WT VEH) and blue (downregulated in APP VEH vs. WT VEH) to illustrate directionality of SUL-138 regulation. **D**, **E**, **G**, **H**, **J** Top 10 enriched CC GO terms for upregulated (top panel) and downregulated (lower panel) proteins for each of the 3 contrasts. Size of the dots represents the number of proteins annotated to the GO term and the fraction of significant proteins is the number of significant proteins divided by the total number of proteins belonging to that term

Next, we assessed the effect of three months of SUL-138 treatment on a key pathological hallmark of AD, the formation of amyloid plaques, in the hippocampus of APP/PS1 mice (Fig. 1G/H, S3). SUL-138 treatment decreased both number (by 54%) and size (by 30%) of plaques compared to vehicle treated APP/PS1 mice (*p* = 0.0203 and *p* = 0.0042, Student’s *t*-test; average of the 4 hippocampi of 2 slices/animal) (Fig. 2B–D, S3A&B). This decrease in plaque load seemed not to be due to alterations in amyloid clearance as we did not observe changes in GFAP or Iba1 expression upon SUL-138 treatment (Figure S4). Taken together, these data show that SUL-138 increases synaptic plasticity and memory in mice to a level that is sufficient to rescue impairments in APP/PS1 mice and is able to prevent pathological amyloid accumulation.

### SUL-138 treatment alters protein levels in hippocampal synapse-enriched P2 fractions

We subsequently investigated the effects of SUL-138 treatment on hippocampal protein expression to uncover potential mechanisms underlying enhanced plasticity and reduced amyloid plaque load. We used mass spectrometry to quantify protein levels in hippocampal P2 fractions, enriched for synapses, followed by differential expression analysis (DEA; see methods, Supplementary dataset 1) in vehicle (VEH) or SUL-138 (SUL) treated APP/PS1 (APP) and wildtype (WT) mice. The total number of detected proteins amounted 7846 (Fig. 2A). Differential protein expression was determined for selected pairwise comparisons; APP VEH vs. WT VEH to detect protein dysregulation due to disease and APP SUL vs. APP VEH and WT SUL vs. WT VEH to identify effects of SUL-138 on protein expression under healthy conditions (Fig. 2B). After multiple testing correction, 549 differentially expressed proteins were identified in the APP VEH vs. WT VEH comparison (FDR, *q* ≤ 0.05). After SUL-138 treatment in APP/PS1 mice, 151 proteins were differentially expressed (APP SUL vs. APP VEH; FDR, *q* ≤ 0.05), of which 66 (44%) overlap with the APP VEH vs. WT VEH comparison, indicating a potential rescue of APP/PS1-altered protein expression. In SUL-138-treated wildtype mice, 77 differentially expressed proteins were identified (WT SUL vs. WT VEH; FDR, *q* ≤ 0.05), of which 22 (28%) overlap with the APP SUL vs. APP VEH comparison, indicating that the number proteins affected by SUL-138 treatment is substantially smaller and partially differs in wildtype mice compared to APP/PS1 mice.

### Protein dysregulation in APP/PS1 mice

Figure 2C shows the protein dysregulation in the hippocampus due to disease (APP/PS1 mice compared to wildtype controls - APP VEH vs. WT VEH). Of all differentially regulated proteins, most are downregulated in APP/PS1 mice (365/549; FDR, *q* ≤ 0.05) and cellular component (CC) GO enrichment analysis [30] revealed many synaptic terms, e.g., “glutamatergic synapse,” “postsynaptic density,” and also “intermediate filament” (Fig. 2E), whereas biological process (BP) terms point to “neuron projection morphogenesis,” “transsynaptic signaling,” and “actin filament organization” (supplementary dataset 2, Figure S5A, lower panel). Focusing on the 184 significantly upregulated proteins (FDR *q* ≤ 0.05), CC enrichment analysis reveals enrichment of, e.g., “mitochondrial matrix” and “chaperone complex” (Fig. 2D) and “protein folding” and “nucleotide metabolic process” (supplementary dataset 3). In addition, BP terms such as “amyloid beta formation,” “amyloid precursor protein catabolic process,” and “amyloid fibril formation” are enriched, as is expected in this mouse model (Figure S5A, upper panel).

In total, 15.2% of measured known AD-associated proteins (22 of 138 measured UniProt “Alzheimer human” proteins [31] and 5 out of the 18 measured GWAS gene-encoded proteins [32, 33]; 27.8%) are significantly altered in APP VEH mice. These are APBA1, APOE, APP, BIN1, CLU, CTSD, DBN1, DNM1L, GFAP, GPC1, HSD17B10, IDE, LMNA, LMNB1, MAP2, NPEPPS, OGT, PRNP, PSEN1, SNCB, SOD2, and SYNPO for UniProt and APH1B, APP, APOE, BIN1, and CLU for GWAS (Figure S6A). These findings confirm the AD-like molecular phenotype of APP/PS1 mice.

### Effects of SUL-138 treatment in APP/PS1 mice

Figure 2F shows hippocampal protein regulation as a result of 3 months of SUL-138 treatment in APP/PS1 mice (APP SUL vs. APP VEH). Most proteins (109/151) are upregulated in APP/PS1 mice after SUL-138 treatment and CC enrichment analysis shows that these proteins belong to, e.g., “supramolecular complex,” “postsynaptic cytoskeleton,” and “intermediate filament” and to the BP terms “cytoskeleton organization,” “fatty acid oxidation,” and “carboxylic acid metabolic process” (Fig. 2G and S5B, upper panel; supplementary dataset 4). Downregulated proteins (42/151) show enrichment of “presynapse,” “synaptic membrane,” and “presynaptic cytosol” (Fig. 2H and S3B, lower panel; supplementary dataset 5), which include the AD-associated proteins APP, CLU, MAP2, and serine/threonine-protein kinases A, B, and G.

Overall, 66 of 151 significantly SUL-138-regulated proteins overlap with proteins affected in APP/PS1 mice (Fig. 2B, F). Of these 66 proteins, 56 (10.2% of all 549 APP/PS1-affected proteins) are significantly rescued, i.e., regulated in the opposite direction (blue vs. red highlight), of which enrichment analyses revealed that they belong to, e.g., “supramolecular fiber,” “axon,” and “intermediate filament cytoskeleton” and to the BP terms “neuron projection morphogenesis,” “supramolecular fiber organization,” and “intermediate filament−based process” (Figure S7A&B; supplementary dataset 6). Furthermore, SUL-138 treatment of APP/PS1 mice induced an opposite direction of regulation of the AD-associated proteins APP, CLU, IDE, MAP2, LMNB1, LMNA, and GFAP (Figure S6B) compared to the APP VEH vs. WT VEH contrast (Figure S6A), indicating that SUL-138 treatment partially precludes AD-associated protein expression. In addition, SUL-138 treatment significantly affected the levels of the AD-associated proteins ACE, GOT1, RBM25, and RGS6, even though they were not significantly changed in APP/PS1 mice.

Taken together, these data show that SUL-138 partially prevents AD-associated protein dysregulation in APP-PS1 mice and restores the levels of several postsynaptic cytoskeletal proteins. GO enrichment analyses further points to mitochondrial metabolic processes as possible targets of SUL-138 in APP/PS1 mice.

### Effects of SUL-138 treatment in wildtype mice

Figure 2I shows the hippocampal proteome changes in SUL-138-treated wildtype mice. SUL-138 changed the levels of 77 proteins in wildtype animals which is less than the 151 regulated proteins in APP/PS1 mice (FDR, *q* ≤ 0.05). Differentially expressed proteins were mostly upregulated (60/77), with a significant CC GO term enrichment for “axon,” “mitochondrial matrix,” and “intermediate filament cytoskeleton” (Fig. 2J). BP enrichment analysis revealed “mitochondrial fatty acid beta oxidation” as the most enriched process (supplementary dataset 7, Figure S5C, upper panel). Downregulated proteins (17/77) showed no significant CC enrichment and BP enrichment for “glycogen catabolic process” and interestingly “amyloid beta clearance” and “amyloid beta metabolic processes,” due to a significant decrease in the levels of, e.g., APOE and IDE in wildtype mice (supplementary dataset 8, Figure S5C, lower panel). Of the 77 regulated proteins affected by SUL-138 treatment in wildtype mice, 29 overlap with dysregulated proteins in APP/PS1 mice (APP VEH vs. WT VEH; red and blue highlight in Fig. 2C). Eight AD-associated proteins were regulated by SUL-138 in wildtype mice: ACE, APOE, CTSD, DBN1, IDE, LMNA, PPP3CA, and SNCA (Figure S6C). Taken together, these data show that SUL-138, independent of genotype, regulates AD-associated proteins, proteins involved in metabolism and cytoskeleton proteins.

### SUL-138 does not have a major effect on synaptic protein expression

**Fig. 3:**
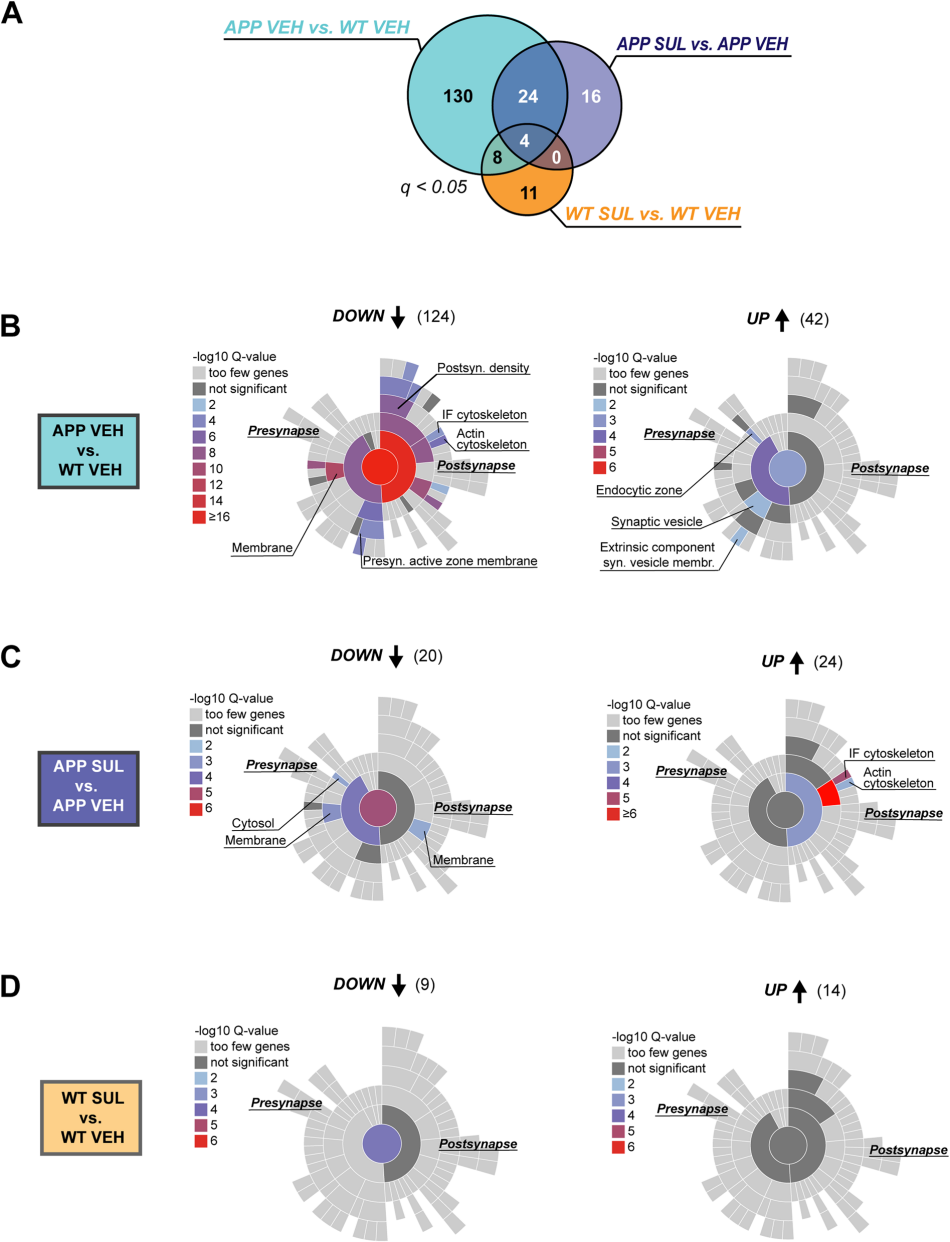
SynGO analyses of synaptic protein regulation by SUL-138 in APP/PS1 and wildtype mice. **A** Differential synaptic protein expression was determined for three contrasts, APP VEH vs. WT VEH (blue), APP SUL vs. APP VEH (purple), and WT SUL vs. WT VEH (orange), resulting in the indicated numbers of significant differentially expressed proteins (FDR, *q* ≤ 0.05). **B** Sunburst plots of CC enrichment for significantly downregulated proteins in APP VEH vs. WT VEH using SynGO showing significant enrichment of multiple pre- and post-synaptic GO terms. Upregulated proteins were annotated to the presynaptic endocytic zone and to synaptic vesicles. **C** Sunburst plots of CC enrichment for significantly downregulated proteins in APP SUL vs. APP VEH reveals modest enrichment for proteins belonging to the pre- and postsynaptic membrane and presynaptic cytosol. Upregulated proteins show a significant enrichment for postsynaptic intermediate filament (IF) and actin cytoskeleton proteins. **D** Sunburst plots of CC enrichment for significantly down- and upregulated proteins in WT SUL vs. WT VEH do not show any specific enrichment of synaptic GO terms

The synaptic signature of the GO enrichment analyses together with the effects of SUL-138 on synaptic plasticity (Fig. 1C–E) prompted us to investigate synaptic protein expression in more detail using in-depth synaptic functional annotation (SynGO, Fig. 3), a dedicated and curated synapse ontology database [34]. In total, 166 regulated proteins in the disease model (APP VEH vs. WT VEH) were annotated in SynGO, while SUL-138 treatment altered 44 synaptic proteins in APP/PS1 mice (APP SUL vs. APP VEH). Furthermore, SUL-138 treatment of wildtype mice led to regulation of 23 synaptic proteins (WT SUL vs. WT VEH; Fig. 3A). The SynGO annotation shows that downregulated proteins in APP/PS1 mice contain a wide spectrum of synaptic proteins belonging to both the pre- and the postsynaptic domain (Fig. 3B, Figure S8A, Supplementary dataset 9). Upregulated proteins show some enrichment of presynaptic proteins, in particular endocytic zone and synaptic vesicle proteins, including several AD-associated proteins, i.e., APP, APBA1, CTSD, BIN1, DNM1L, OGT, PSEN1, and SNCB.

SUL-138 treatment in APP/PS1 mice had a very modest effect on postsynaptic protein expression, increasing the levels of several actin and intermediate filament cytoskeletal proteins, specifically INA, NEFH, NEFL, NEFM and MYH10, MYO6, and SYNE, and decreasing the levels of some presynaptic cytosol and membrane proteins, which were primarily AD-associated proteins again, i.e., APP, CLU, MAP2, and GPC4, and serine/threonine-protein kinases A, B, and G (Fig. 3C and S8B, Supplementary dataset 10).

In wildtype mice, SUL-138-induced changes in protein levels were not associated with any specific SynGO terms (Fig. 3D and S8C, Supplementary dataset 11). Together, these data suggest that the increase in synaptic plasticity by SUL-138 treatment is not due to specific changes in synaptic protein levels other than a few actin and intermediate filament proteins.

### SUL-138 differentially affects the mitochondrial proteome in APP/PS1 and wildtype mice

**Fig. 4:**
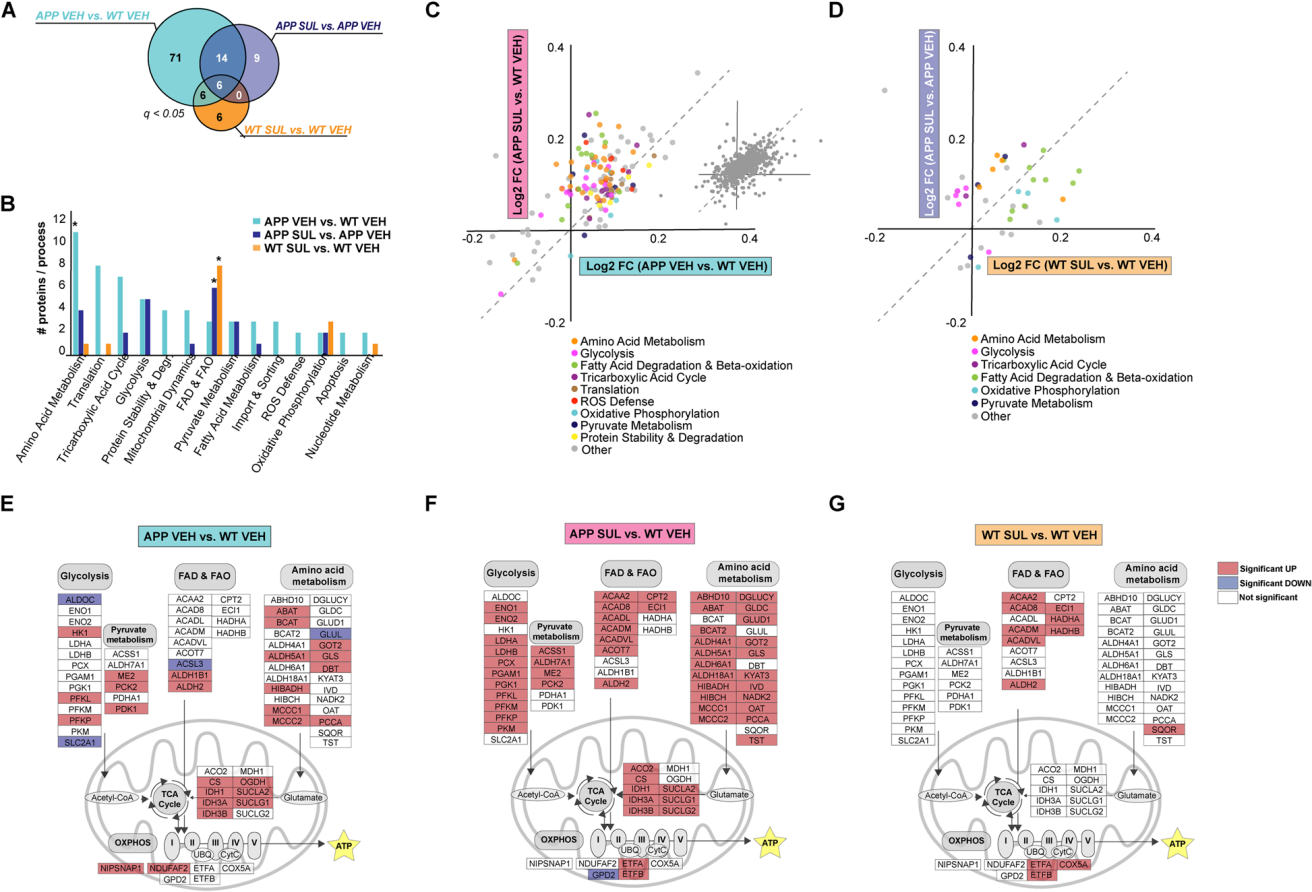
SUL-138 shifts the metabolic proteome. **A** Venn diagram of significantly regulated mitochondrial proteins for the three relevant comparisons APP VEH vs. WT VEH, APP SUL vs. APP VEH and WT SUL vs. WT VEH (FDR, *q* ≤ 0.05). **B** MitoXplorer analysis of all mitochondrial proteins with significant regulation in at least one comparison. The graph indicates the number of proteins per mitochondrial process for each comparison; significant MitoXplorer enrichment is indicated (*p ≤ 0.05). **C** Pairs contrast graph of differential mitochondrial protein expression in APP VEH vs. WT VEH (*x*-axis) and APP SUL vs. WT VEH (*y*-axis) comparisons. A general upregulation of mitochondrial proteins involved in multiple metabolic processes is observed in APP/P1 mice (APP VEH vs. WT VEH), whereas only the levels of proteins involved in amino acid metabolism, FAD and FAO and glycolysis are further increased by SUL-138 treatment. The inset shows expression levels of all mitochondrial proteins, without statistical cut-off, and indicates that the observed upregulation of several mitochondrial processes is not due to a general upregulation of all mitochondrial proteins. **D** Pairs contrast graph of differential mitochondrial protein expression in WT SUL vs. WT VEH (*x*-axis) and APP SUL vs. APP VEH (*y*-axis) comparisons showing a clear separation of mitochondrial protein regulation, with FAD and FAO regulated more strongly in WT mice and amino acid metabolism and glycolysis in APP/PS1 mice. **E**–**G** Schematic summary of genotype and SUL-138 treatment on the three main metabolic inputs (glycolysis, FAD and FAO, and amino acid metabolism) towards the TCA cycle and the oxidative phosphorylation (OXPHOS) complex. In red and blue significantly upregulated and downregulated proteins (FDR, *q* ≤ 0.05) are indicated

As our primary GO analyses pointed to mitochondrial metabolic process being dysregulated in APP/PS1 mice as well as being regulated by SUL-138 treatment in both APP/PS1 and wildtype mice, we next performed an in-depth analysis of mitochondrial protein expression using MitoXplorer, a tool that specifically enables the functional annotation of mitochondrial proteins [35]. In each of the three comparisons, a substantial number of MitoXplorer-annotated mitochondrial proteins were significantly differentially expressed (97 for APP VEH vs. WT VEH, 29 for APP SUL vs. APP VEH and 18 for WT SUL vs. WT VEH; FDR, *q* ≤ 0.05) (Fig. 4A). The APP/PS1 genotype induced a significant enrichment of proteins involved in amino acid metabolism (APP VEH vs. WT VEH). In contrast, SUL-138 treatment induced a significant enrichment for fatty acid degradation (FAD) and fatty acid beta oxidation (FAO) in both APP/PS1 and WT mice (APP SUL vs. APP VEH and WT SUL vs. WT VEH) (Fig. 4B; supplementary dataset 12), suggesting that SUL-138 shifts energy substrate usage.

Examining in more detail mitochondrial protein dysregulation in APP/PS1 mice (APP VEH vs. WT VEH) and the effect of SUL-138 (APP SUL vs. WT VEH), we plotted the fold changes of all mitochondrial proteins that are significantly changed in at least one comparison (FDR, *q* ≤ 0.05) in a pairs contrast graph (Fig. 4C), showing a specific upregulation of several mitochondrial processes in APP/PS1 mice, which is further enhanced by SUL-138. The upregulation of these proteins does not seem to be due to an overall increase in mitochondrial abundance as it is not observed for all mitochondrial proteins (inset in Fig. 4C). Thus, rather than restoring mitochondrial protein expression in APP/PS1 mice, SUL-138 selectively enhances the expression of proteins involved in specific mitochondrial pathways, in particular the amino acid metabolism, FAD and FAO and glycolysis pathways. When the effects of SUL-138 in APP/PS1 mice (APP SUL vs. APP VEH) and in wildtype mice (WT SUL vs. WT VEH) are compared in the same way, a clear and interesting separation between SUL-138 effects can be observed (Fig. 4D). Proteins involved in FAD and FAO are more strongly upregulated by SUL-138 in wildtype mice, whereas upregulation of glycolysis and amino acid metabolism proteins is more pronounced in APP/PS1 mice. Figure 4E, G (& S9) summarizes the changes in mitochondrial metabolic pathways in APP/PS1 mice and as a result of SUL-138 treatment. Whereas proteins involved in FAD and FAO are upregulated by SUL-138 in both wildtype and APP/PS1 mice, upregulation of glycolysis and amino acid metabolism proteins is only observed in APP/PS1 mice. In addition to the general effects of SUL-138 on FAD and FAO, electron transfer flavoprotein subunit alpha and beta (ETFA and B), which transfer electrons from FAD and FAO to the ubiquinone pool of the oxidative phosphorylation complex in the electron transport chain, are upregulated in both APP/PS1 and wildtype mice. Together, these results indicate that SUL-138 treatment shifts mitochondrial energy production to FAD and FAO in general and increases glycolysis, amino acid metabolism, and oxidative phosphorylation in APP/PS1 mice specifically.

## Discussion

We show that three months of treatment with SUL-138 rescues LTP and memory deficits and decreases amyloid-beta plaque load in APP/PS1 mice. Proteomic analysis of synapse-enriched P2 fractions revealed that SUL-138 partially rescued dysregulated protein expression in the hippocampus of APP/PS1 mice, including several typical AD-associated proteins. SUL-138 did not prominently affect synaptic protein levels, but profoundly affected protein levels in several main mitochondrial metabolic pathways in both APP/PS1 and wildtype mice. Our findings indicate that a shift in mitochondrial metabolism may contribute to the enhancement of synaptic plasticity in a way that is relevant for the treatment of AD.

APP/PS1 mice are widely used as an amyloid-driven AD mouse model and display several hallmarks of the disease, including synaptic and cognitive impairment and amyloid plaque accumulation [36, 37]. Indeed, we found an impairment of hippocampal LTP and memory and detected abundant amyloid plaque deposition in the hippocampus at 6 months of age. We further demonstrate that differentially expressed proteins in the hippocampus of APP/PS1 compared to wildtype mice at this age include a substantial number of known human AD-associated proteins (15% and 28% of Uniprot proteins and GWAS identified genes, respectively), which may be surprising given the fact that no mouse model of AD fully recapitulates the human disease [38]. The relevance of the APP/PS1 model is further substantiated by GO analyses demonstrating enrichment of GO terms reflecting amyloid beta formation, precursor protein catabolism and fibril formation. Functional enrichment analyses also identified synaptic and mitochondrial metabolic protein dysregulation in APP/PS1 mice, both of which are hallmarks of AD pathology. Synaptic proteins were overall downregulated in APP/PS1 mice, supporting previously observed synapse loss in AD [39]. On the other hand, APP/PS1 mice feature an upregulation of mitochondrial proteins involved in amino acid metabolism, possibly to compensate for functional mitochondrial impairments in AD and to maintain energy homeostasis [40]. Noteworthy, APP/PS1 mice also showed an upregulation of 7 out of 8 chaperonin-containing tailless complex polypeptides (i.e., CCT2-8), which are known to regulate protein aggregation and were shown to be lower expressed in AD brains [41]. This upregulation may serve as a compensatory mechanism against pathogenic amyloid-beta aggregation [42] and provides an interesting new link towards controlling amyloid-beta aggregation in AD. One possible limitation of our findings is that only male mice were used. It is well-known that changes in the levels of sex steroids in female mice have an effect on hippocampal plasticity [43], and the inclusion of female mice would therefore have reduced the statistical power to detect SUL-138-induced changes. Similar to male mice, female APP/PS1 mice also show increased Aβ levels and learning and memory impairments [44, 45]. Future experiments should investigate the beneficial effects of SUL-138 in female mice in order to provide better clinical translation of our findings.

Several of the AD-associated proteins that are regulated by SUL-138 have clear connections to amyloid plaque pathology. Cleavage of APP leads to the production of Aβ peptides which are the basis of amyloid plaques [46] and clusterin (CLU), which mediates Aβ toxicity [47], is found increased in AD patient brains [47]. Insulin degrading enzyme (IDE; also known as amyloid-beta degrading protease) has been shown to be important for amyloid clearance and IDE levels are decreased in AD patients [48]. Also, levels of angiotensin-converting enzyme (ACE), which functions to degrade Aβ, are lower in AD patients [49]. Together, these SUL-138-induced changes in AD-associated protein levels may (in part) be responsible for the decreased amyloid plaque load that we observed in SUL-138-treated APP/PS1 mice. Whether the rescue of AD pathology by SUL-138 is a direct effect or a secondary effect of changes in mitochondrial energy balance remains to be determined.

The most prominent effect of SUL-138 treatment was a genotype-independent upregulation of mitochondrial fatty acid degradation and fatty acid beta oxidation pathways and of electron transfer flavoprotein subunit alpha and beta protein expression. The first convert fatty acids into direct substrates for oxidative phosphorylation, while the latter transfer electrons from these substrates to the electron transport chain via the ubiquinone pool. This pathway promotes protonmotive force, thereby stimulating ATP synthesis [50, 51]. The SUL-138-induced increase in protein levels in this pathway in both APP/PS1 and wildtype mice could therefore potentially underlie the observed increase in ATP-dependent LTP and memory in both genotypes [52, 53]. In addition, fatty acid beta oxidation has been shown to directly alter hippocampal plasticity, i.e., by stimulating adult neuronal stem cell activity [54]. Besides these genotype-independent changes, SUL-138 increased the levels of glycolysis and amino acid metabolism proteins in APP/PS1 mice specifically. This could reflect the inability of mitochondria in APP/PS1 mice to meet ATP demand due to an obstruction in fatty acid oxidation and glycolysis, both of which are impaired in AD [55–57].

It has been shown previously that SUL-138 also increases the activity of complexes I and IV [23]. The same complexes are upregulated during daily torpor in mice [18]. This points to an additional beneficial effect of SUL-138 by raising protonmotive force via these complexes [58]. Further studies using direct mitochondrial respiration measurements (e.g., complex efficiency, ROS levels, ATP production, oxidative phosphorylation input) are required to determine which mitochondrial pathways are functionally affected by SUL-138 and whether these align with the changes in protein expression that we observed.

Given the effects of SUL-138 on hippocampal LTP and memory we were surprised to see the relatively small effect of SUL-138 on synaptic protein expression. In APP/PS1 mice, SUL-138 treatment produced a rather specific rescue of postsynaptic proteins belonging to the actin and intermediate filament cytoskeleton, in particular INA, NEFH, NEFL, and NEFM and actin-dependent motor proteins MYH10 and MYO6. Both actin and intermediate filament proteins are crucial for dendritic spine architecture, which is tightly connected to synaptic strength [59]. Postsynaptic cytoskeletal proteins have previously been linked to LTP and memory [60–62], and their loss in AD has been observed [63–65]. However, we did not find significant changes in the levels of neurotransmitter receptors or related signaling proteins that would be required to translate synaptic input into structural change, and it seems unlikely that changes in actin and intermediate filament proteins alone are sufficient to explain the observed changes in synaptic plasticity and memory.

Interestingly, mitochondrial distribution is also dependent on the cytoskeleton and essential for synaptic activity, as mitochondria produce ATP, maintain Ca^2+^ homeostasis and support activity-dependent plasticity via local translation of proteins [52, 66]. Several cytoskeleton proteins identified in our study are thought to be responsible for the transport and docking of mitochondria, e.g., motor proteins MYH10 and MYO6, and actin is the major cytoskeleton player governing mitochondrial arrest in synapses [59, 66]. It is thus conceivable that SUL-138 not only affects mitochondrial metabolism but also promotes directly or indirectly the transport and docking of mitochondria to synapses, thus contributing to energy-dependent synaptic plasticity and enhancing LTP and memory.

The significance of targeting mitochondrial bioenergetics for neuronal protection is supported by studies on pioglitazone, which acts via mitoNEET on oxidative phosphorylation [67] and on MitoQ, which works via antioxidant support of complex II [68]. These compounds show promising outcomes in various neurodegenerative disease models, including AD mouse models such as 3xTg-AD, APP/PS1, and APP-J20 [68, 69]. However, just as other ROS scavengers, clinical outcomes have not met expectations yet [70, 71]. In addition, growing evidence is showing that ketone bodies, the derivatives of fatty acid oxidation, have broad neuroprotective effects [72] and that ketogenic diets cause modest cognitive improvement in AD patients, further supporting fatty acid metabolism as a promising therapeutic target.

## Conclusions

The hibernation-derived substituted 6-chromanol SUL138 was previously shown to support electron transport and limit ROS production [23] and protect against cooling-induced [24] and diabetic [27] kidney injury. Our data add to these findings by demonstrating SUL-138 prompts fatty acid metabolism in mitochondria, enhances synaptic plasticity and memory, and reduces amyloid-beta plaque load in APP/PS1 mice. The dual effect of SUL-138, both decreasing ROS levels and shifting towards more efficient ways of producing ATP, may be of particular relevance for compromised neurons in the treatment of AD.

## Materials and methods

### Animals

All experiments with animals were approved by the local animal research committee (IVD) and complied with the national central committee of animal research (CCD #16427) ordination. APP/PS1 (strain B6C3-Tg(APPswe,PSEN1dE9)85Dbo/J) mice were bred locally in the Amsterdam Animal Research Center (AARC). Male animals were used in all experiments. APP/PS1 mice were 3 months ± 2 weeks of age at the start of the experiment. Mice were single-housed on sawdust in standard Makrolon type 2 cages (~21 °C ambient temperature (*T*_a_) and ~50% humidity), enriched with cardboard nesting material and chewing wood and with food and water ad lib. Mice were kept on a 12:12 light-dark cycle, with lights on at 7:00 AM.

### SUL-138 treatment

Mice were fed ad lib with vehicle or SUL-138 containing food pellets to reach a desired oral dose of 30 mg/kg/day. Based on mouse weight of ~30g, food intake of ~5 g/day and a desired oral intake of SUL-38 of 30mg/kg/day, food pellets were sprayed with SUL-138 (1g per 5kg food) dissolved in alcohol and diluted with water to a final concentration of 0.015 % ethanol. Vehicle food was prepared by spraying with the same volume of 0.015 % ethanol containing water. Blood SUL-138 titer was determined after 1 week of treatment (Supplementary Table 1). Treatment was continued for ~12 weeks until fear conditioning, electrophysiology, and until decapitation to obtain frozen hippocampi for molecular analysis or perfusion with 4% paraformaldehyde (PFA) for brains used for immunohistochemical analyses.

### Long-term potentiation

Field long-term potentiation (LTP) was recorded using a planar multi-electrode recording setup (MED64 system; Alpha Med Sciences, Tokyo, Japan). Animals were decapitated and brains were immediately placed in ice-cold slicing buffer (124 mM NaCl, 3.3 mM KCl, 1.2 mM KH_2_PO_4_, 7 mM MgSO_4_, 0.5 mM CaCl_2_, 20 mM NaHCO_3_ and 10 mM glucose; constantly gassed with 95% O_2_/5% CO_2_). Coronal hippocampal slices were cut using a vibrating microtome at 400 μM and then placed in a chamber containing artificial cerebrospinal fluid (aCSF: 124 mM NaCl, 3.3 mM KCl, 1.2 mM KHPO_4_, 1.3 mM MgSO_4_, 2.4 mM CaCl_2_, 20 mM NaHCO_3_ and 10 mM glucose; constantly gassed with 95% O_2_/5% CO_2_). Slices were left in the buffer for at least 30 min before recording. The slices were placed on an 8 × 8 multi-electrode array containing P5155 probes (Alpha Med Sciences; inter-electrode distance 150 μm), and 500 μL aCSF was added to the moist chamber which was constantly gassed with 95%O_2_/5% CO_2_. Correct placement of the array over the CA1 area was done using a microscope (SZ61, Olympus, Japan), and an image of the placement was acquired for all the recorded slices. Slices were held in place using a platinum harp. During recording, the chamber with the slice was constantly perfused with oxygenated aCSF at flow rate 2 mL/min at RT. From the 64 electrodes, one electrode on the afferent side of the CA1 area was chosen for stimulation. All other electrodes recorded, but only the electrodes that were in the direct lane of stimulation were used for analysis (5–8 electrodes per recording). Slices with LTP measurements below baseline level of 100% were excluded and numbers did not differ between groups (data not shown). After maintaining a stable baseline recording for at least 10 min, LTP was evoked by 3x 100 Hz stimulation (tetanus) of 1 s separated by 20 s, and the slope and amplitude were measured for 60 min. LTP was expressed as percentage of baseline. All data collection and processing were performed blinded.

### Contextual fear conditioning

Mice were handled for 2 min on 2 consecutive days prior to conditioning. Context training was performed in a plexiglass chamber with a stainless steel grid floor in a sound-proof setup (Noldus). Mice were placed in the context and after 2 min received a 2-s 0.7 mA shock. Mice were kept in the context training box for an additional 30 s. In between mice, the box was thoroughly cleaned using 70% ethanol. During training, white noise was present. Context-dependent memory was assessed 24 h, 48 h, 72 h, and 96 h later in the same context (including white noise) by measuring freezing for 120 s. Lastly, a novel context (a triangular shaped plastic box without grid) was used to assess generalized fear 24 h after the context test. Mice were put in the novel context without white noise and freezing levels were measured for 120 s. Between mice, the novel context box was cleaned using 0.1% acetic acid. Freezing was recorded and analyzed using Ethovision XT software (Noldus). Freezing was defined as absence of movement, including nose movement, except for respiration or heartbeat movement, and expressed as percentage of time.

### Synapse enrichment for proteomics (P2)

Hippocampal tissue was homogenized in ice-cold homogenization buffer (0.32 M sucrose, 5 mM HEPES/NaOH, PH 7.4) containing 1 tablet of cOmplete™ EDTA-free Protease Inhibitor Cocktail per 50mL (Sigma-Aldrich) and 1 tablet of PhosStop per 10 mL (Sigma-Aldrich). The homogenate was then centrifuged at 1000×g for 10 min at 4 °C. The supernatant was removed and centrifuged at 20,000×g for 20 min to obtain a synapse-enriched pellet (P2).

### Protein digestion

P2 protein concentrations were determined using Bradford assay, and 25 μg protein per animal was used for FASP in-solution digestion as previously described [73]. In brief, samples were incubated with 120 μL reducing agent (2% SDS, 100 mM TRIS, 1.33 mM TBEP) at 55 °C for 1 h while shaking constantly at 900 rpm. Samples were then incubated with 2 μL methyl methanethiosulfonate for 15 min at RT while shaking. The samples were then loaded onto YM-30 filters (Microcon, Millipore), and 250 μL 8 M urea in 100 mM Tris (pH 8.8) was added. The samples were washed by spinning the filters at 14,000×g for 10 min followed by washing with fresh urea for 4x. Finally, the samples were washed with 50 mM NH_4_HCO_3_. After the washing steps the samples were incubated with trypsin overnight in a humidified chamber at 37 °C. The peptides were eluted form the filter using 0.1% acetic acid, dried in a SpeedVac, and stored at − 20 °C.

### Liquid chromatography and mass spectrometry

Peptides were quantified by LC-MS/MS using an Ultimate 3000 LC system (Dionex, Thermo Scientific) coupled to a Triple TOF 5600 mass spectrometer (Sciex). Peptides were trapped on a 5-mm Pepmap 100 C18 column (300 μm i.d., 5 μm particle size, Dionex) and fractionated on a 200 mm Altima C18 column (100 μm i.d., 3 μm particle size). The concentration of acetonitrile in the mobile phase was increased from 5 to 18% in 88 min, 18 to 25% in 98 min, 25 to 40% in 108 min, and to 90% in 2 min at a flow rate of 5 μL/min. Eluted peptides were electro-sprayed into the TripleTOF MS using a microspray needle of 5500 V. DIA experiments consisted of a parent ion scan of 150 ms followed by a DIA window of 8 Da with a scan time of 80 msec. It was stepped through the mass range between 450 and 770 m/z. The collision energy per window was based on the appropriate collision energy for 2+ ions centered upon the window with a spread of 15 eV.

6-Chromanol levels in plasma was performed using LC-MS/MS. First, plasma were mixed with acetonitrile and sonicated, followed by centrifugation at 14,000×g to pellet protein precipitate and debris. Tissue samples were subjected to additional solid phase extraction using a SPE Strata C-18 cartridge (100 mg, 55 μm, 70Å, Phenomenex, Torrance, CA), prepared with 1 ml methanol followed by 1 ml water. Analytes were eluted in acetonitrile to methanol (3:7 v/v). Analyte recovery was > 70 %. Liquid chromatography of the samples was performed on a 1260 Infinity HPLC device (Agilent Tech., Santa Clara, CA) using a ZORBAX Eclipse AAA column (3.0 × 150 mm, particle size 3.5 μm) in a reversed phase setup and a flow rate of 0.5 mL/min. Solvents consisted of methanol (6%) to acetonitrile (4%) acetate and methanol (54%) to acetonitrile (36%) in water with 0.1% ammonium for solvent A and B, respectively. MS/MS detection was performed on a QQQ 6460 mass spectrometer (Agilent Tech., Santa Clara, CA). Detection was set for a quantifier ion (205.1 m/z, collision energy 25V). Gas temperature for MS was set to 300 °C and flow was set to 6 L/min. Quantification of the samples was performed using an external standard for calibration. LOD and LOQ were 5 and 17 pg/mL, respectively. Deuterated SUL-138 (SUL138-d5) was used as internal standard.

### DIA data analysis

DIA data were searched against a spectral library of P2 biochemical subfractions from mouse hippocampus [74] using Spectronaut 13.7 [75] with default settings. The resulting abundance values and qualitative scores for each peptide in the spectral library were exported for further downstream analysis. MS-DAP 0.2.5 (https://github.com/ftwkoopmans/msdap) was used for the interpretation of data quality and differential expression analysis (DEA). While importing the Spectronaut data report, fragment group MS2 total peak areas without Spectronaut normalization were selected to represent peptide intensity values and both proteins from the MaxQuant contaminant database, and iRT peptides were removed from the dataset. Samples with demonstrable chromatographic aberrations, leading to substantially increased within-group coefficient of variation estimates, were highlighted in quality control figures and excluded from differential testing.

In each statistical contrast, peptides observed in both sample groups with Spectronaut confidence score ≤ 0.01 in at least 3 samples (biological replicates) were selected. Normalization was then applied to this data subset and finally MS-EmpiRe [76] was used for differential testing. All data visualizations and MS-DAP parameters are included in the MS-DAP report (Supplementary dataset 13). All raw protein data is provided in Supplementary Dataset 1.

### Immuno-histochemistry

Mouse brain was post-fixated by immersion in 4% paraformaldehyde (PFA) for 24 h with consequent incubation in 30% sucrose at 4 °C overnight. Coronal cross-sections were cut on a cryostat (− 20 °C, Leica CM1850) at 20 μM thickness and stored in PBS 1 × + 0.1% NaAz at 4 °C. Sections were cut at comparable bregma levels between groups and hippocampal area was similar between all sections (Figure S10). Sections were transferred to wells filled with 1x PBS for free-floating staining and washed 3x 10 min on a horizontal shaker at room temperature (RT). Followed by a blocking step of 1h at RT in blocking solution consisting of the following: 1× PBS, 5% normal goat serum, 2.5% bovine serum albumin, and 0.2% TRITON X-100 (Thermo Scientific). Samples were then incubated with primary anti-β-amyloid 1-16 antibody (BioLegend, mouse, 1:250), anti-glial fibrillary acidic protein (GFAP) (DAKO, rabbit, 1:1000), and anti-Iba1 (SySy, guinea pig, 1:400) in blocking solution, gently shaking at 4 °C overnight. The next day, tissue was washed 4x 10 min with 1× PBS. Alexa 488 goat anti-mouse lgG1, Alexa 568 goat anti-guinea pig lgG1, and Alexa 588 goat anti-rabbit lgG1 (Thermo Scientific, 1:400) were used for secondary antibody incubation during 1h at RT, covered to protect from fluorescent signal loss. Subsequently, tissue was washed 4x 10 min in 1× PBS and mounted on glass slides using Vectashield mounting medium with DAPI (Vector) and sealed with nail polish. Finished slides were immediately imaged.

### Microscopy and image analyses

Beta-amyloid plaques in the mouse brain were visualized using high-content fluorescence spectroscopy on a TissueFAXS system (TissueGnostics) paired with a Zeiss AxioObserver Z1. All images were acquired using a Zeiss – EC Plan Neonfluar 10 × (0.3 NA) objective with a CMOS-color PL-B623 Pixelink camera (3.1 Megapixels). Image acquisition was automatized after manual entry of the regions of interest on the sample in the TissueFAXS acquisition software, which stitched together individual acquisitions to create a representative image of the whole cross-section. Imaging was conducted at the UMCG Imaging and Microscopy Center (UMIC), which is sponsored by NWO-grants 40-00506-98-9021(TissueFaxs) and 175-010-2009-023 (Zeiss 2p). The images were inverted in Fiji [77] and plaque numbers and size were determined for the two slices per animal by setting an intensity threshold (the same for all images) and measuring above threshold intensity spots in the hippocampus. Per animal, the number and size of the plaques per hippocampi were averaged over the two slices and the four hippocampi.

### Statistics

GraphPad Prism 8.02 (for Windows, GraphPad Software, La Jolla, CA) was used for statistical analyses. For all statistical tests, a *p* or *q* (FDR correction in proteomics data) ≤ 0.05 was considered significant. Error bars show the standard error of the mean (SEM). The number of animals used for statistical analysis are indicated in all graphs by showing all data points. For pairwise comparisons, the Students *t*-test was used. For comparisons of three or more groups an ANOVA was used with a post hoc Fisher’s LSD test.

## Supplementary Information

**Additional file 1: Table S1.** SUL-138 plasma titers after 1 week of treatment.

**Additional file 2: Figure S1.** no significant difference in LTP between APP/PS1 VEH and WT VEH after 3x 100Hz stimulation. (A) Representative pre- (black) and post-tetanus (orange/purple) fEPSP traces for control WT and APP/PS1 mice. (B) LTP was measured as the fEPSP slope as percentage of baseline for control WT (orange) and APP/PS1 (purple) mice (n.s.: 2-way ANOVA, *p* > 0.05). (E) LTP maintenance after tetanus (3x 100Hz) was similar in vehicle treated WT (118.10 ± 4.17 and 110.90 ± 2.24) and APP/PS1 (117.60 ± 6.10 and 110.90 ± 3.99) mice (WT VEH *n* = 10 APP VEH *n* = 8; Student’s *t*-test, *p* ≥ 0.05).

**Additional file 3: Figure S2.** No differences in locomotor activity between SUL-138 and vehicle treated wildtype and APP/PS1 mice. APP/PS1 and wildtype mice did not differ in locomotor activity during the fear conditioning training session, as measured by (A) distance moved during training (cm; one-way ANOVA, n.s. *p* > 0.05) and (B) velocity of movement (cm/s; one-way ANOVA, n.s. *p* > 0.05). An expected increase in locomotor activity was observed, in APP/PS1 mice, showing significantly higher distance moved and velocity compared to WT mice (one-way ANOVA, *p ≤ 0.05).

**Additional file 4: Figure S3**. SUL-138 decreases amyloid plaque load. Representative images of APP/PS1 mice treated with vehicle (A) or SUL-138 (B) shows less amyloid plaques in the hippocampal area in SUL-138 treated APP/PS1 mice (amyloid-beta staining in black, amyloid plaques in the hippocampus circled in red).

**Additional file 5: Figure S4.** GFAP and Iba1 staining in vehicle- and SUL-138 treated APP/PS1 and wildtype mice. (A) Sections of vehicle- and SUL-138-treated APP/PS1 and wildtypes (WT) mice were stained with DAPI (nuclei), anti-GFAP (astrocytes) and anti-Iba1 (microglia) (*n* = 5/group). (B/C) Both GFAP and Iba1 showed higher expression (% of total area/image) in APP/PS1 mice than in WT mice (*p* = 0.0295 and *p* = 0.0748; one-way ANOVA, post hoc Fisher’s LSD). Treatment with SUL-138 did not alter GFAP or Iba1 expression in either WT or APP/PS1 mice (*p* > 0.05; one-way ANOVA, post hoc Fisher’s LSD). Scale bar: 100μm.

**Additional file 6: Figure S5.** Biological process enrichment of significantly regulated proteins. Top 10 enriched BP GO terms for upregulated (top panel) and downregulated (lower panel) proteins for each of the 3 contrasts: (A) APP VEH vs. WT VEH, (B) APP SUL vs. APP VEH and (C) WT SUL vs. WT VEH. Size of the dots represents the number of proteins annotated to the GO term and the fraction of significant proteins is the number of significant proteins divided by the total number of proteins belonging to that term.

**Additional file 7: Figure S6.** AD associated protein regulation. Volcano plots showing (dys)regulation of proteins in the three relevant comparisons: APP VEH vs. WT VEH (A), APP SUL vs, APP VEH (B) and WT SUL vs. WT VEH (C). Highlighted in pink are AD associated proteins (GWAS and UniProt).

**Additional file 8: Figure S7.** Cellular component and biological process enrichment of overlap APP VEH vs. WT VEH and APP SUL vs. APP VEH. Top 10 enriched (A) CC (top panel) and (B) BP (lower panel) GO terms for proteins that were dysregulated in APP/PS1 (APP VEH vs. WT VEH) and are altered by SUL-138 (APP SUL vs. APP VEH). Size of the dots represents the number of proteins annotated to the GO term and the fraction of significant proteins is the number of significant proteins divided by the total number of proteins belonging to that term.

**Additional file 9: Figure S8.** SynGO gene counts of synaptic protein regulation by SUL-138 in APP/PS1 and wildtype mice. (A) Sunburst plots of gene counts for significantly downregulated proteins in APP VEH vs. WT VEH using SynGO showing extensive dysregulation throughout the synapse (B) Sunburst plots of gene counts of significantly downregulated proteins in APP SUL vs. APP VEH shows pre- and postsynaptic protein regulation (C) Sunburst plots of CC enrichment for significantly down- and upregulated proteins in WT SUL vs. WT VEH shows pre- and postsynaptic protein regulation.

**Additional file 10: Figure S9.** mitochondrial protein regulation in APP SUL vs. APP VEH. Schematic representations of the three main metabolic inputs towards the TCA cycle and oxidative phosphorylation (OXPHOS; ETC): glycolysis, FAD and FAO, and amino acid metabolism. In red and blue significantly up- and downregulated proteins (FDR, *q* ≤ 0.05) are indicated.

**Additional file 11: Figure S10.** Hippocampal area is similar between vehicle and SUL-138 treated APP/PS1. Hippocampal area of all hippocampi analyzed for plaques was determined using Fiji [77]. The areas did not differ between vehicle treated (APP VEH; clear purple bar) and SUL-138 treated (APP SUL; filled purple bar) (n.s. *p* > 0.05; student’s *t*-test).

**Additional file 12:** **Supplementary dataset 1.** differential expression analysis (DEA).

**Additional file 13:** **Supplementary dataset 2.** GO analysis of protein dysregulation in the hippocampus due to disease (APP/PS1 mice compared to wildtype controls - APP VEH vs. WT VEH); significantly down regulated group.

**Additional file 14:** **Supplementary dataset 3.** GO analysis of protein dysregulation in the hippocampus due to disease (APP/PS1 mice compared to wildtype controls - APP VEH vs. WT VEH); significantly up regulated group.

**Additional file 15:** **Supplementary dataset 4.** GO analysis of hippocampal protein regulation as a result of 3 months of SUL-138 treatment in APP/PS1 mice (APP SUL vs. APP VEH); significantly down regulated group.

**Additional file 16:** **Supplementary dataset 5.** GO analysis of hippocampal protein regulation as a result of 3 months of SUL-138 treatment in APP/PS1 mice (APP SUL vs. APP VEH); significantly up regulated group.

**Additional file 17:** **Supplementary dataset 6.** GO analysis of SUL-138-regulated proteins overlapping with proteins affected in APP/PS1 mice.

**Additional file 18:** **Supplementary dataset 7.** GO analysis of SUL-138-regulated proteins overlap with proteins affected in APP/PS1 mice; significantly down regulated group.

**Additional file 19:** **Supplementary dataset 8.** GO analysis of SUL-138-regulated proteins overlapping with proteins affected in APP/PS1 mice; significantly up regulated group.

**Additional file 20:** **Supplementary dataset 9.** SynGO annotation of significantly regulated proteins in the WT VEH vs. APP VEH comparison.

**Additional file 21:** **Supplementary dataset 10.** SynGO annotation of significantly regulated proteins in the APP VEH vs. APP SUL comparison.

**Additional file 22:** **Supplementary dataset 11.** SynGO annotation of significantly regulated proteins in the WT VEH vs. WT SUL comparison.

**Additional file 23:** **Supplementary dataset 12.** Mitocarta and mitoXplorer data analysis.

**Additional file 24:** **Supplementary dataset 13.** MS-DAP report.

## Data Availability

All data is available, either as supplemental data set of this manuscript, or by request to the corresponding authors.

## Abbreviations

AD: Alzheimer’s disease
ROS: Reactive oxygen species
LTP: Long-term potentiation
NC: Novel Context
FC: Fear conditioning
VEH: Vehicle
SUL: SUL-138
WT: Wildtype
APP: APP/PS1
FAD: Fatty acid degradation
FAO: Fatty acid oxidation
OXPHOS: Oxidative phosphorylation system
ETC: Electron transport chain
