# Supplementary figures and images for: The hibernation-derived compound SUL-138 shifts the mitochondrial proteome towards fatty acid metabolism and prevents cognitive decline and amyloid plaque formation in an Alzheimer’s disease mouse model
Source: Alzheimers Res Ther. 2022 Dec 9;14:183. doi: 10.1186/s13195-022-01127-z (PMC9733344; doi:10.1186/s13195-022-01127-z)

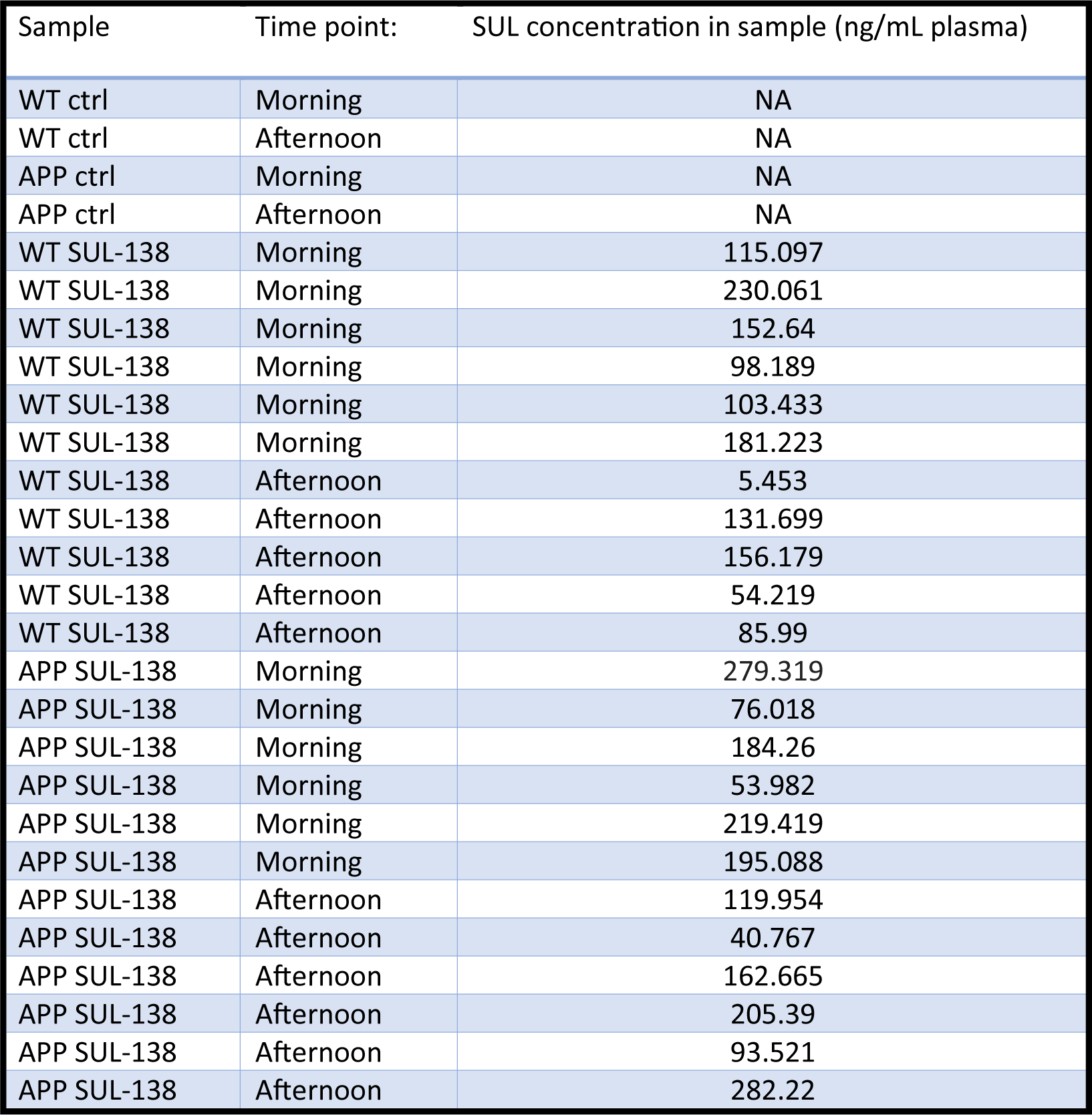

Supplement: Supplementary file 1 — Additional file 1: Table S1. SUL-138 plasma titers after 1 week of treatment. [file 13195_2022_1127_MOESM1_ESM.tif]

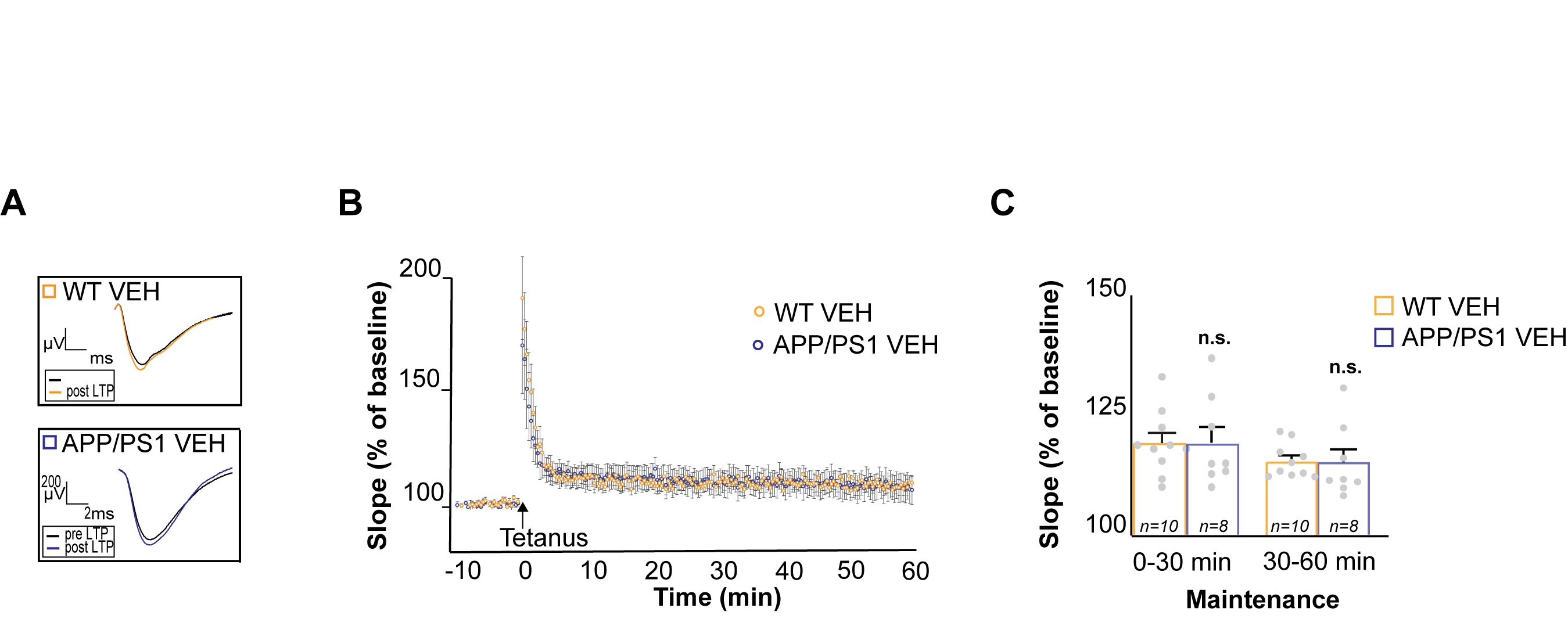

Supplement: Supplementary file 2 — Additional file 2: Figure S1. no significant difference in LTP between APP/PS1 VEH and WT VEH after 3x 100Hz stimulation. (A) Representative pre- (black) and post-tetanus (orange/purple) fEPSP traces for control WT and APP/PS1 mice. (B) LTP was measured as the fEPSP slope as percentage of baseline for control WT (orange) and APP/PS1 (purple) mice (n.s.: 2-way ANOVA, p > 0.05). (E) LTP maintenance after tetanus (3x 100Hz) was similar in vehicle treated WT (118.10 ± 4.17 and 110.90 ± 2.24) and APP/PS1 (117.60 ± 6.10 and 110.90 ± 3.99) mice (WT VEH n = 10 APP VEH n = 8; Student’s t-test, p ≥ 0.05). [file 13195_2022_1127_MOESM2_ESM.tif]

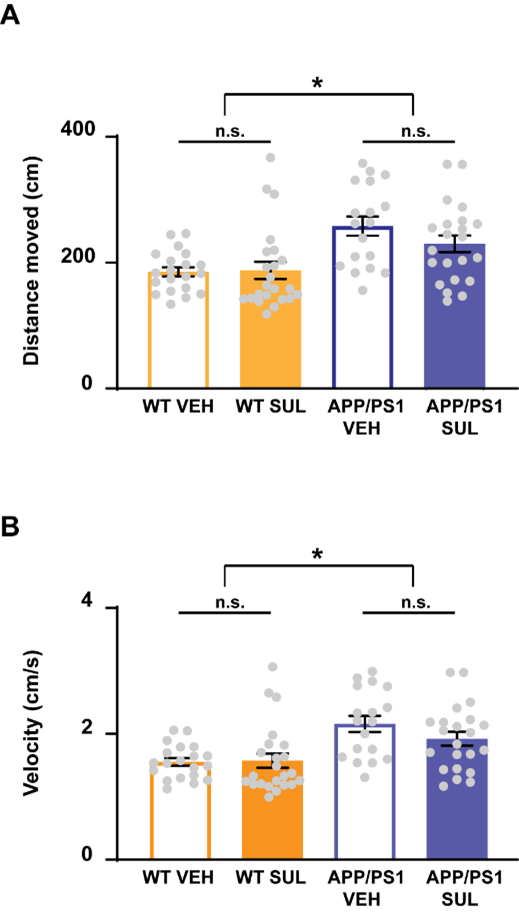

Supplement: Supplementary file 3 — Additional file 3: Figure S2. No differences in locomotor activity between SUL-138 and vehicle treated wildtype and APP/PS1 mice. APP/PS1 and wildtype mice did not differ in locomotor activity during the fear conditioning training session, as measured by (A) distance moved during training (cm; one-way ANOVA, n.s. p > 0.05) and (B) velocity of movement (cm/s; one-way ANOVA, n.s. p > 0.05). An expected increase in locomotor activity was observed, in APP/PS1 mice, showing significantly higher distance moved and velocity compared to WT mice (one-way ANOVA, *p ≤ 0.05). [file 13195_2022_1127_MOESM3_ESM.tif]

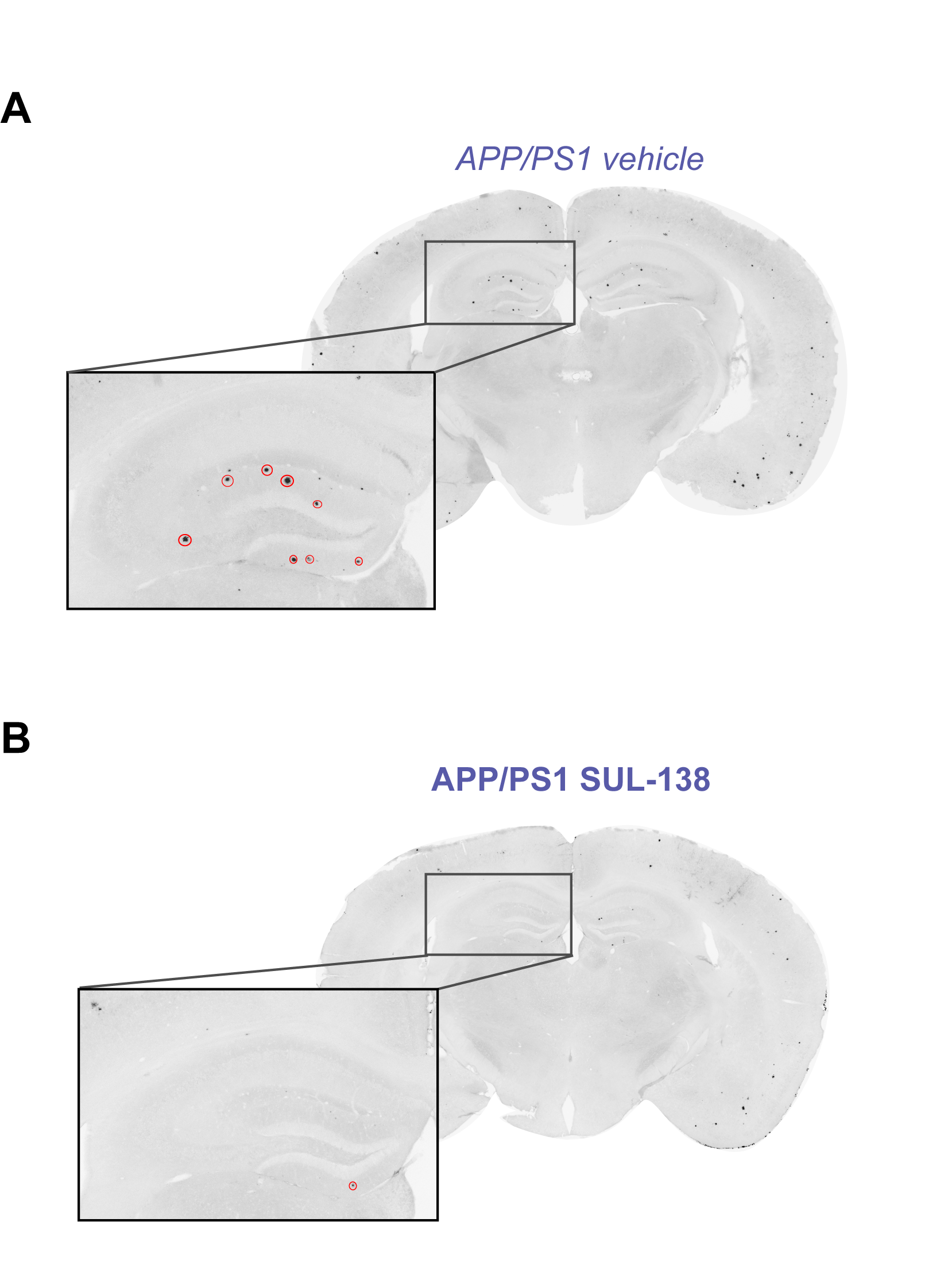

Supplement: Supplementary file 4 — Additional file 4: Figure S3. SUL-138 decreases amyloid plaque load. Representative images of APP/PS1 mice treated with vehicle (A) or SUL-138 (B) shows less amyloid plaques in the hippocampal area in SUL-138 treated APP/PS1 mice (amyloid-beta staining in black, amyloid plaques in the hippocampus circled in red). [file 13195_2022_1127_MOESM4_ESM.tif]

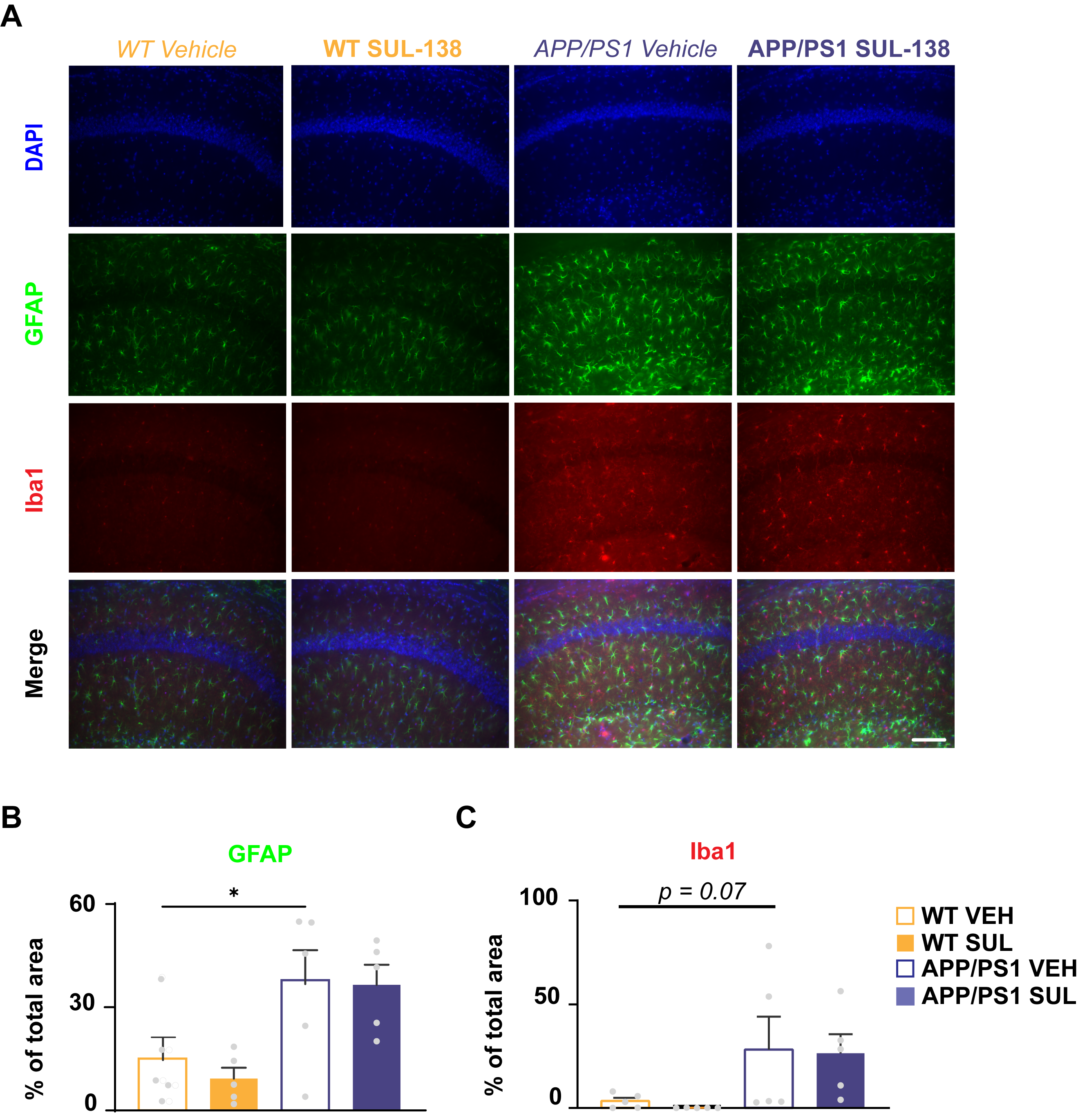

Supplement: Supplementary file 5 — Additional file 5: Figure S4. GFAP and Iba1 staining in vehicle- and SUL-138 treated APP/PS1 and wildtype mice. (A) Sections of vehicle- and SUL-138-treated APP/PS1 and wildtypes (WT) mice were stained with DAPI (nuclei), anti-GFAP (astrocytes) and anti-Iba1 (microglia) (n = 5/group). (B/C) Both GFAP and Iba1 showed higher expression (% of total area/image) in APP/PS1 mice than in WT mice (p = 0.0295 and p = 0.0748; one-way ANOVA, post hoc Fisher’s LSD). Treatment with SUL-138 did not alter GFAP or Iba1 expression in either WT or APP/PS1 mice (p > 0.05; one-way ANOVA, post hoc Fisher’s LSD). Scale bar: 100μm. [file 13195_2022_1127_MOESM5_ESM.tif]

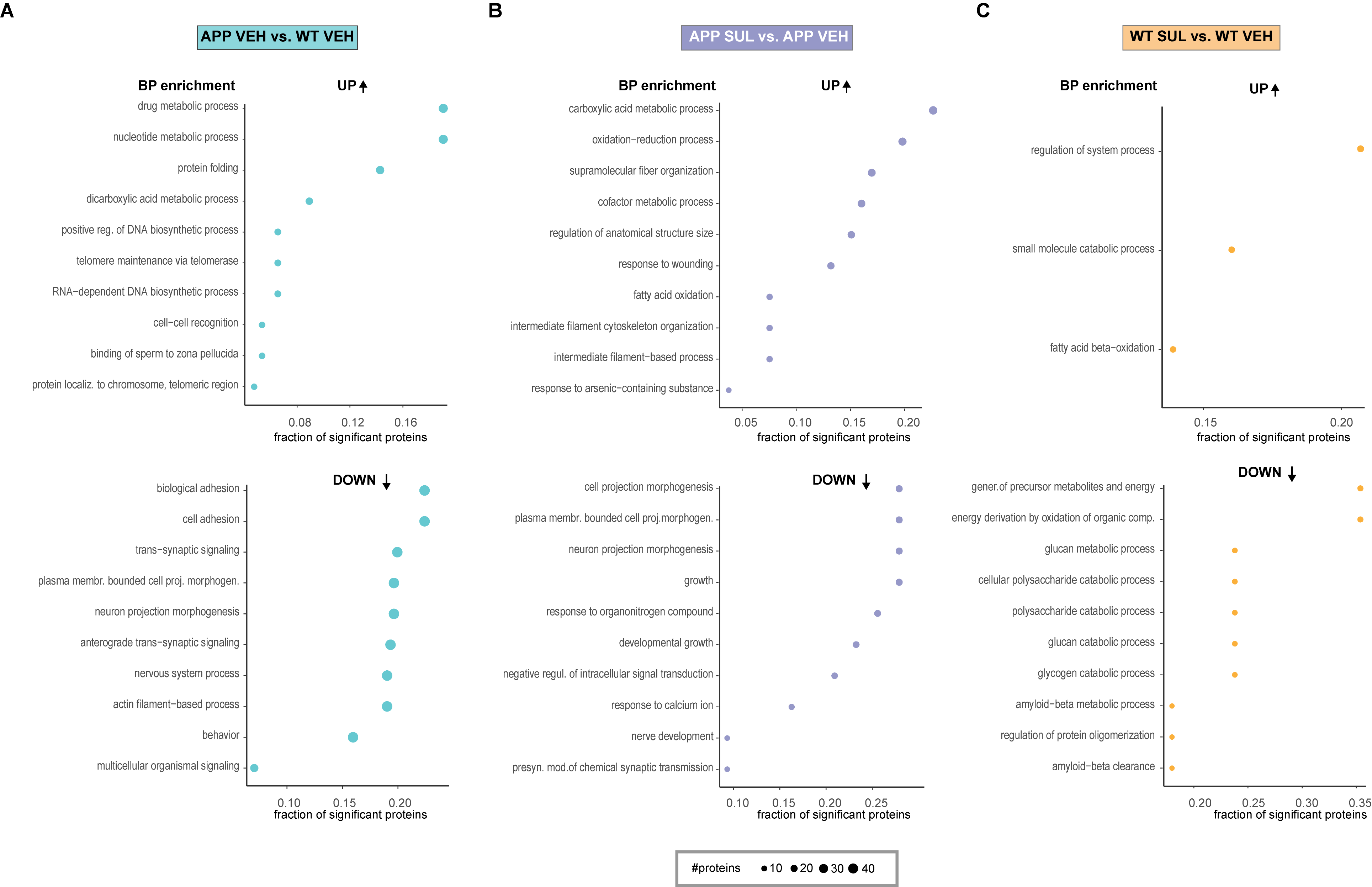

Supplement: Supplementary file 6 — Additional file 6: Figure S5. Biological process enrichment of significantly regulated proteins. Top 10 enriched BP GO terms for upregulated (top panel) and downregulated (lower panel) proteins for each of the 3 contrasts: (A) APP VEH vs. WT VEH, (B) APP SUL vs. APP VEH and (C) WT SUL vs. WT VEH. Size of the dots represents the number of proteins annotated to the GO term and the fraction of significant proteins is the number of significant proteins divided by the total number of proteins belonging to that term. [file 13195_2022_1127_MOESM6_ESM.tif]

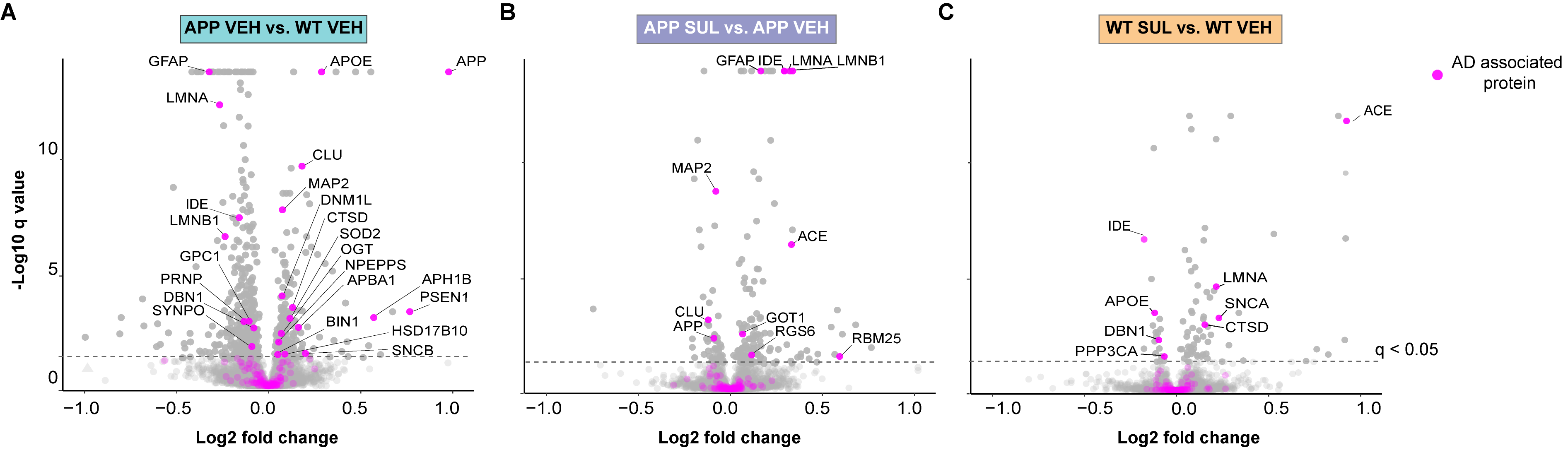

Supplement: Supplementary file 7 — Additional file 7: Figure S6. AD associated protein regulation. Volcano plots showing (dys)regulation of proteins in the three relevant comparisons: APP VEH vs. WT VEH (A), APP SUL vs, APP VEH (B) and WT SUL vs. WT VEH (C). Highlighted in pink are AD associated proteins (GWAS and UniProt). [file 13195_2022_1127_MOESM7_ESM.tif]

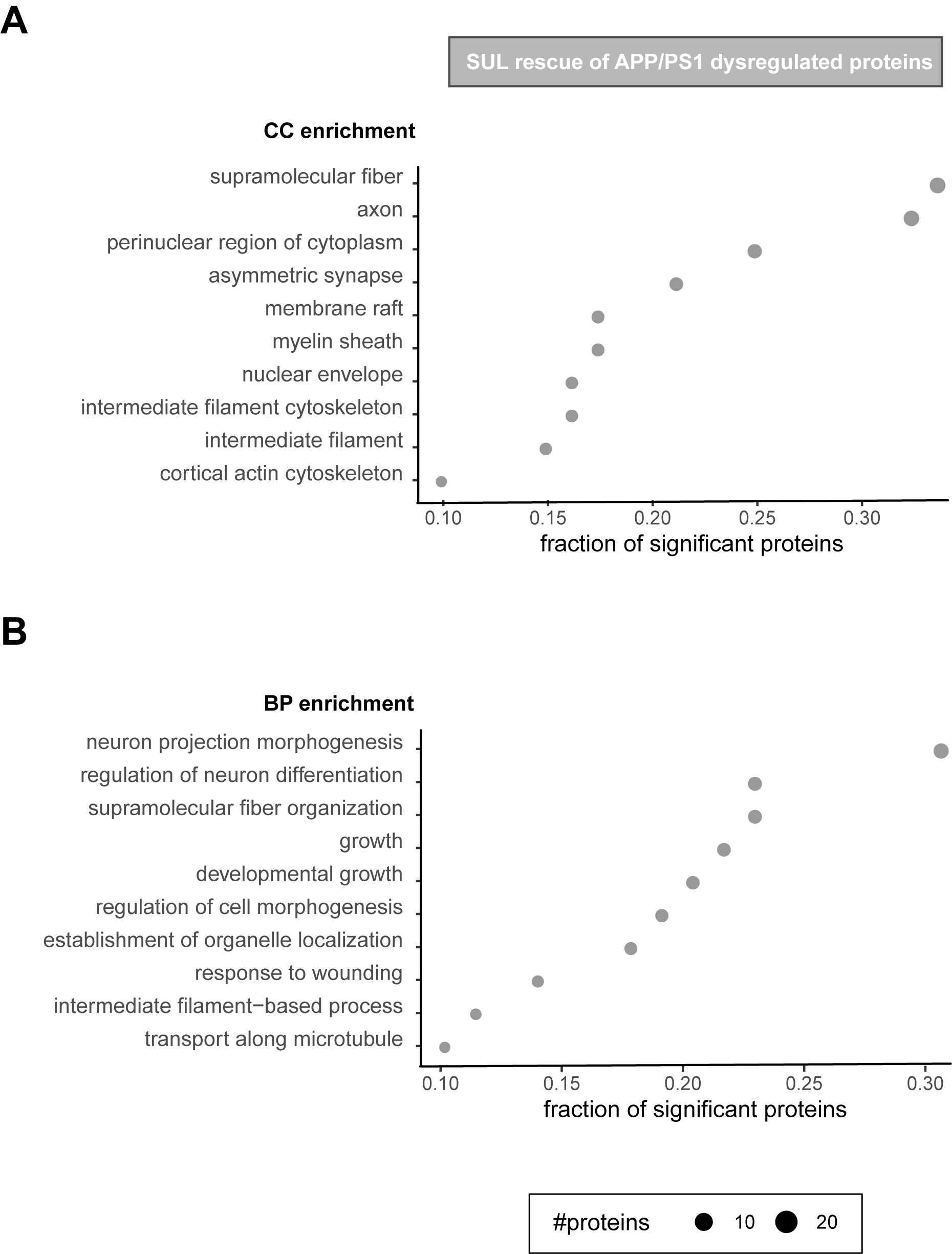

Supplement: Supplementary file 8 — Additional file 8: Figure S7. Cellular component and biological process enrichment of overlap APP VEH vs. WT VEH and APP SUL vs. APP VEH. Top 10 enriched (A) CC (top panel) and (B) BP (lower panel) GO terms for proteins that were dysregulated in APP/PS1 (APP VEH vs. WT VEH) and are altered by SUL-138 (APP SUL vs. APP VEH). Size of the dots represents the number of proteins annotated to the GO term and the fraction of significant proteins is the number of significant proteins divided by the total number of proteins belonging to that term. [file 13195_2022_1127_MOESM8_ESM.tif]

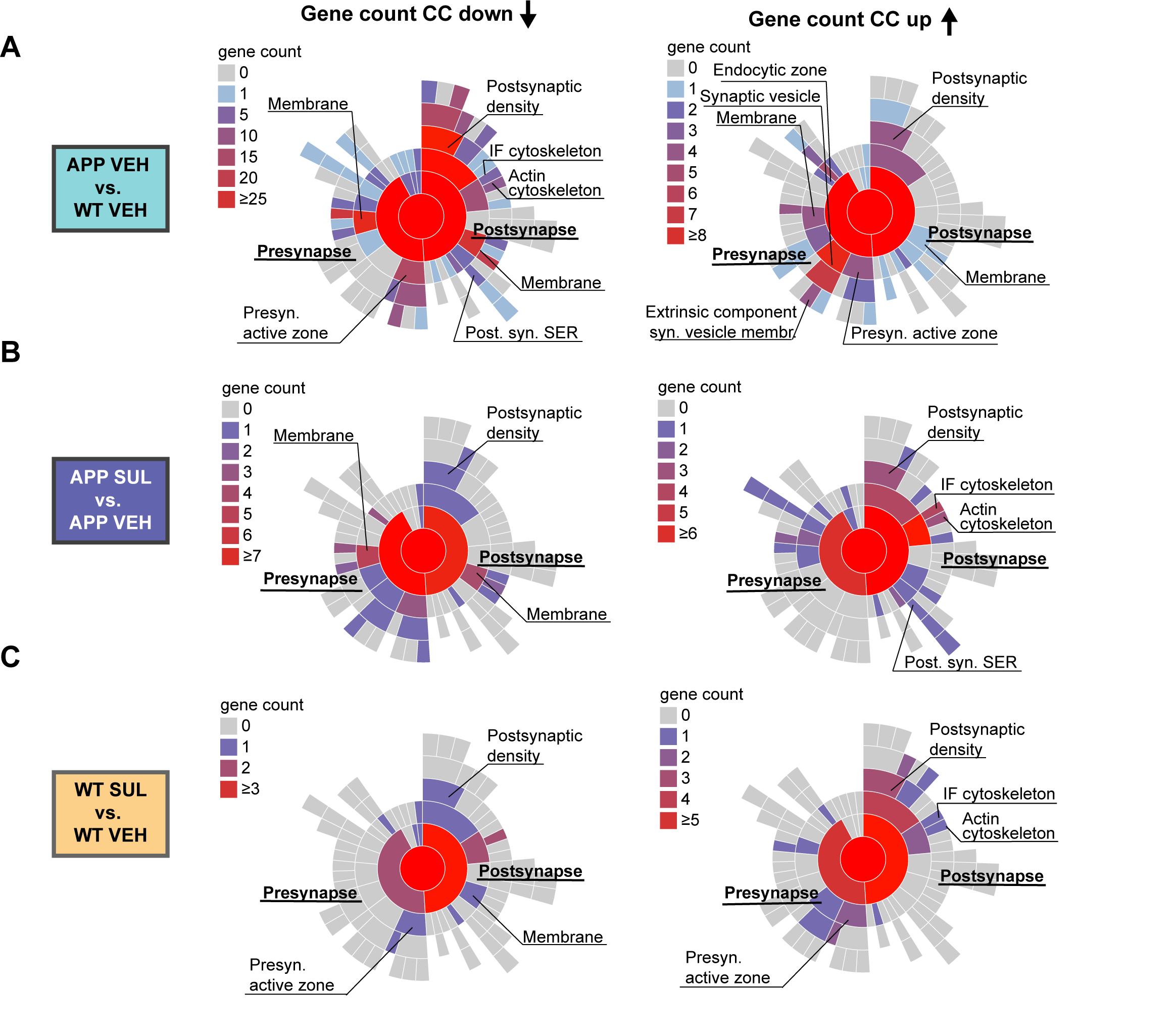

Supplement: Supplementary file 9 — Additional file 9: Figure S8. SynGO gene counts of synaptic protein regulation by SUL-138 in APP/PS1 and wildtype mice. (A) Sunburst plots of gene counts for significantly downregulated proteins in APP VEH vs. WT VEH using SynGO showing extensive dysregulation throughout the synapse (B) Sunburst plots of gene counts of significantly downregulated proteins in APP SUL vs. APP VEH shows pre- and postsynaptic protein regulation (C) Sunburst plots of CC enrichment for significantly down- and upregulated proteins in WT SUL vs. WT VEH shows pre- and postsynaptic protein regulation. [file 13195_2022_1127_MOESM9_ESM.tif]

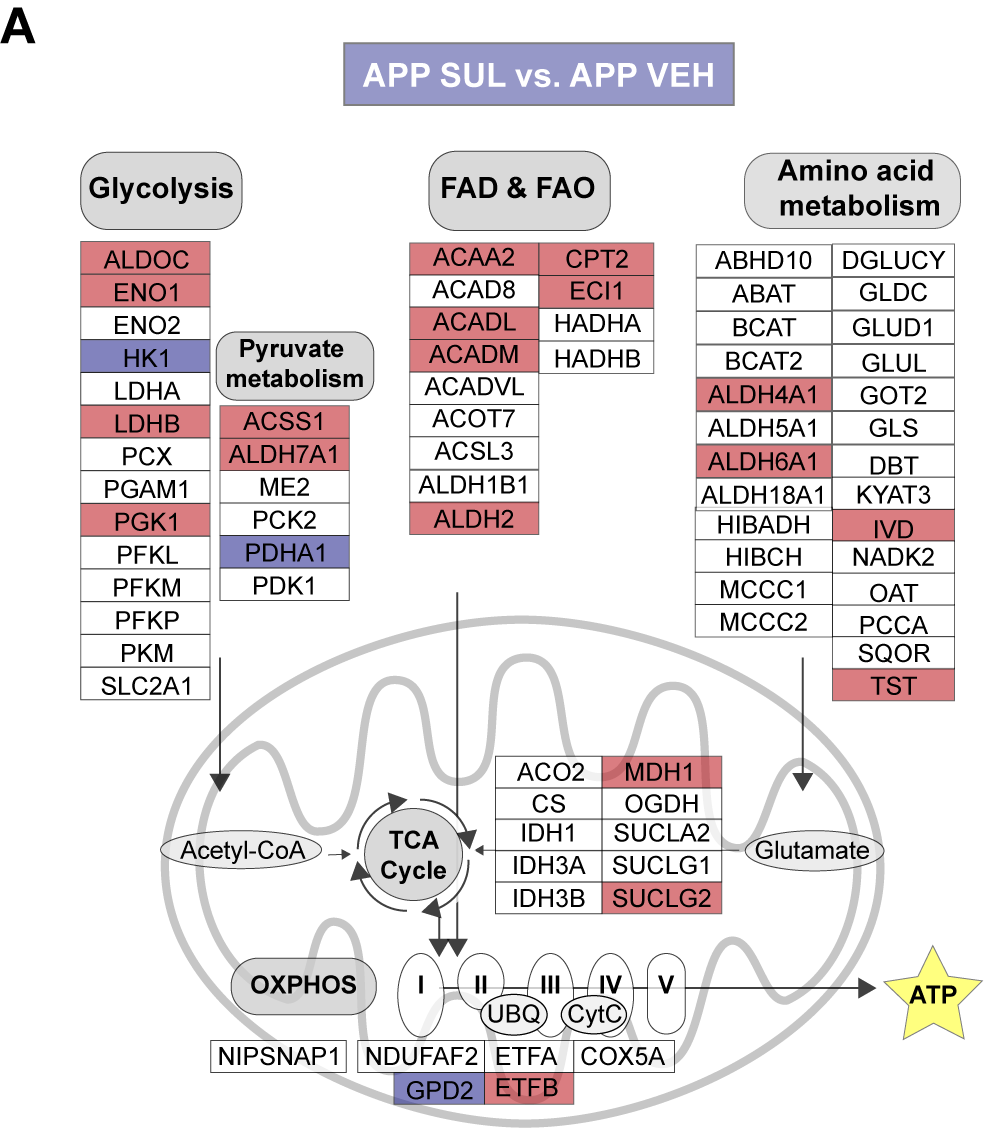

Supplement: Supplementary file 10 — Additional file 10: Figure S9. mitochondrial protein regulation in APP SUL vs. APP VEH. Schematic representations of the three main metabolic inputs towards the TCA cycle and oxidative phosphorylation (OXPHOS; ETC): glycolysis, FAD and FAO, and amino acid metabolism. In red and blue significantly up- and downregulated proteins (FDR, q ≤ 0.05) are indicated. [file 13195_2022_1127_MOESM10_ESM.tif]

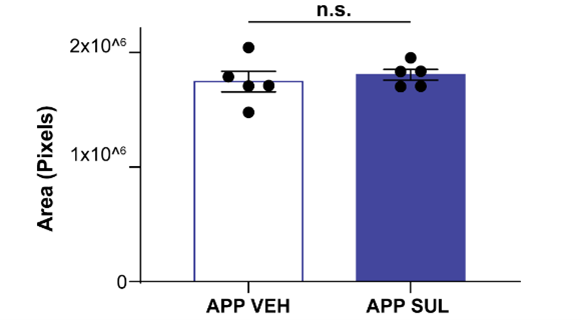

Supplement: Supplementary file 11 — Additional file 11: Figure S10. Hippocampal area is similar between vehicle and SUL-138 treated APP/PS1. Hippocampal area of all hippocampi analyzed for plaques was determined using Fiji [77]. The areas did not differ between vehicle treated (APP VEH; clear purple bar) and SUL-138 treated (APP SUL; filled purple bar) (n.s. p > 0.05; student’s t-test). [file 13195_2022_1127_MOESM11_ESM.tif]
