# Supplementary material for: The hibernation-derived compound SUL-138 shifts the mitochondrial proteome towards fatty acid metabolism and prevents cognitive decline and amyloid plaque formation in an Alzheimer’s disease mouse model
Source: Alzheimers Res Ther. 2022 Dec 9;14:183. doi: 10.1186/s13195-022-01127-z (PMC9733344; doi:10.1186/s13195-022-01127-z)
Supplement: Supplementary file 24 — Additional file 24: Supplementary dataset 13. MS-DAP report. [file 13195_2022_1127_MOESM24_ESM.pdf]

MS-DAP: Mass Spectrometry Downstream Analysis Pipeline

version: beta 0.1.7.2 https://github.com/ftwkoopmans/msdap/

Contents

1 Quality control **2**

1.1 DIA confidence score distributions 2

1.2 number of peptides and proteins 5

1.3 data completeness 10

1.4 abundance distributions 12

1.5 retention time 15

1.6 variation among replicates 28

1.7 PCA 36

2 Differential abundance analysis **42**

2.1 wt_ctrl vs wt_sul 42

2.2 app_ctrl vs app_sul 48

2.3 wt_ctrl vs app_ctrl 54

2.4 wt_sul vs app_sul 60

3 Summary of statistical results **66**

4 log **67**

5 R command history **68**

6 R session info **69**

1 Quality control

The quality control figures in this section enable you to investigate reproducibility and global clustering of samples by visualizing:

- number of peptides/proteins detected in each sample
- dataset completeness
- local effects in HPLC peptide retention time per sample
- reproducibility of peptide quantification among replicates
- PCA of all samples to visualize clustering

The first set of quality control figures describes individual samples, thereafter group-level quality metrics are described and finally sample clustering is used to highlight structure in the entire dataset.

1.1 DIA confidence score distributions

DIA data was used as input for this analysis. Below histograms visualize both target (green) and decoy (grey) cscores in each sample, indicating how confident the input software was in the identification of peptides from the spectral library in the raw DIA data. Samples are ordered by the number of precursors quantified at q-value confidence threshold 0.01. At this threshold, the respective cscore and number of peptides is shown.

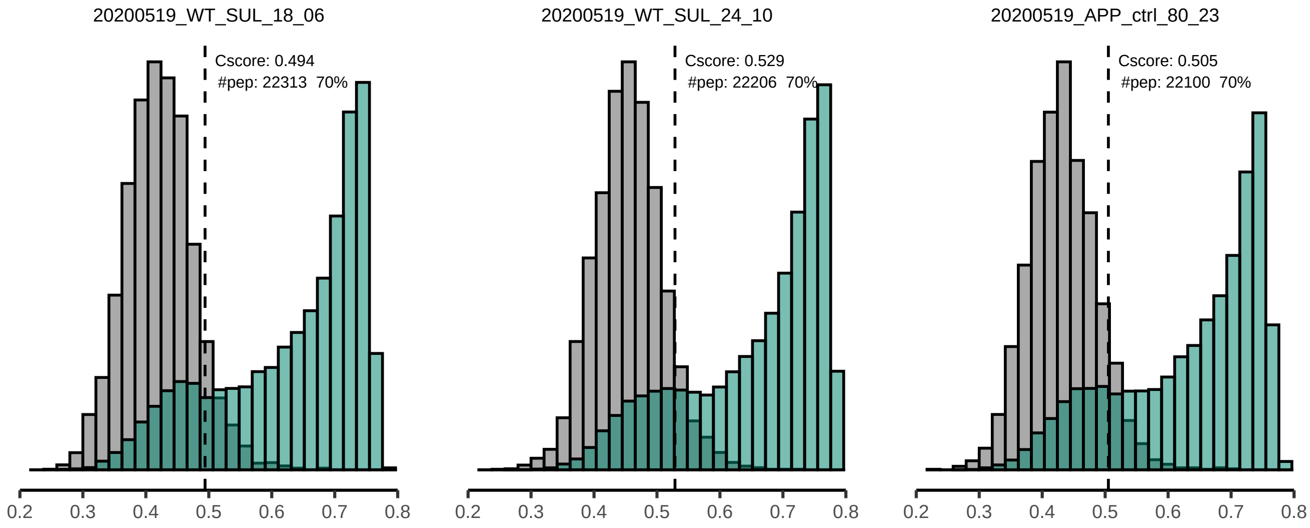

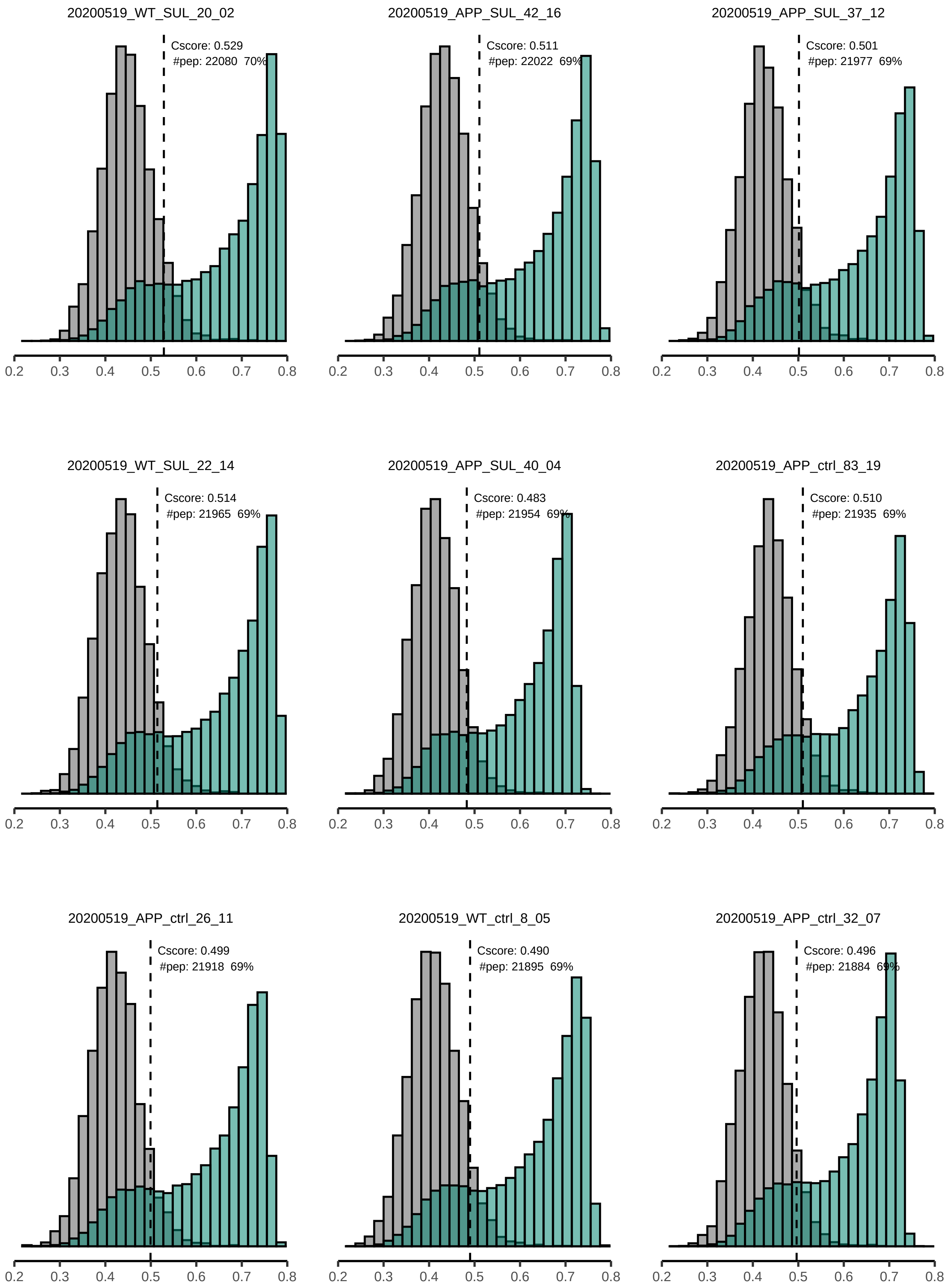
20200519_WT_SUL_20_02
Cscore: 0.529
#pep: 22080 70%
20200519_APP_SUL_42_16
Cscore: 0.511
#pep: 22022 69%
20200519_APP_SUL_37_12
Cscore: 0.501
#pep: 21977 69%
20200519_WT_SUL_22_14
Cscore: 0.514
#pep: 21965 69%
20200519_APP_SUL_40_04
Cscore: 0.483
#pep: 21954 69%
20200519_APP_ctrl_83_19
Cscore: 0.510
#pep: 21935 69%
20200519_APP_ctrl_26_11
Cscore: 0.499
#pep: 21918 69%
20200519_WT_ctrl_8_05
Cscore: 0.490
#pep: 21895 69%
20200519_APP_ctrl_32_07
Cscore: 0.496
#pep: 21884 69%
0.2
0.3
0.4
0.5
0.6
0.7
0.8

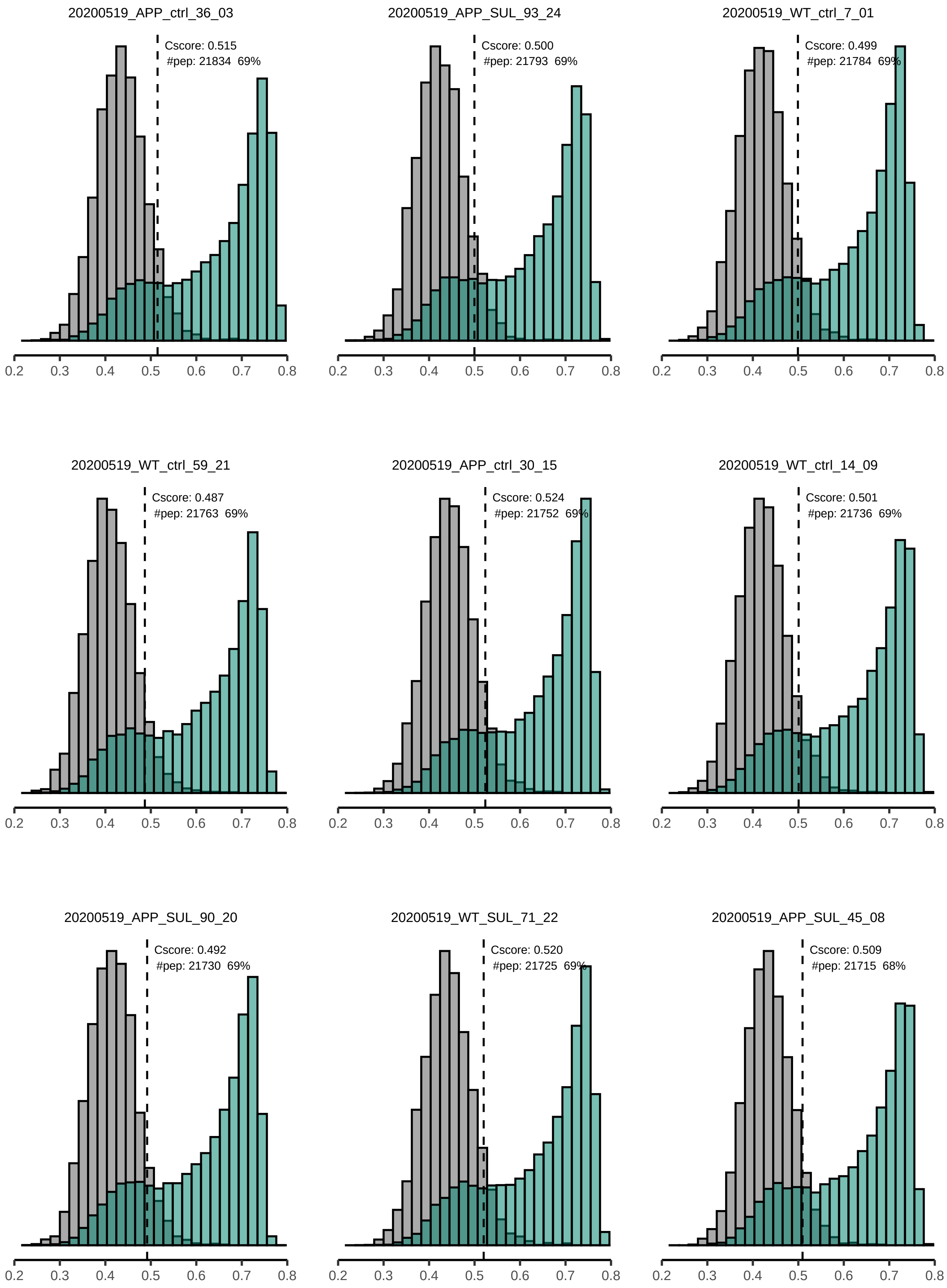
20200519_APP_ctrl_36_03
Cscore: 0.515
#pep: 21834 69%
20200519_APP_SUL_93_24
Cscore: 0.500
#pep: 21793 69%
20200519_WT_ctrl_7_01
Cscore: 0.499
#pep: 21784 69%
20200519_WT_ctrl_59_21
Cscore: 0.487
#pep: 21763 69%
20200519_APP_ctrl_30_15
Cscore: 0.524
#pep: 21752 69%
20200519_WT_ctrl_14_09
Cscore: 0.501
#pep: 21736 69%
20200519_APP_SUL_90_20
Cscore: 0.492
#pep: 21730 69%
20200519_WT_SUL_71_22
Cscore: 0.520
#pep: 21725 69%
20200519_APP_SUL_45_08
Cscore: 0.509
#pep: 21715 68%
0.2 0.3 0.4 0.5 0.6 0.7 0.8

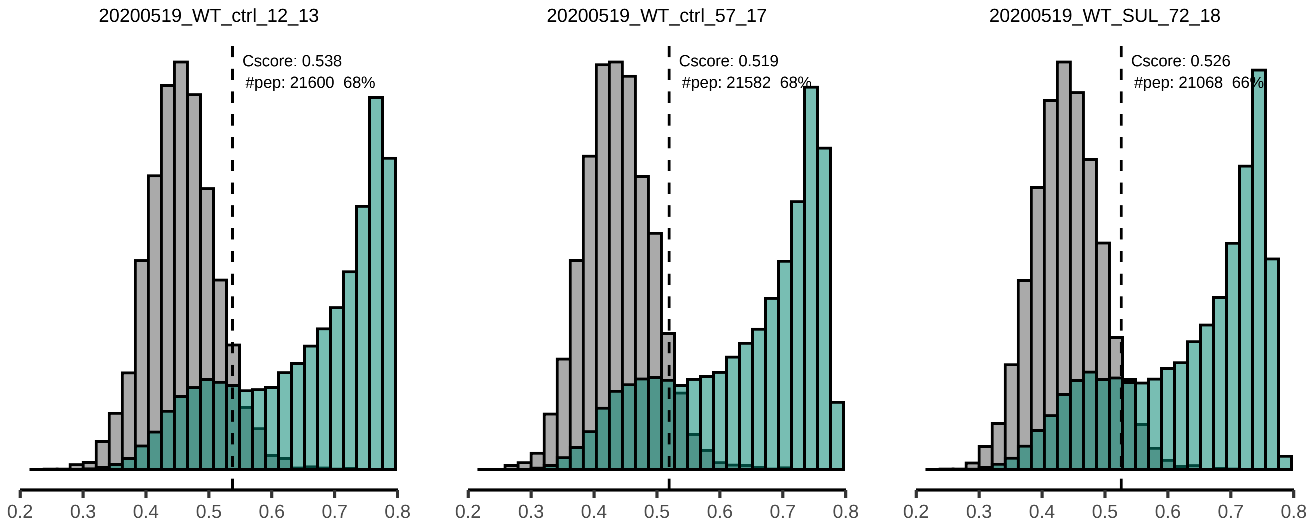

1.2 number of peptides and proteins

These plots show the number of (target) peptides that are 'detected' per sample. For DDA, 'detected' implies the peptide has a MS/MS identification. Peptides quantified through match-between-runs (MBR) are quantified but not detected/identified. In case of DDA, we also show the number of peptides quantified through MBR. For DIA, we refer to a peptide as 'detected' if the confidence score (for identification) is <= 0.01.

Samples in this plot are sorted by their experimental group, and then ordered and by their name within each group. This data is also available in the output table 'samples.xlsx'.

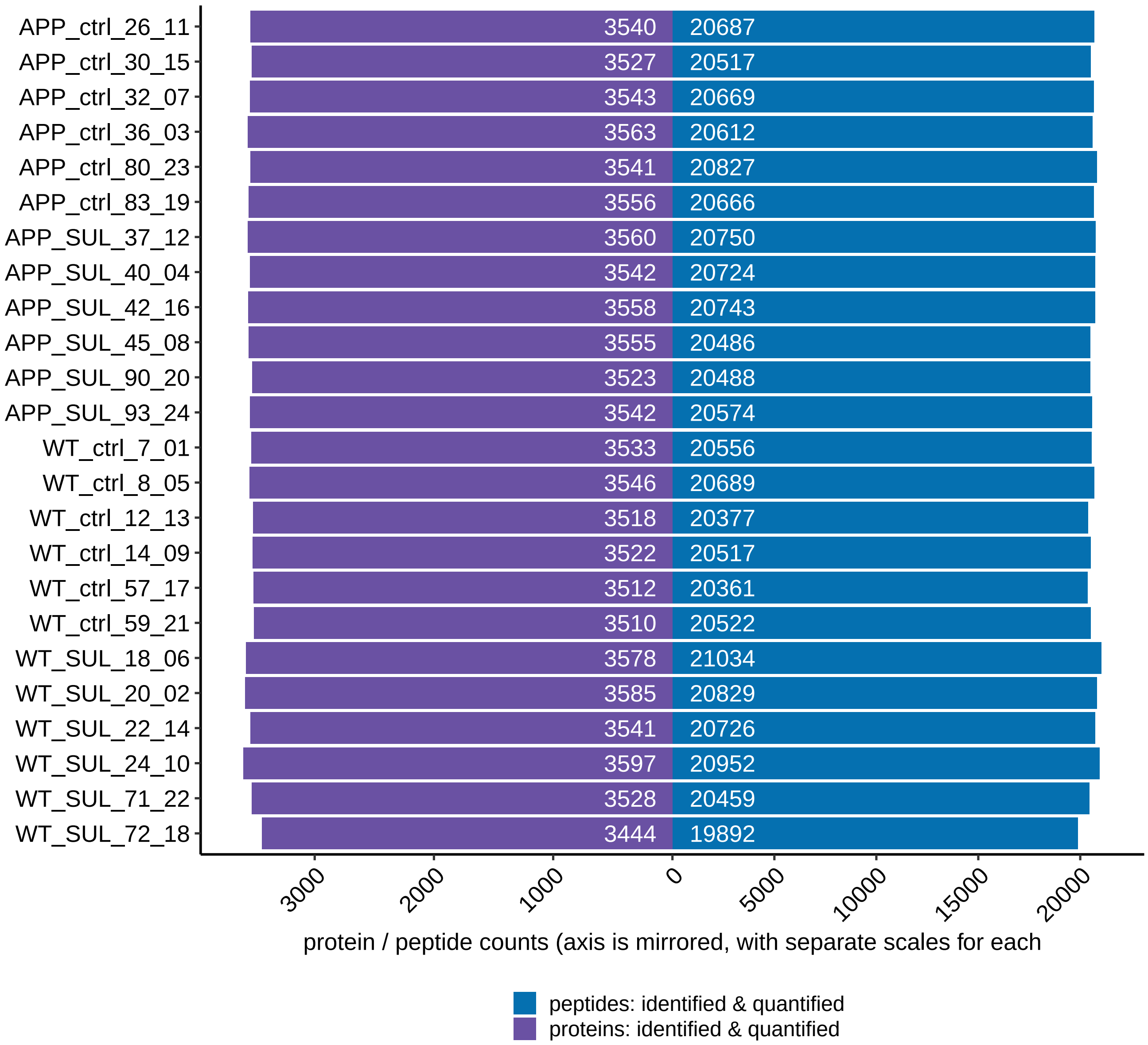

APP_ctrl_26_11 3540 20687
APP_ctrl_30_15 3527 20517
APP_ctrl_32_07 3543 20669
APP_ctrl_36_03 3563 20612
APP_ctrl_80_23 3541 20827
APP_ctrl_83_19 3556 20666
APP_SUL_37_12 3560 20750
APP_SUL_40_04 3542 20724
APP_SUL_42_16 3558 20743
APP_SUL_45_08 3555 20486
APP_SUL_90_20 3523 20488
APP_SUL_93_24 3542 20574
WT_ctrl_7_01 3533 20556
WT_ctrl_8_05 3546 20689
WT_ctrl_12_13 3518 20377
WT_ctrl_14_09 3522 20517
WT_ctrl_57_17 3512 20361
WT_ctrl_59_21 3510 20522
WT_SUL_18_06 3578 21034
WT_SUL_20_02 3585 20829
WT_SUL_22_14 3541 20726
WT_SUL_24_10 3597 20952
WT_SUL_71_22 3528 20459
WT_SUL_72_18 3444 19892
3000 2000 1000 0 5000 10000 15000 20000
protein / peptide counts (axis is mirrored, with separate scales for each
peptides: identified & quantified
proteins: identified & quantified

1.2.1 color-coding sample metadata

The number of detected peptides in a sample, as compared to other samples within a dataset, can be used as a measure for sample quality. Color-coding individual samples for metadata that you provided as input (e.g. experiment batch, sample handling order, gel lanes, etc.) allows visual inspection as to whether these relate to the rate of successful peptide detection.

The figure below aims to provide an overview of all sample metadata at a first glance. The y-axis shows all sample metadata. On each row all samples in the dataset are shown as a dot, each color-coded by the respective property shown on the y-axis (with minor vertical jitter for visual clarity). If there is a major effect of any experimental condition on the number of detected peptides, this is easily spotted as all outlier samples (extremes in number peptide detection, x-axis) will have the same color. The following figures expand this overview into a detailed figure for each sample property, those visualization are designed to further dive into each experimental condition and provide respective color legends.

Note that the visualization of sample metadata in this report depends on user-provided input; each column in the metadata input table (besides sample names) that contains more than 1 unique value is automatically used as a factor for color-coding all figures in this section. So the data used for color-coding is also available in the output table 'samples.xlsx'.

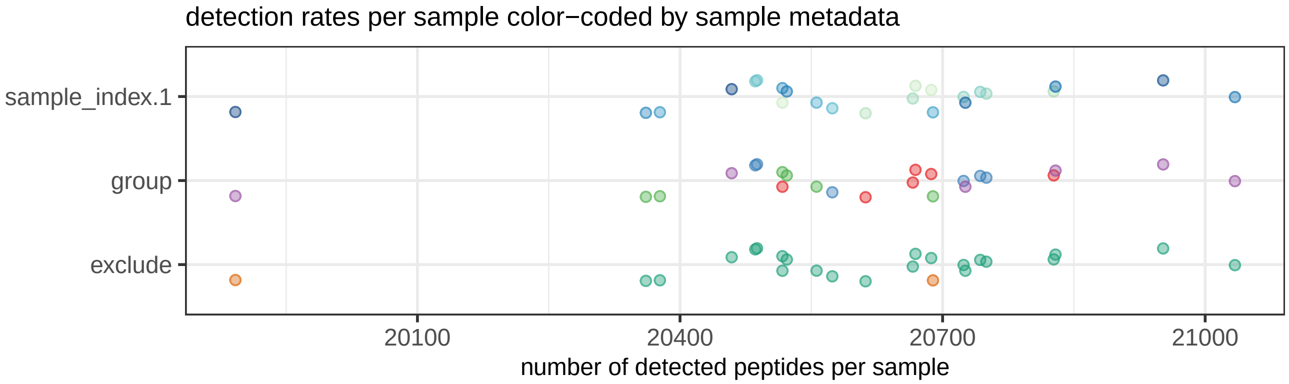

color-coding sample metadata, expanded

To further detail each sample property, each row in the above figure is now split into separate plots. Thus, a figure is generated for each experimental condition from the user-provided metadata (column in the samples table, it's name shown in the plot title).

The y-axis shows all unique variables, the x-axis the number of detected peptides and each dot is a sample. The vertical line indicates the median value for each row. Colors are consistent with the above plot.

For example: the first plot shows color-coding by the 'group' property, so each row represents a sample group. If samples in a particular group systematically yield fewer peptides than another group, a clear pattern will be visible.

Note that the *exclude* metadata is a user-provided indication of 'outlier samples' in the dataset that are excluded from downstream statistical analysis.

sample metadata used for color−coding: sample_index.1

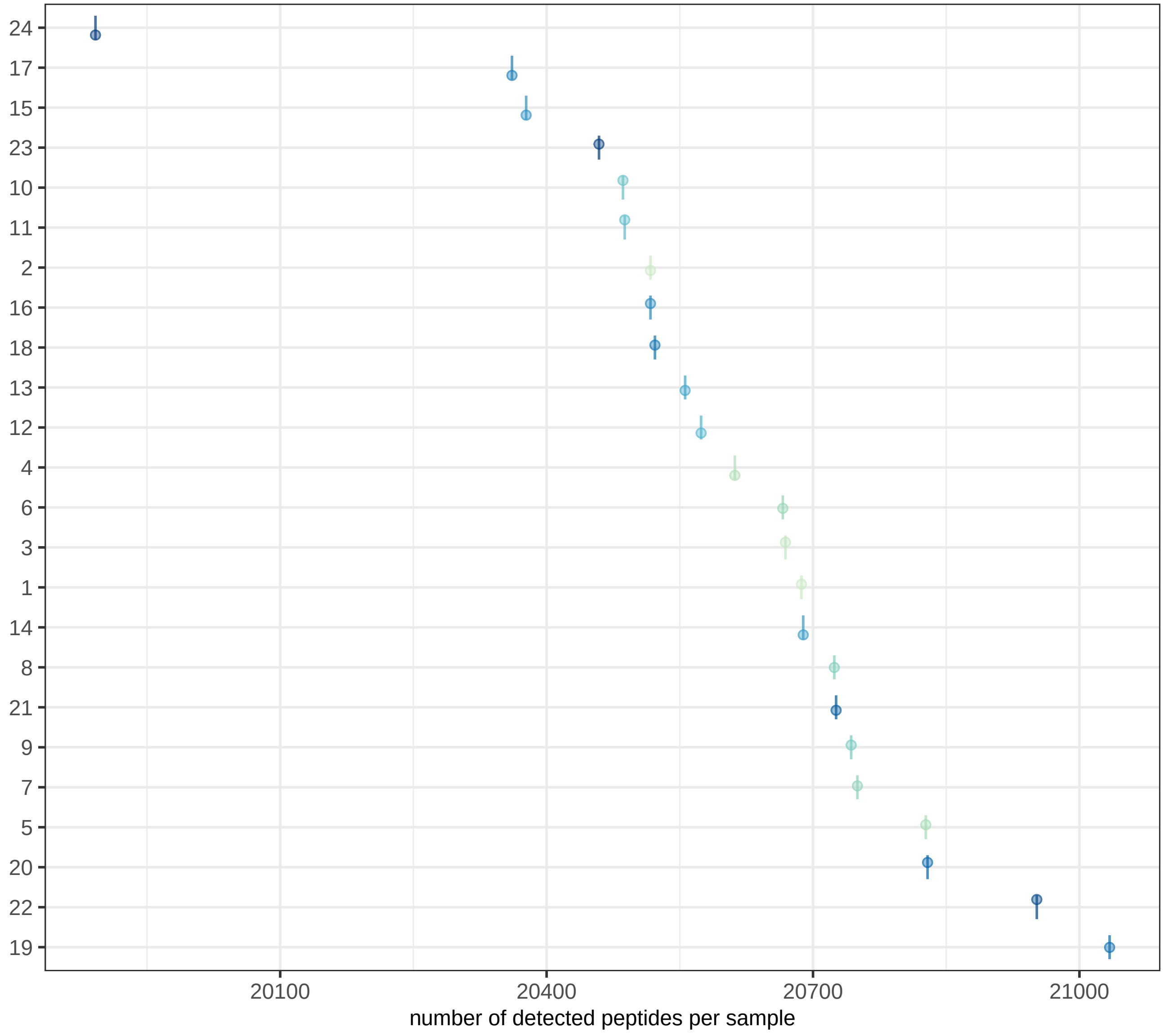

sample metadata used for color−coding: group

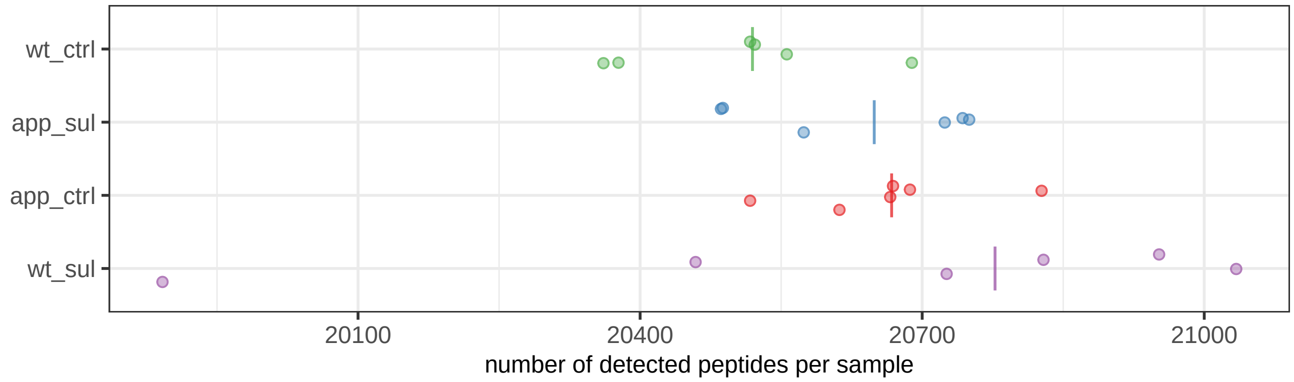

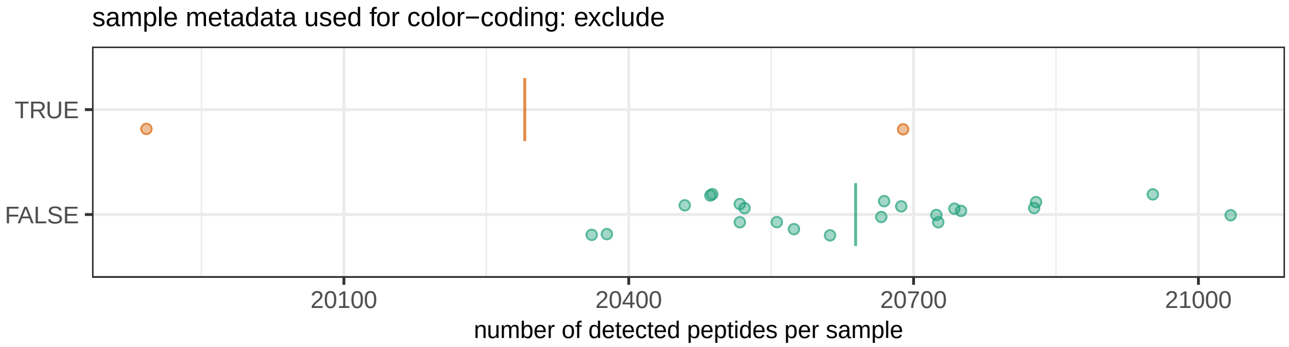

sample metadata used for color−coding: exclude
TRUE
FALSE
20100
20400
20700
21000
number of detected peptides per sample

1.3 data completeness

To visualize how many peptides are consistently identified in multiple samples, the first figure summarizes how common missing values are in the entire dataset. Optimally, most peptides are identified in 100% of samples and this curve slowly falls of. The following figure shows for each sample whether its peptides are also present in other samples in the dataset or whether these are unique to a (minor) subset of samples. You can use this mark of experimental consistency to compare datasets generated by similar protocols and mass-spec acquisition.

1.3.1 cumulative distribution

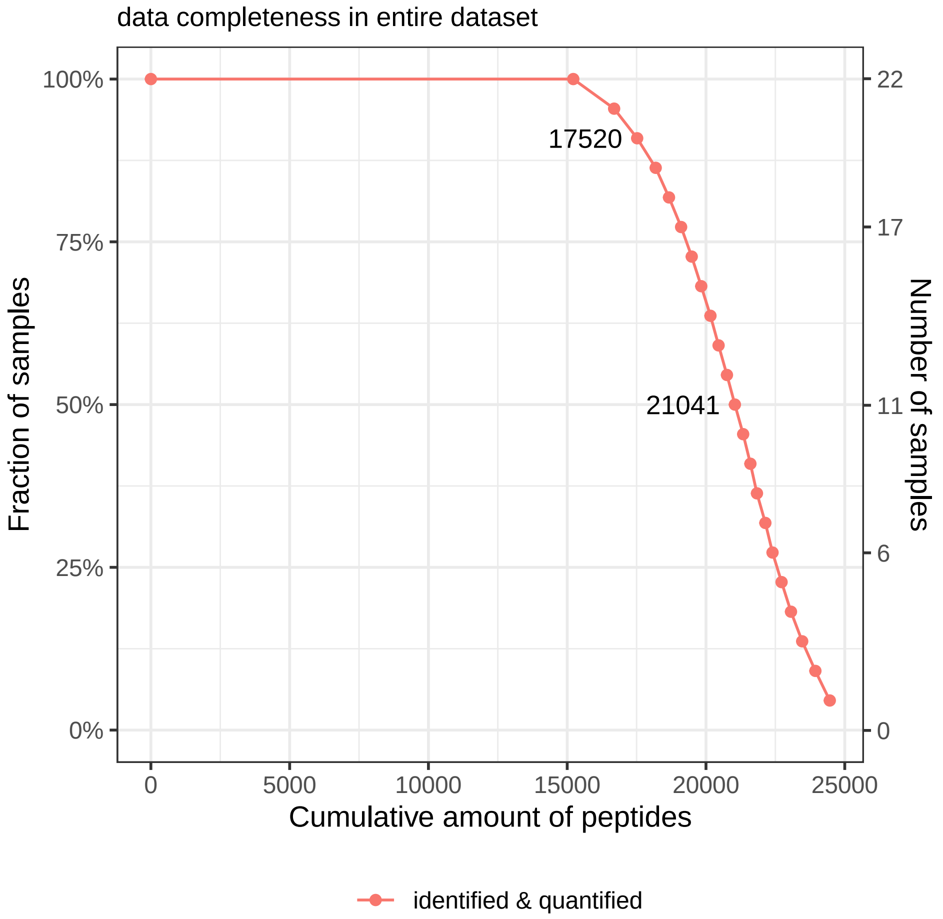

Samples flagged as ‘exclude’ (by user) are not taken into account in this figure. Exact values are shown for data points matching 90% and 50% of samples to convenience comparison between analyses (e.g. before/after configuring ‘exclude’ samples, or comparing between experiments of similar protocol).

1.3.2 peptide detection frequency

Each identified peptide in a sample is classified and color-coded by the number of other samples where the same peptide is present. Visualization of the amount of peptides that overlap with other samples in the dataset, from peptides identified in most samples (red) to one-hit-wonders (blue), helps identify uncommon samples (more blue/green than other samples).

Optimally, the majority of peptides in each sample are red~orange with relatively few uniquely identified peptides (blue~green). Samples are sorted by the total amount of detected peptides.

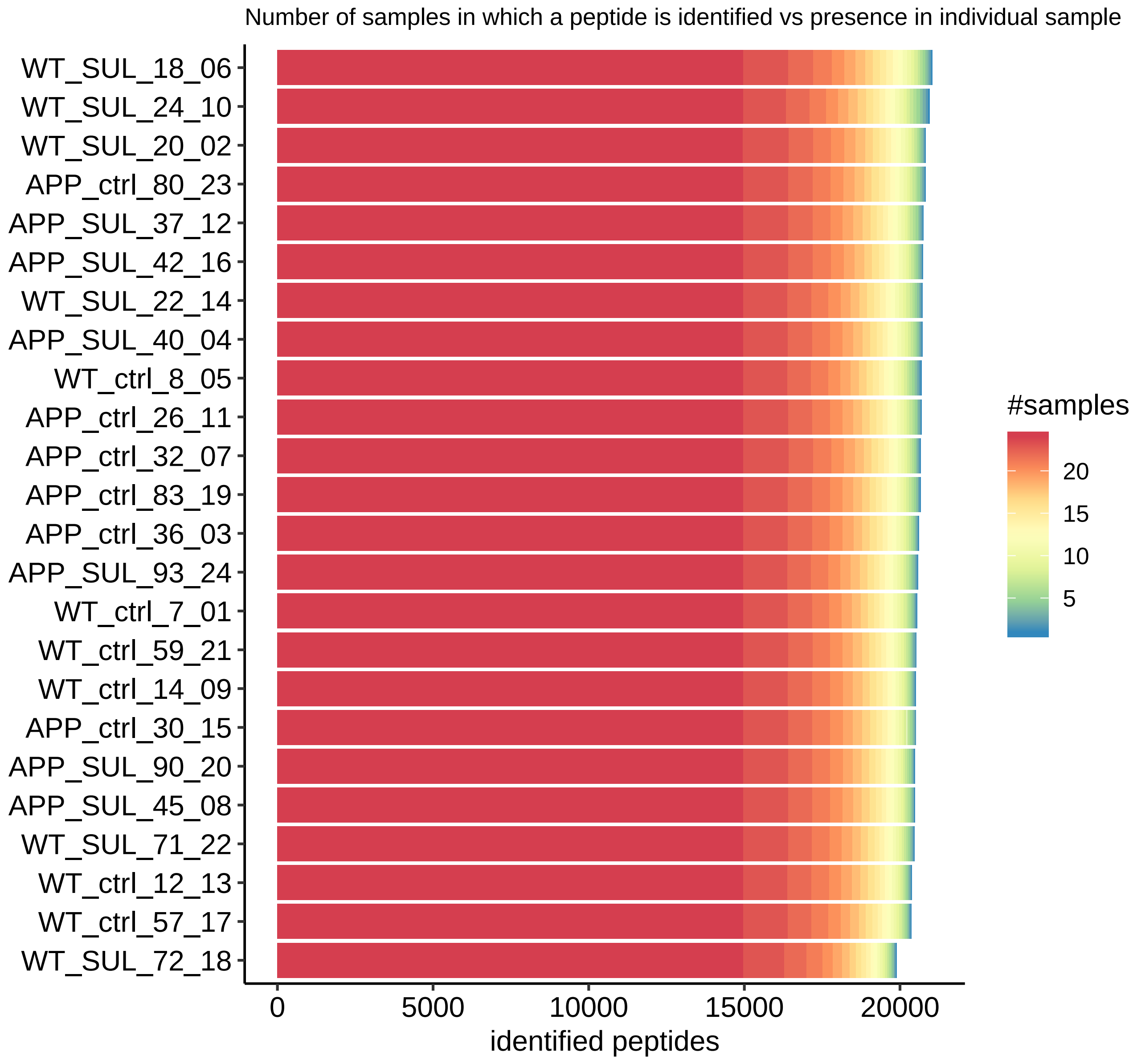

Number of samples in which a peptide is identified vs presence in individual sample
WT_SUL_18_06
WT_SUL_24_10
WT_SUL_20_02
APP_ctrl_80_23
APP_SUL_37_12
APP_SUL_42_16
WT_SUL_22_14
APP_SUL_40_04
WT_ctrl_8_05
APP_ctrl_26_11
APP_ctrl_32_07
APP_ctrl_83_19
APP_ctrl_36_03
APP_SUL_93_24
WT_ctrl_7_01
WT_ctrl_59_21
WT_ctrl_14_09
APP_ctrl_30_15
APP_SUL_90_20
APP_SUL_45_08
WT_SUL_71_22
WT_ctrl_12_13
WT_ctrl_57_17
WT_SUL_72_18
0
5000
10000
15000
20000
identified peptides
#samples
20
15
10
5

1.4 abundance distributions

The figures in this subsection are used to identify unexpected mass-spec sensitivity or sample loading differences. Peptide data is shown as provided in input files, so peptide filtering nor intensity normalization has been applied yet (for proper QC, make sure the software that generated the input data did not apply normalization prior). If the dataset is DDA, match-between-runs (MBR) peptides are included in these distributions whereas for DIA only 'detected' peptides (based on confidence score threshold) are included.

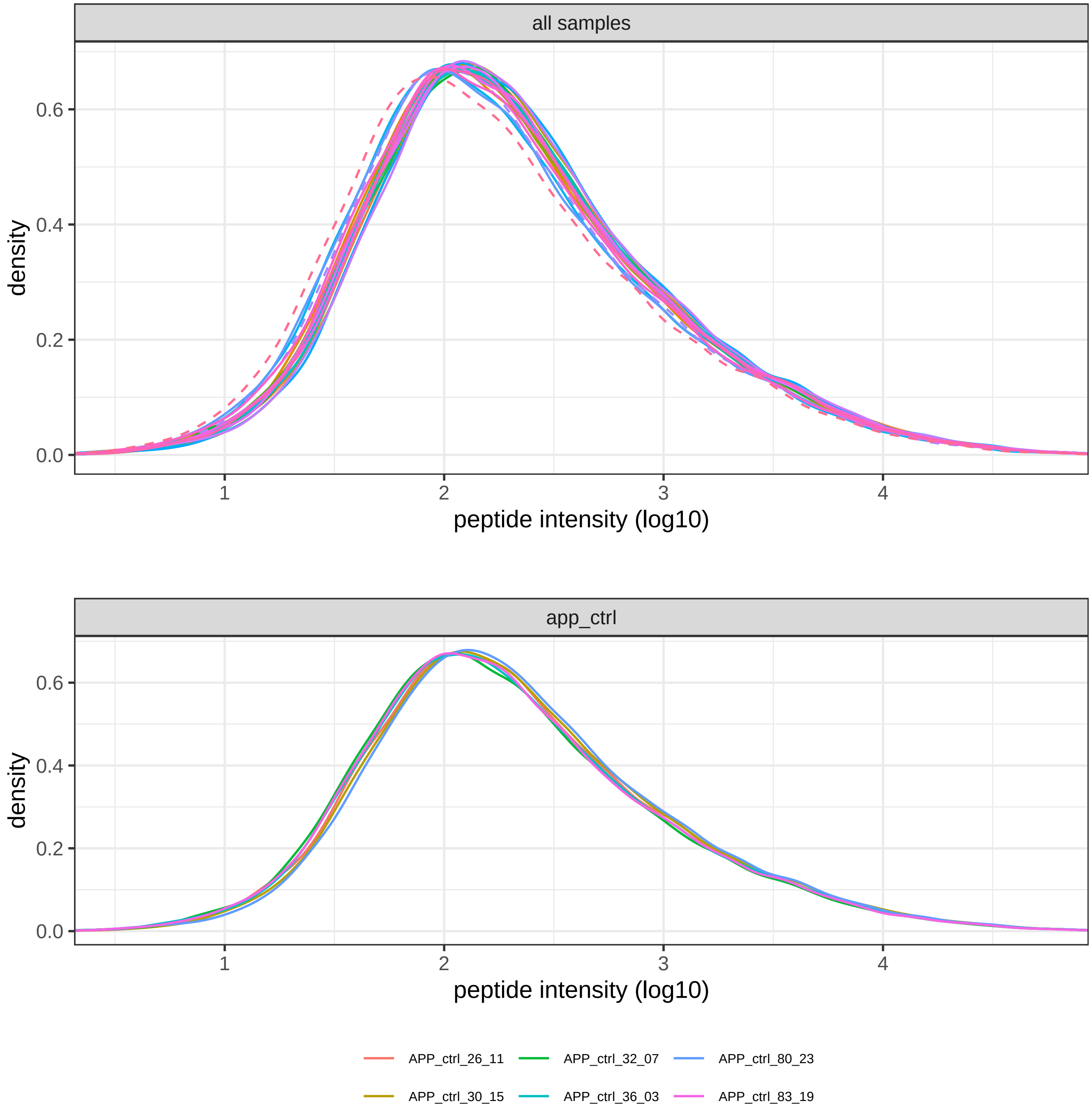

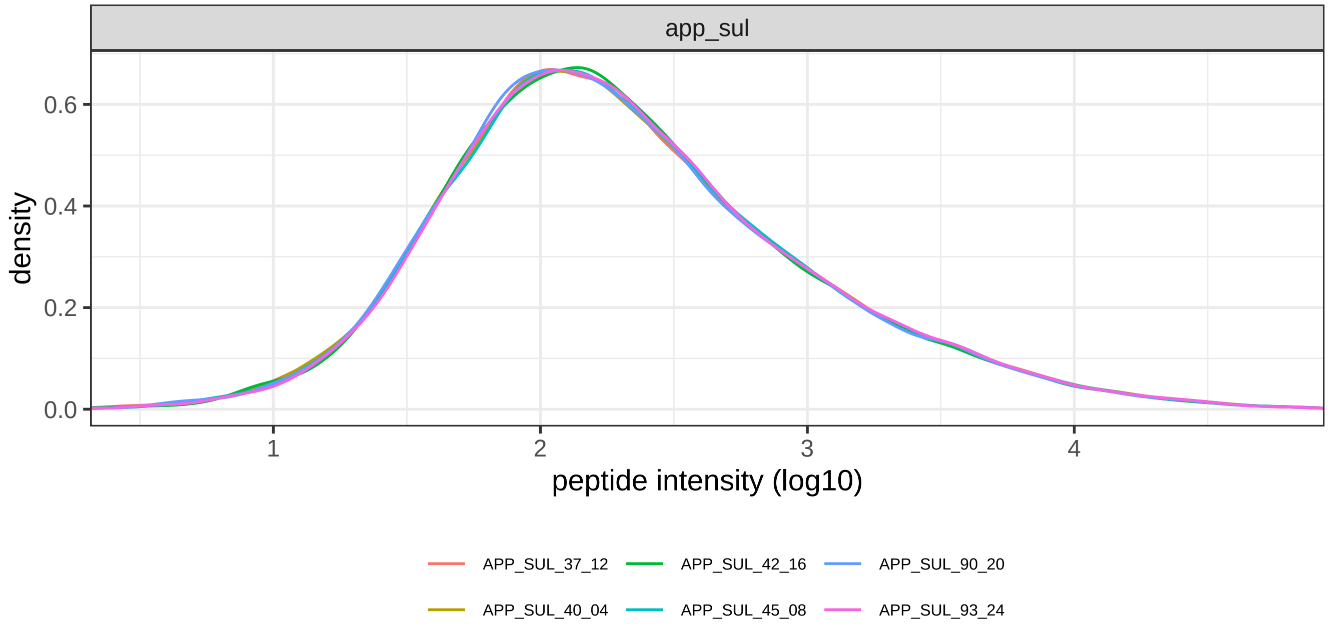
app_sul
density
0.0
0.2
0.4
0.6
1
2
3
4
peptide intensity (log10)
APP_SUL_37_12
APP_SUL_42_16
APP_SUL_90_20
APP_SUL_40_04
APP_SUL_45_08
APP_SUL_93_24

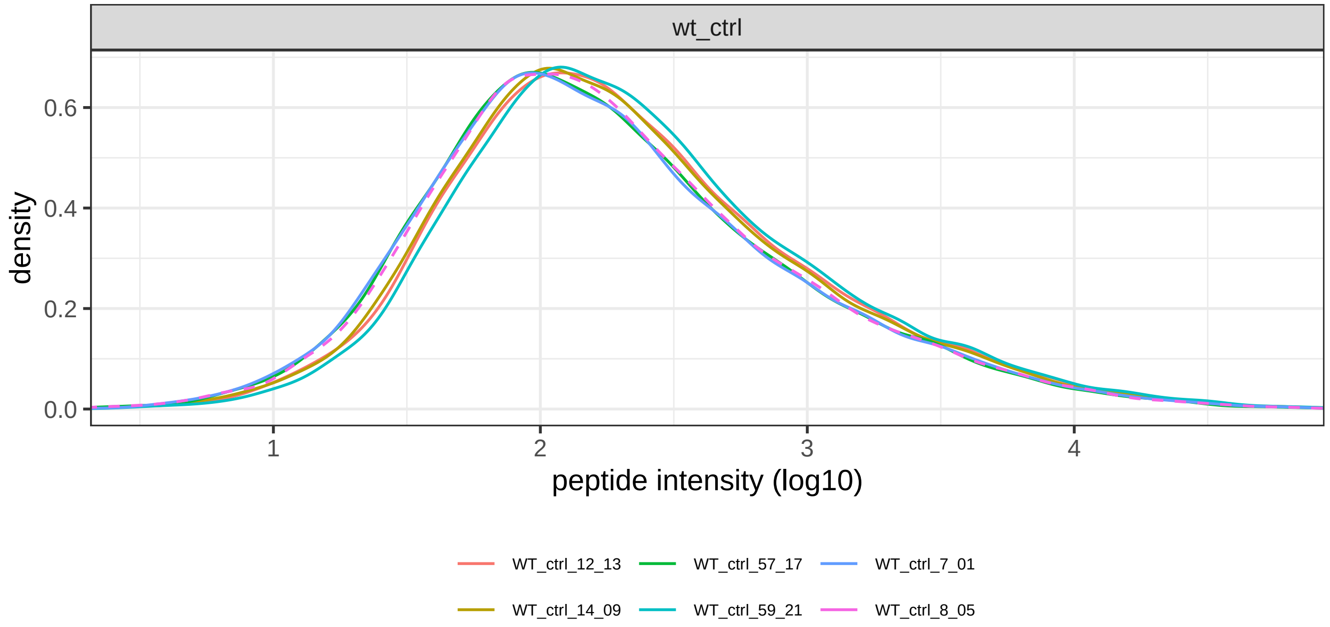
wt_ctrl
density
0.0
0.2
0.4
0.6
1
2
3
4
peptide intensity (log10)
WT_ctrl_12_13
WT_ctrl_57_17
WT_ctrl_7_01
WT_ctrl_14_09
WT_ctrl_59_21
WT_ctrl_8_05

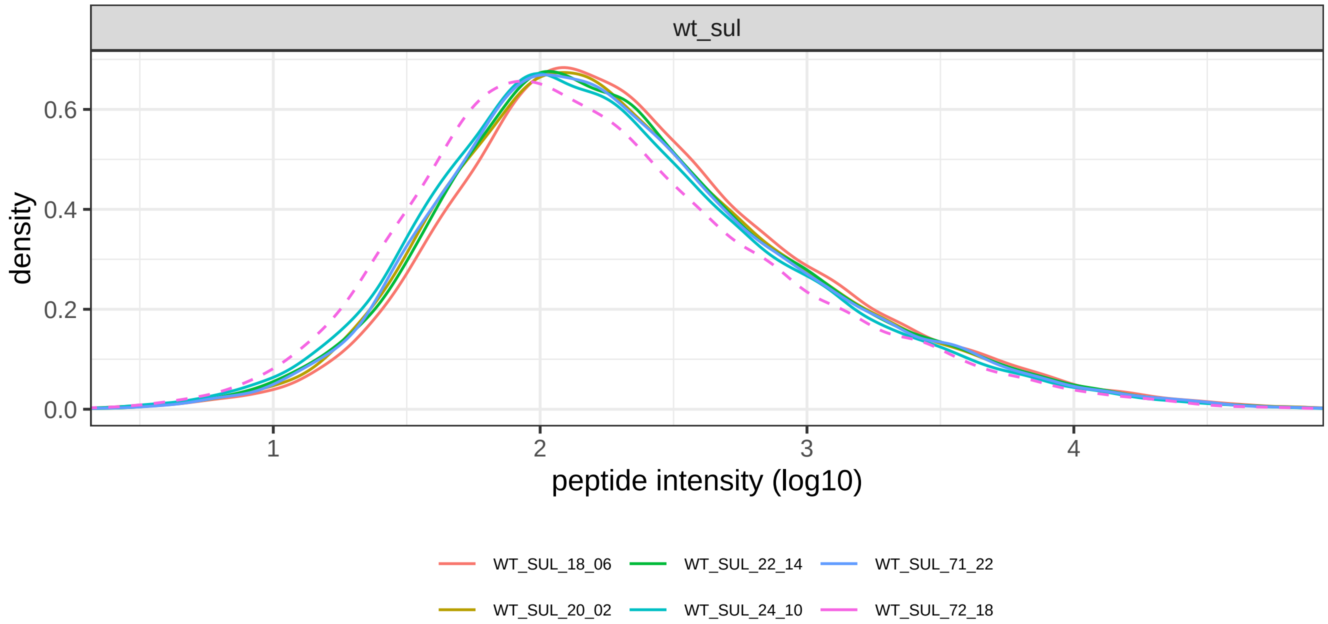
wt_sul
density
0.6
0.4
0.2
0.0
1
2
3
4
peptide intensity (log10)
WT_SUL_18_06
WT_SUL_22_14
WT_SUL_71_22
WT_SUL_20_02
WT_SUL_24_10
WT_SUL_72_18

1.5 retention time

The figures in this section allow you to identify potential problems during HPLC elution, such as a temporarily blocking column, failing ionization spray or decreasing sensitivity over time. For each sample, all peptides that are also observed in a replicate (such that there is a point of reference available) are visualized.

1.5.1 retention time distributions

The density of the number of peptides eluting at each point in time. The figure below presents an overview of all samples that allows for the identification of outlier samples that follow distinct elution patterns. The following section shows details for each sample. Samples marked as 'exclude' in the provided sample metadata table are visualized as dashed lines.

progress: RT plots: preparing data took 36 seconds progress: RT plots: creating plots took 8 seconds

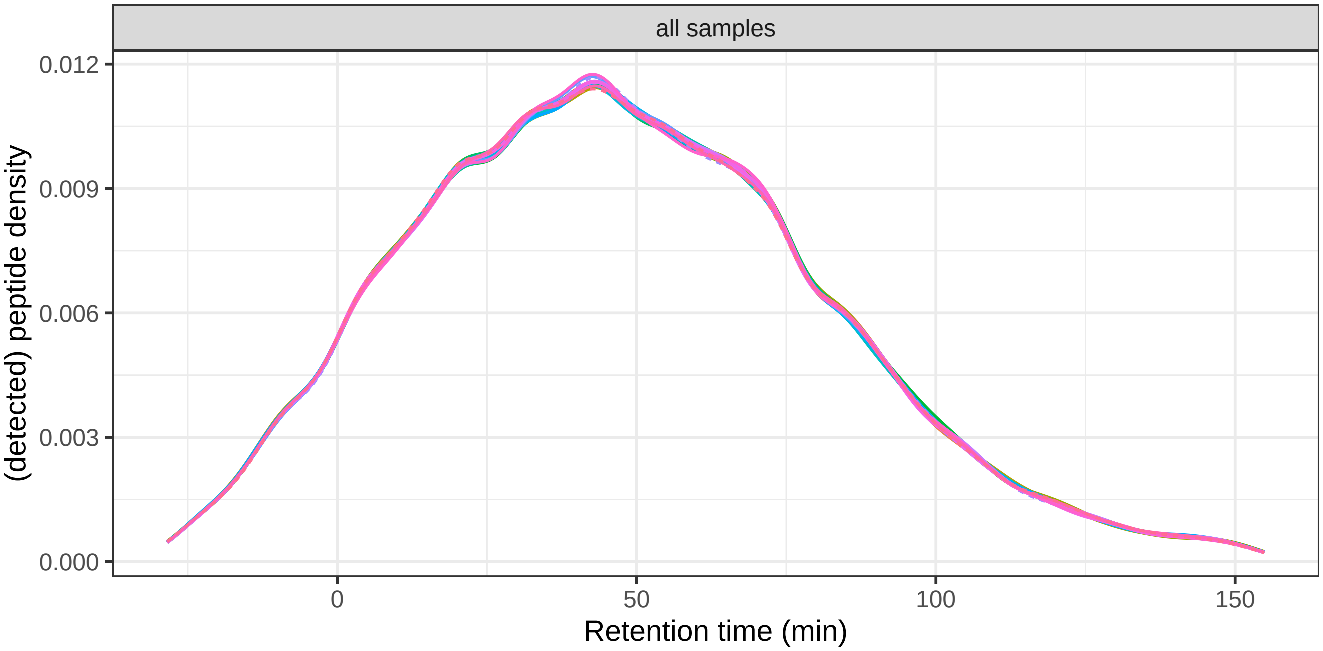

1.5.2 retention time local effects

To investigate how each measurement differs from others, we visualize each sample as a 3 panel figure. First, the data is binned across the retention time dimension (x-axis). If a samples was marked as 'exclude' in the provided sample metadata, this is indicated in the plot title.

The top panel shows the number of peptides in the input data, e.g. as recognized by the software that generated input for this pipeline, over time (black line). For reference, the grey line shows the median amount over all samples (note; if this is the exact same in all samples, the grey line may not be visible as it falls behind the black line).

The middle panel indicates whether peptide retention times deviate from their median over all samples (blue line). The grey area depicts the 5% and 95% quantiles, respectively. The line width corresponds to the number of peptides eluting at that time (data from first panel). Analogously, the bottom panel shows the deviation in peptide abundance as compared to the median over all samples (red line).

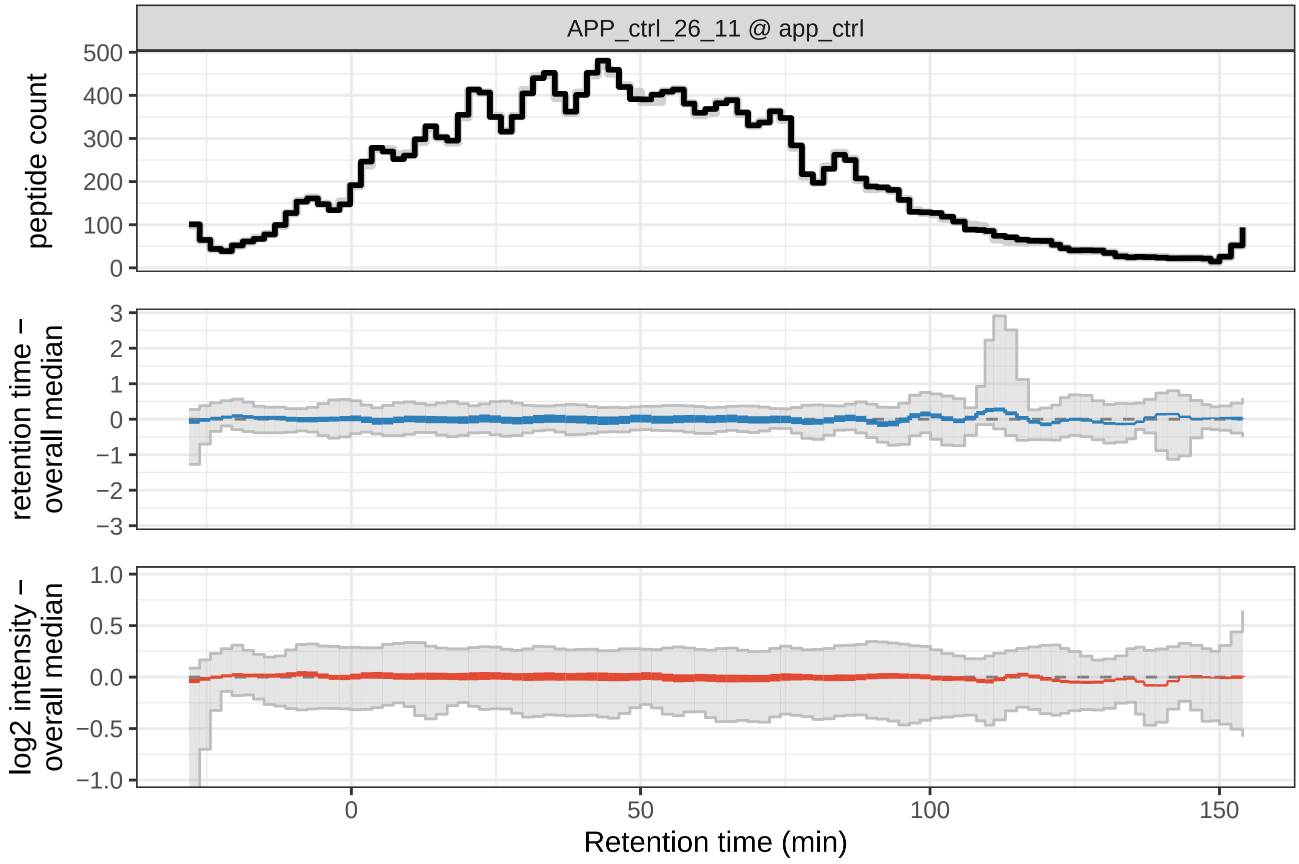
APP_ctrl_26_11 @ app_ctrl
peptide count
retention time – overall median
log2 intensity – overall median
Retention time (min)
0
50
100
150

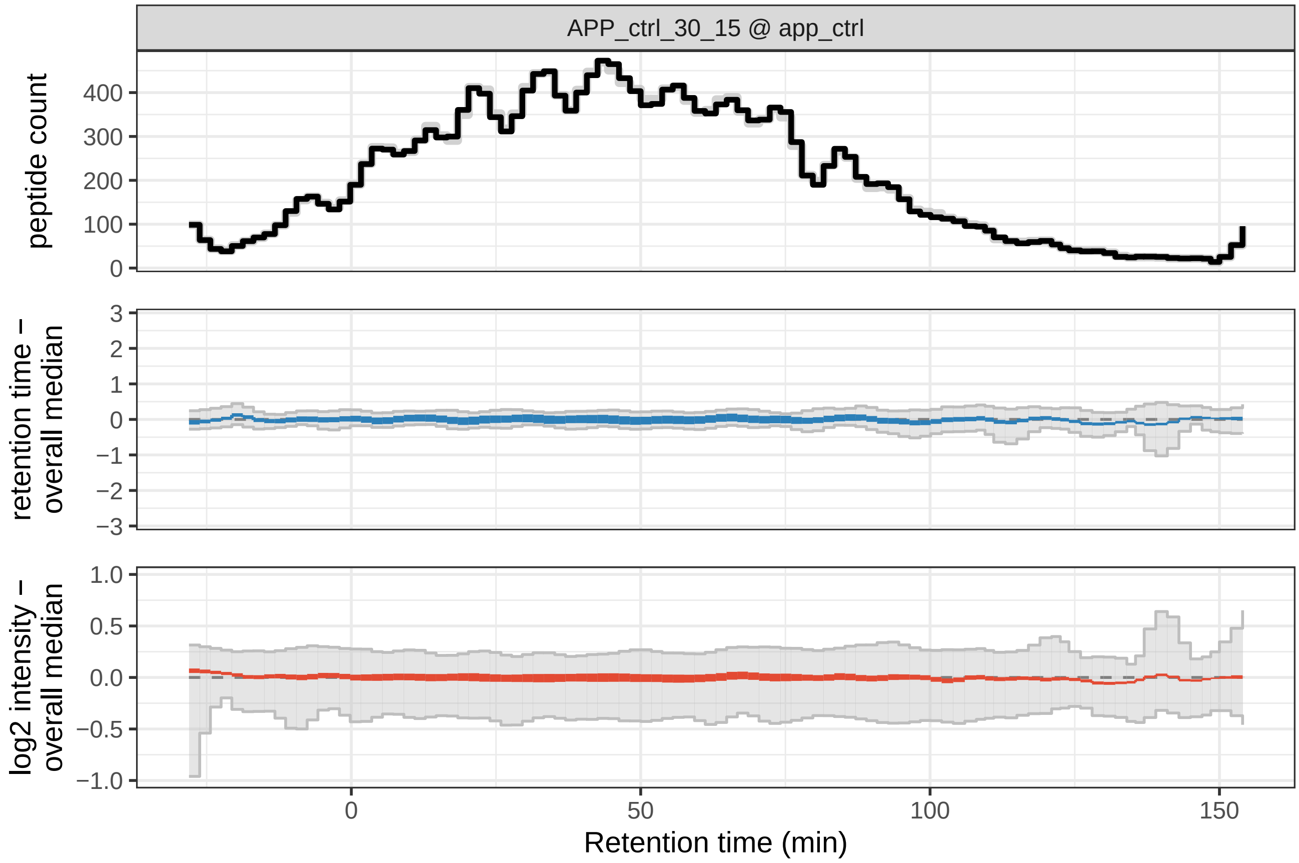
APP_ctrl_30_15 @ app_ctrl
peptide count
retention time – overall median
log2 intensity – overall median
Retention time (min)
0
50
100
150

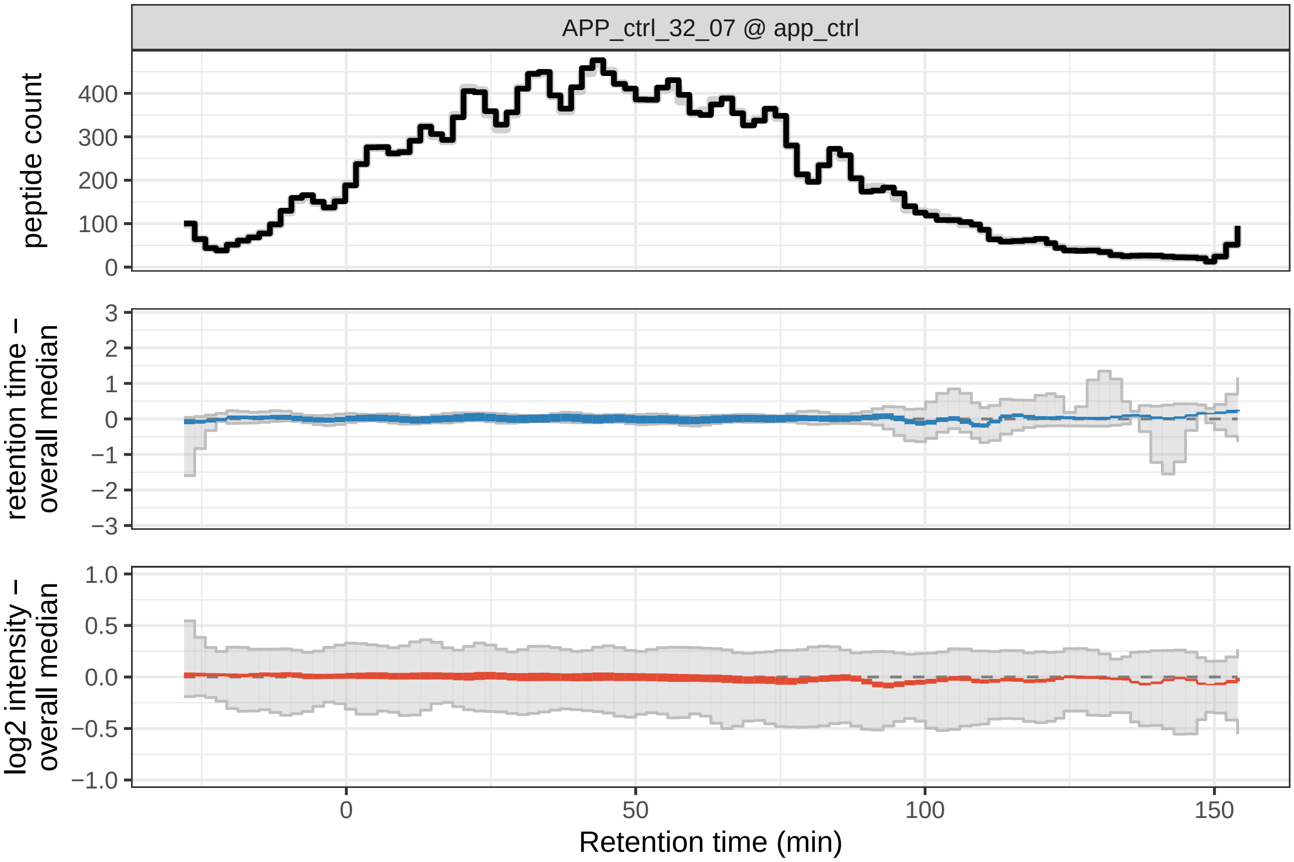
APP_ctrl_32_07 @ app_ctrl
peptide count
retention time – overall median
log2 intensity – overall median
Retention time (min)

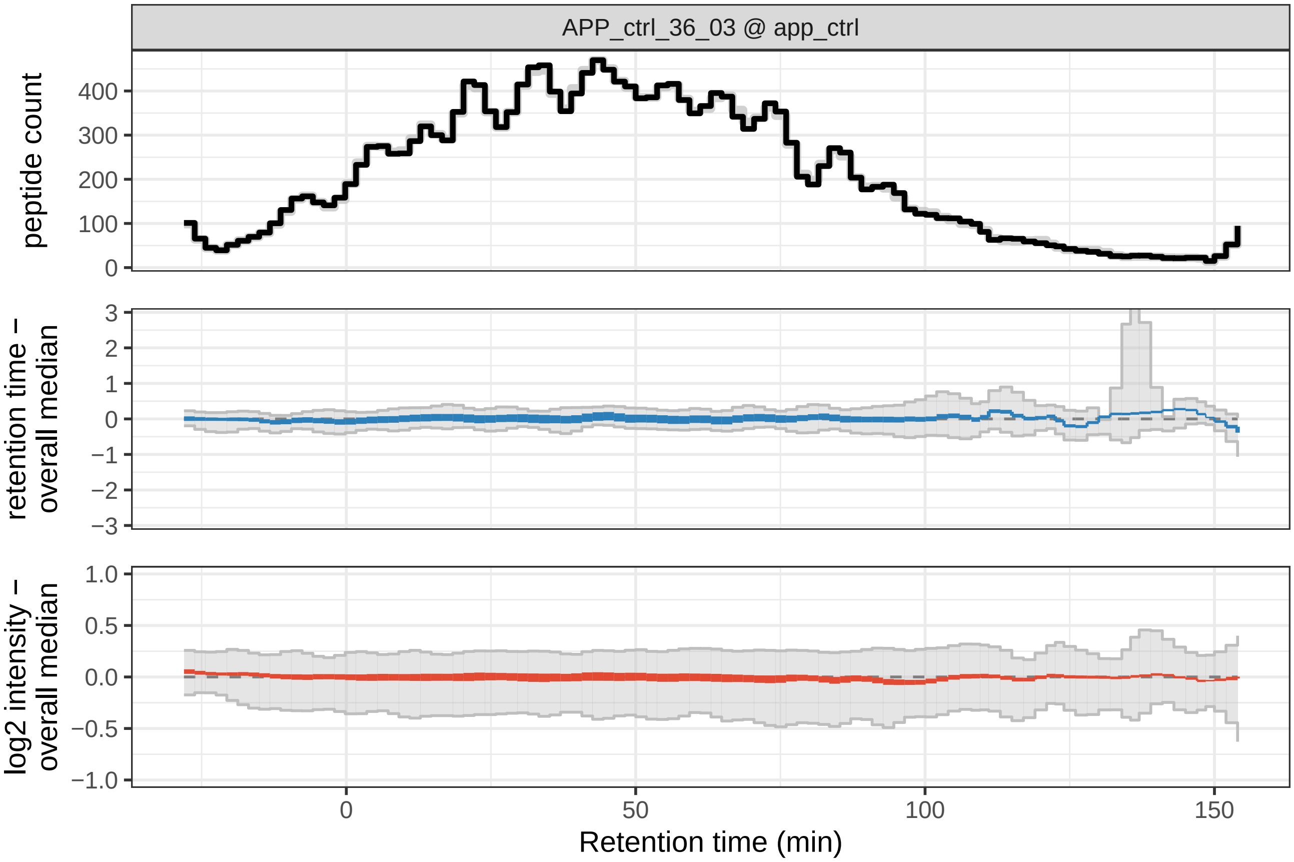
APP_ctrl_36_03 @ app_ctrl
peptide count
retention time – overall median
log2 intensity – overall median
Retention time (min)

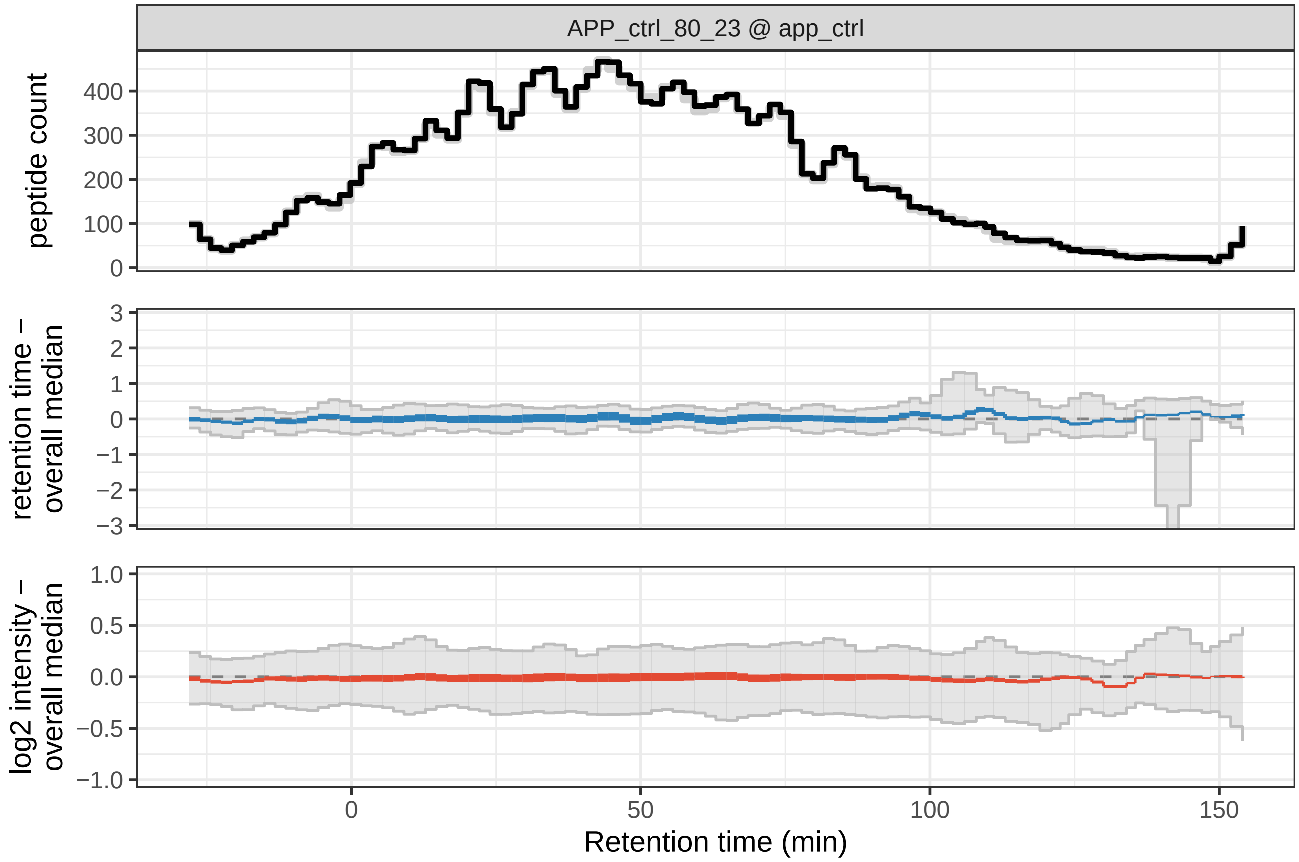
APP_ctrl_80_23 @ app_ctrl
peptide count
retention time – overall median
log2 intensity – overall median
Retention time (min)

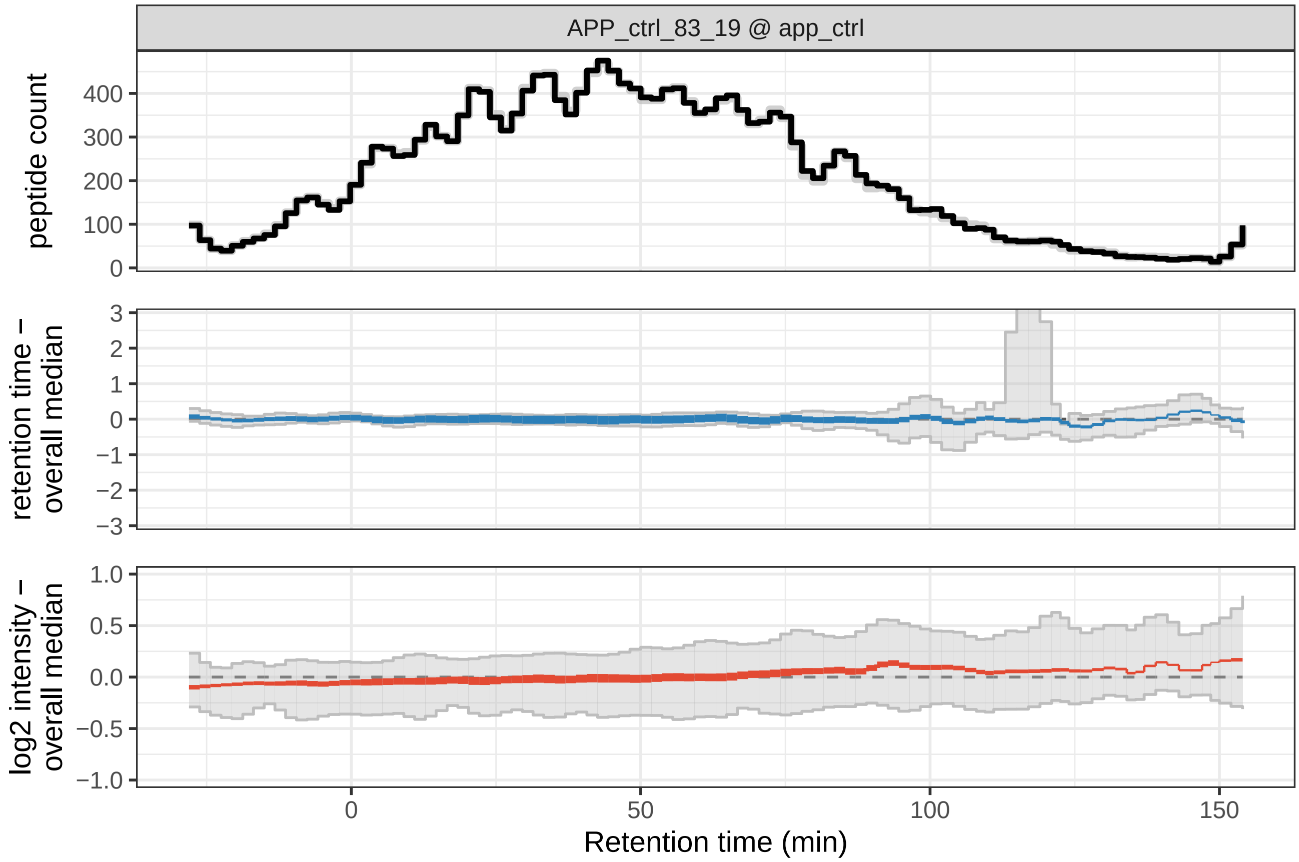
APP_ctrl_83_19 @ app_ctrl
peptide count
retention time – overall median
log2 intensity – overall median
Retention time (min)

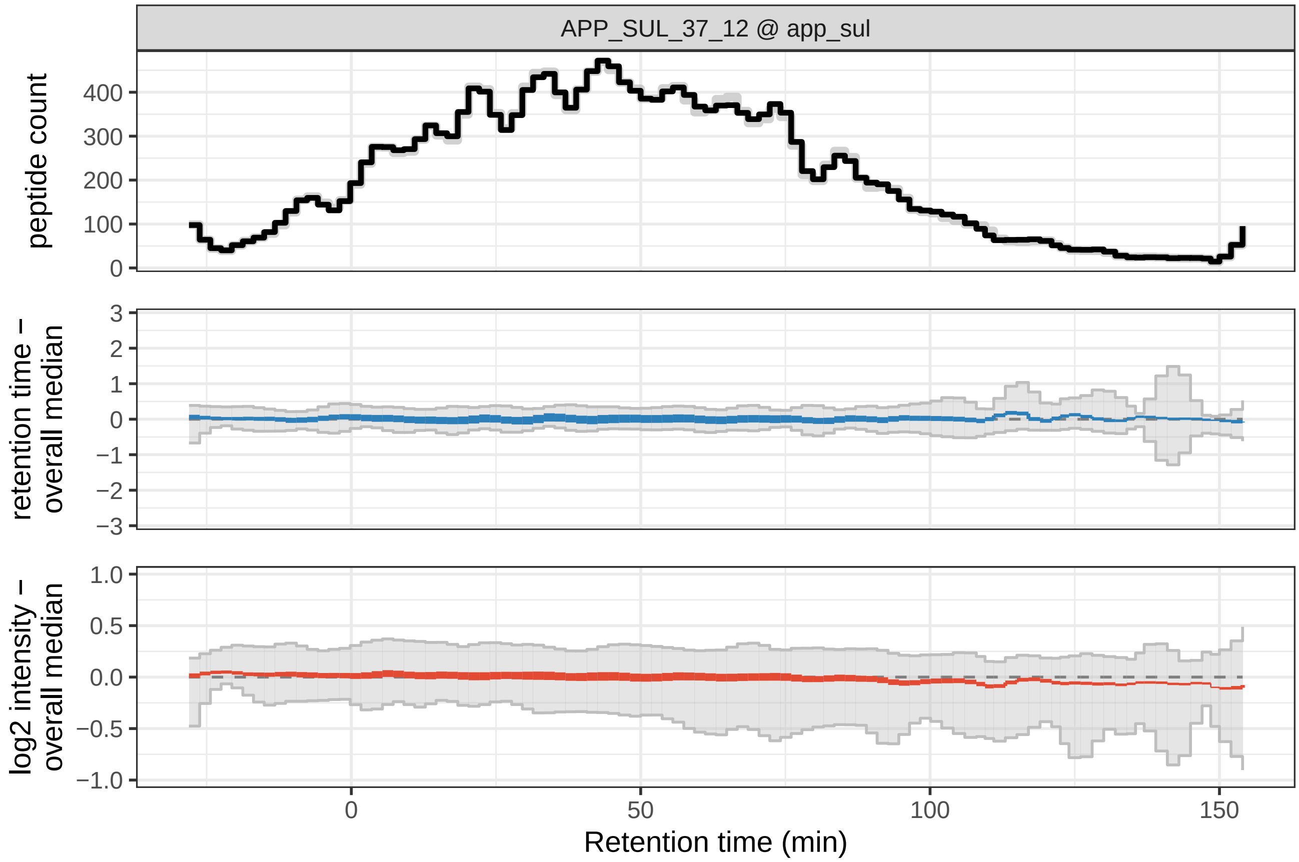

APP_SUL_37_12 @ app_sul
peptide count
retention time – overall median
log2 intensity – overall median
Retention time (min)

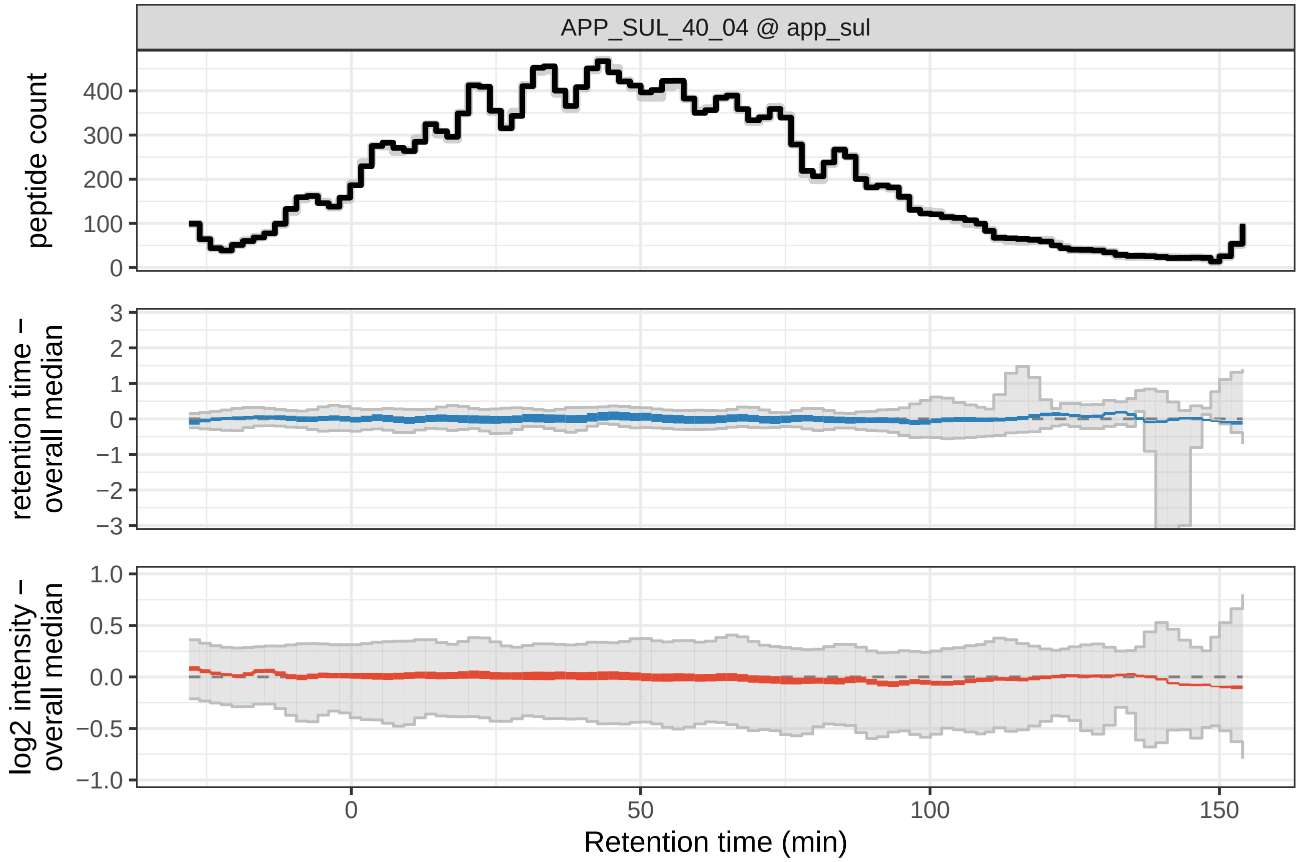

APP_SUL_40_04 @ app_sul
peptide count
retention time – overall median
log2 intensity – overall median
Retention time (min)

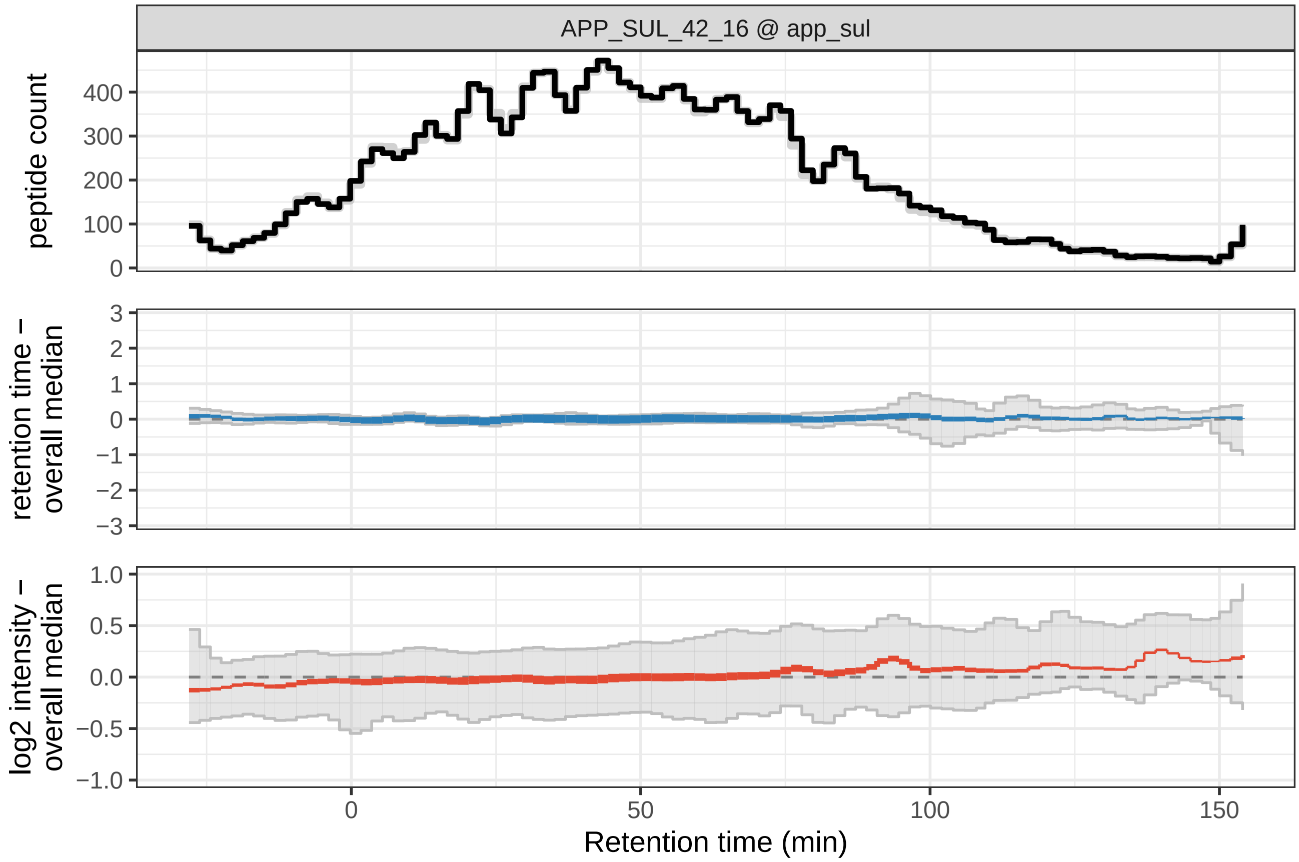
APP_SUL_42_16 @ app_sul
peptide count
retention time – overall median
log2 intensity – overall median
Retention time (min)

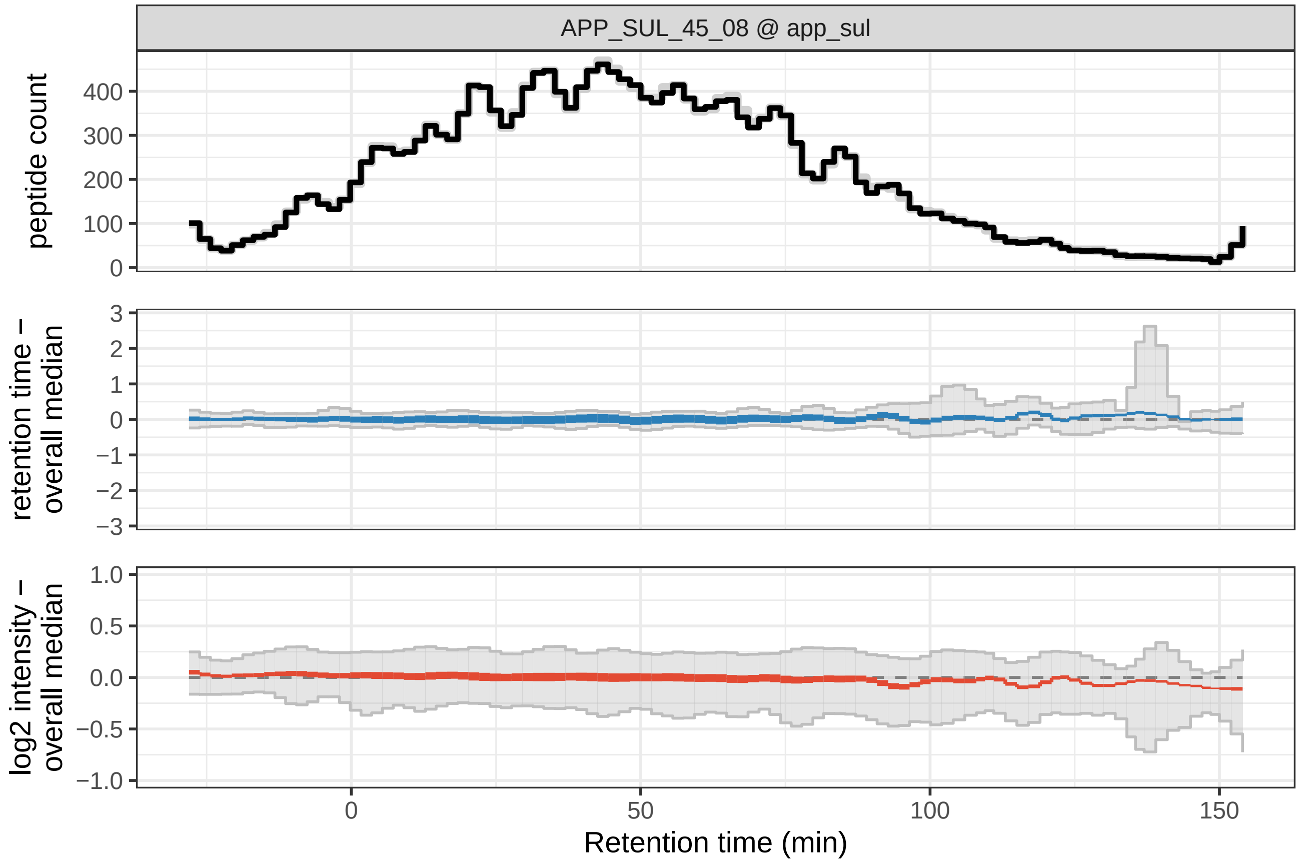
APP_SUL_45_08 @ app_sul
peptide count
retention time – overall median
log2 intensity – overall median
Retention time (min)

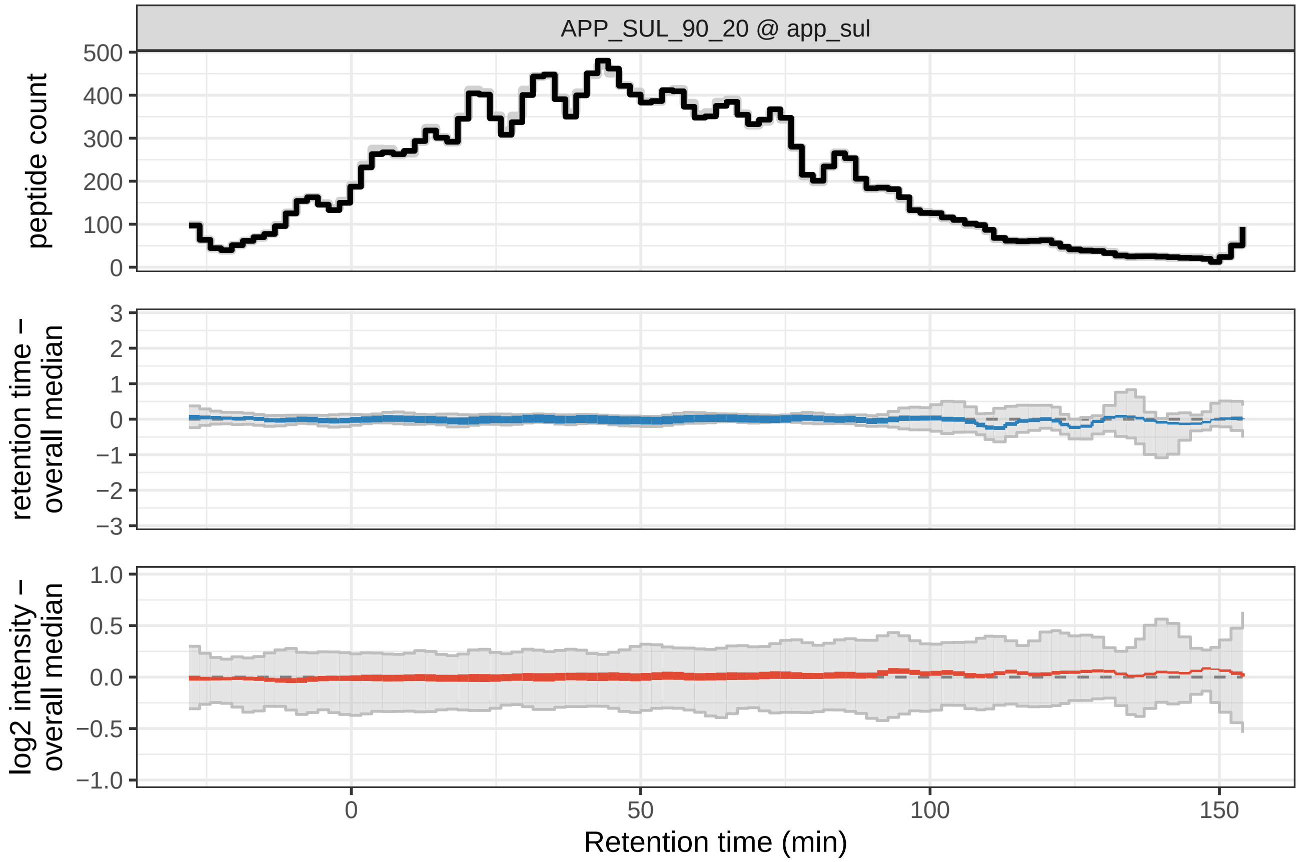
APP_SUL_90_20 @ app_sul
peptide count
0
100
200
300
400
500
retention time –
overall median
3
2
1
0
−1
−2
−3
log2 intensity –
overall median
1.0
0.5
0.0
−0.5
−1.0
0
50
100
150
Retention time (min)

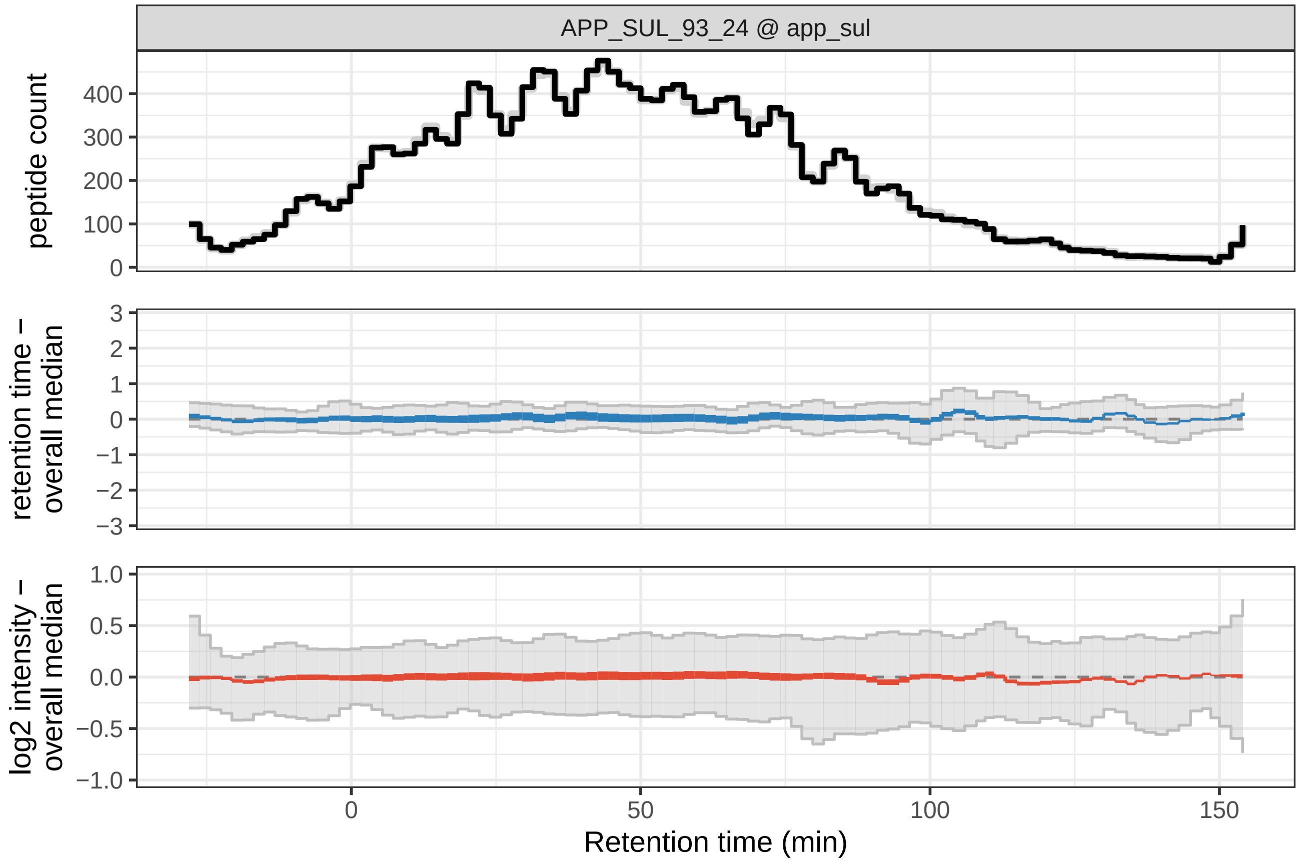
APP_SUL_93_24 @ app_sul
peptide count
0
100
200
300
400
retention time –
overall median
3
2
1
0
−1
−2
−3
log2 intensity –
overall median
1.0
0.5
0.0
−0.5
−1.0
0
50
100
150
Retention time (min)

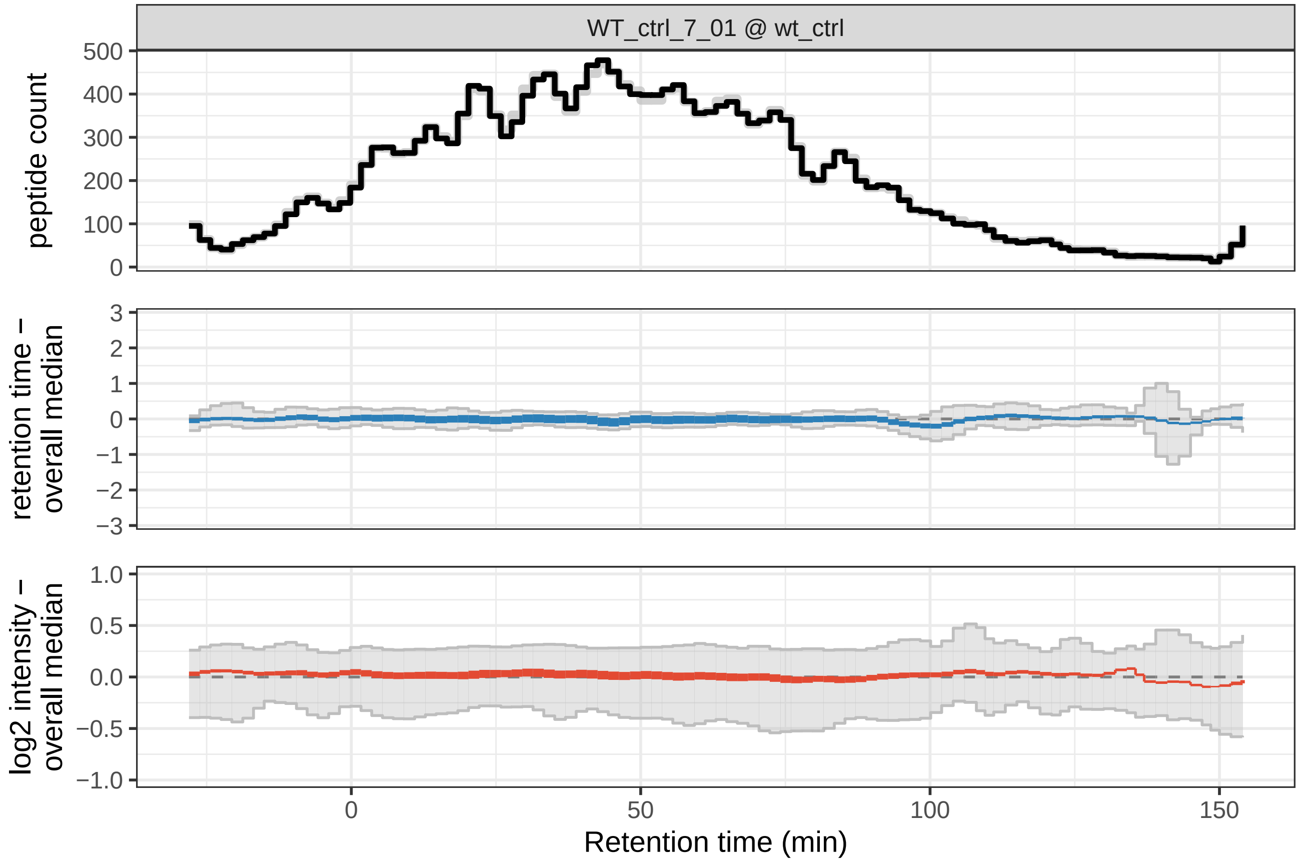
WT_ctrl_7_01 @ wt_ctrl
peptide count
retention time – overall median
log2 intensity – overall median
Retention time (min)
0
50
100
150

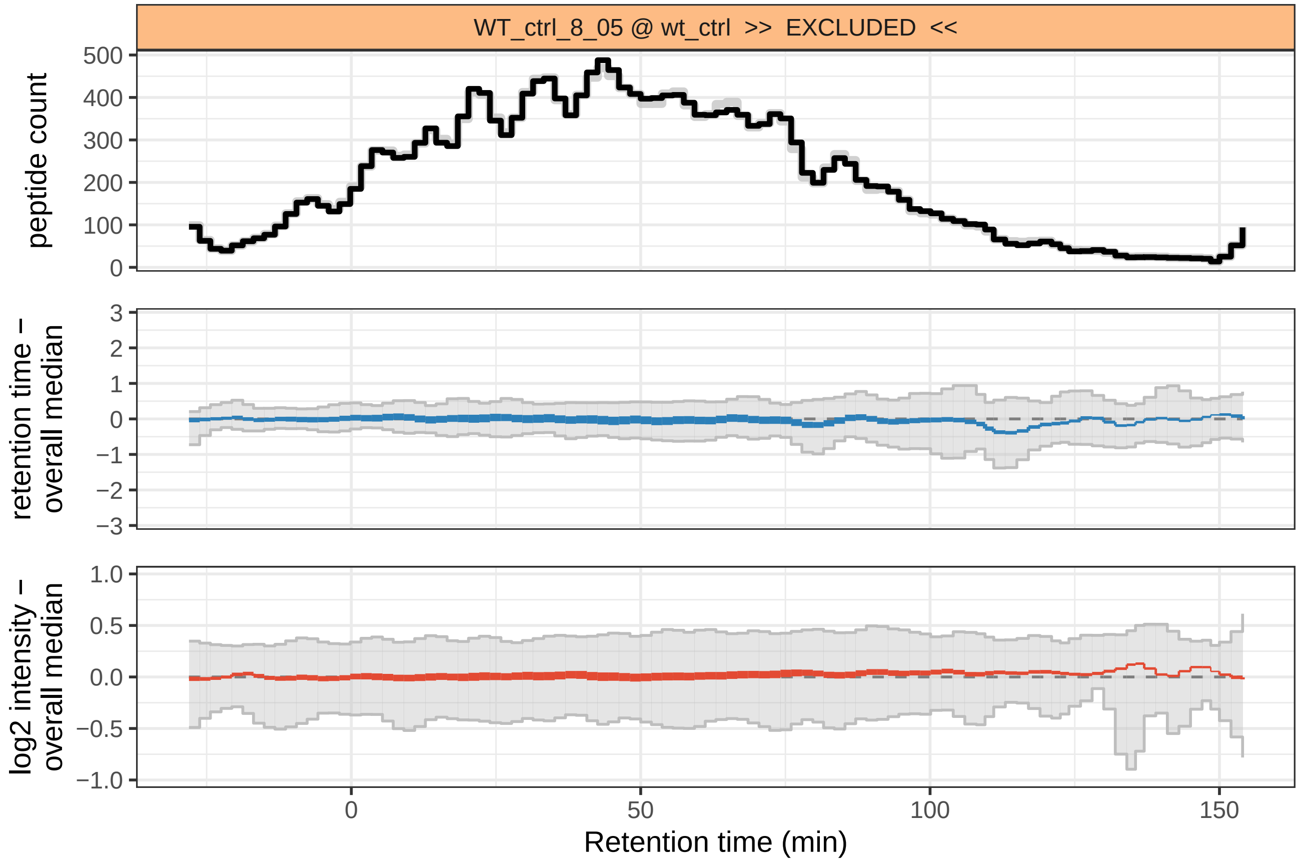
WT_ctrl_8_05 @ wt_ctrl >> EXCLUDED <<
peptide count
retention time – overall median
log2 intensity – overall median
Retention time (min)
0
50
100
150

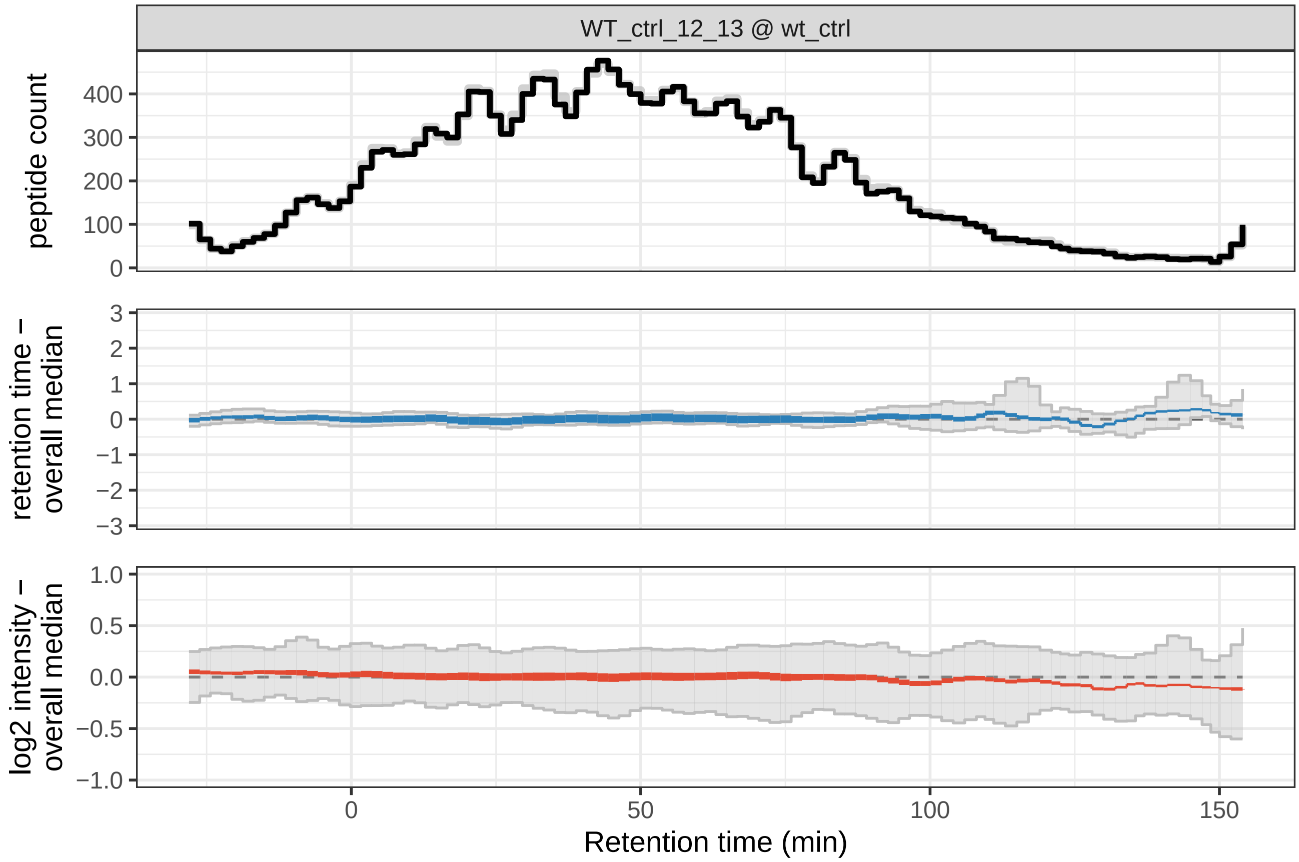
WT_ctrl_12_13 @ wt_ctrl
peptide count
retention time – overall median
log2 intensity – overall median
Retention time (min)

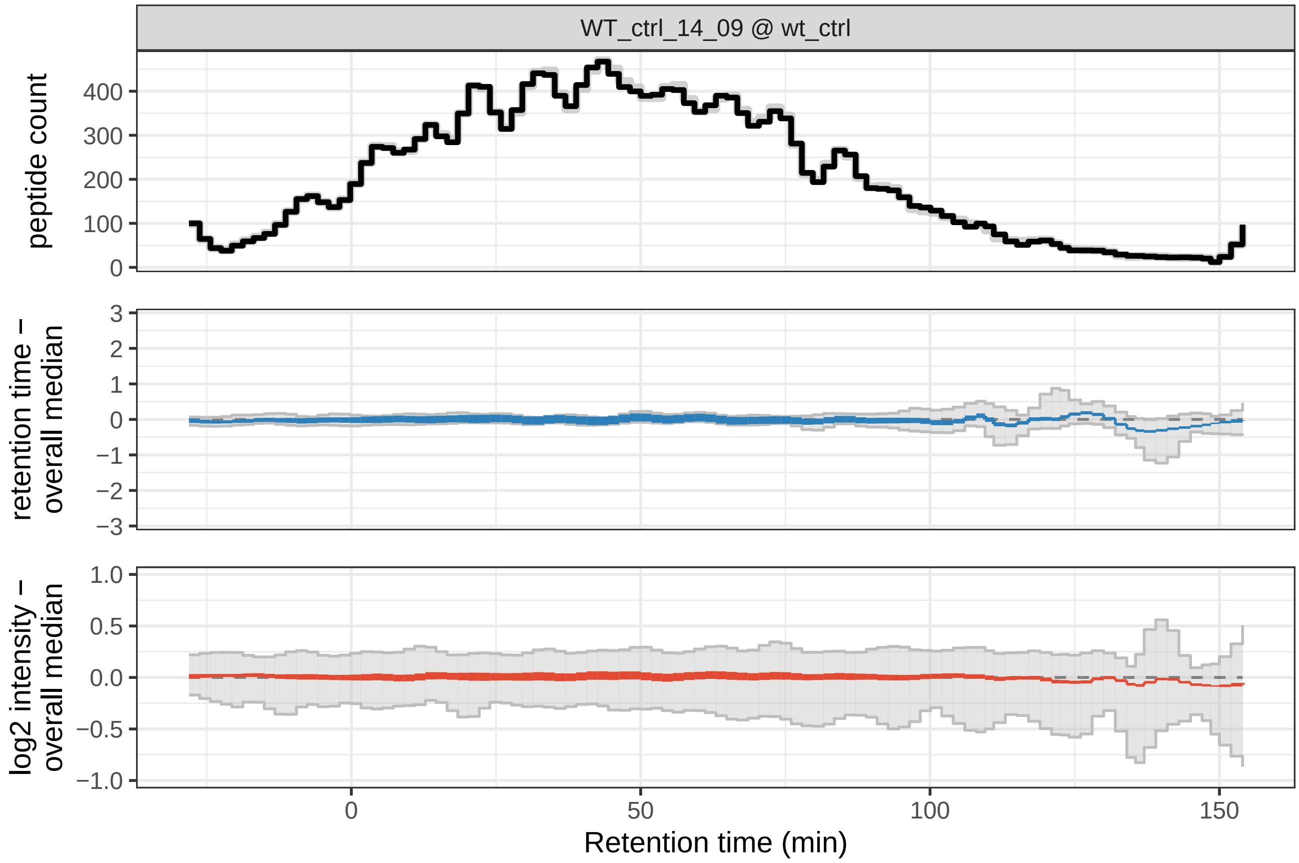
WT_ctrl_14_09 @ wt_ctrl
peptide count
retention time – overall median
log2 intensity – overall median
Retention time (min)

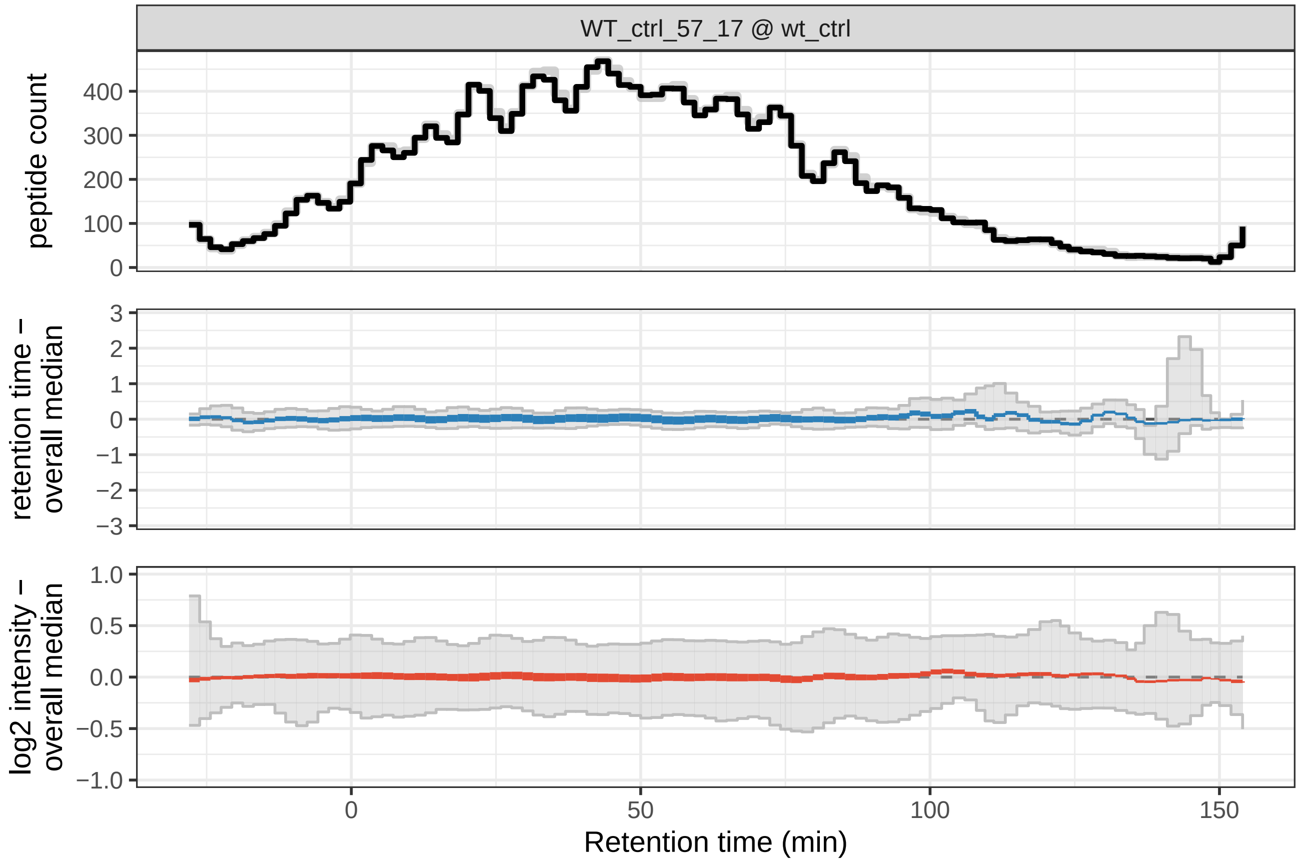
WT_ctrl_57_17 @ wt_ctrl
peptide count
retention time – overall median
log2 intensity – overall median
Retention time (min)

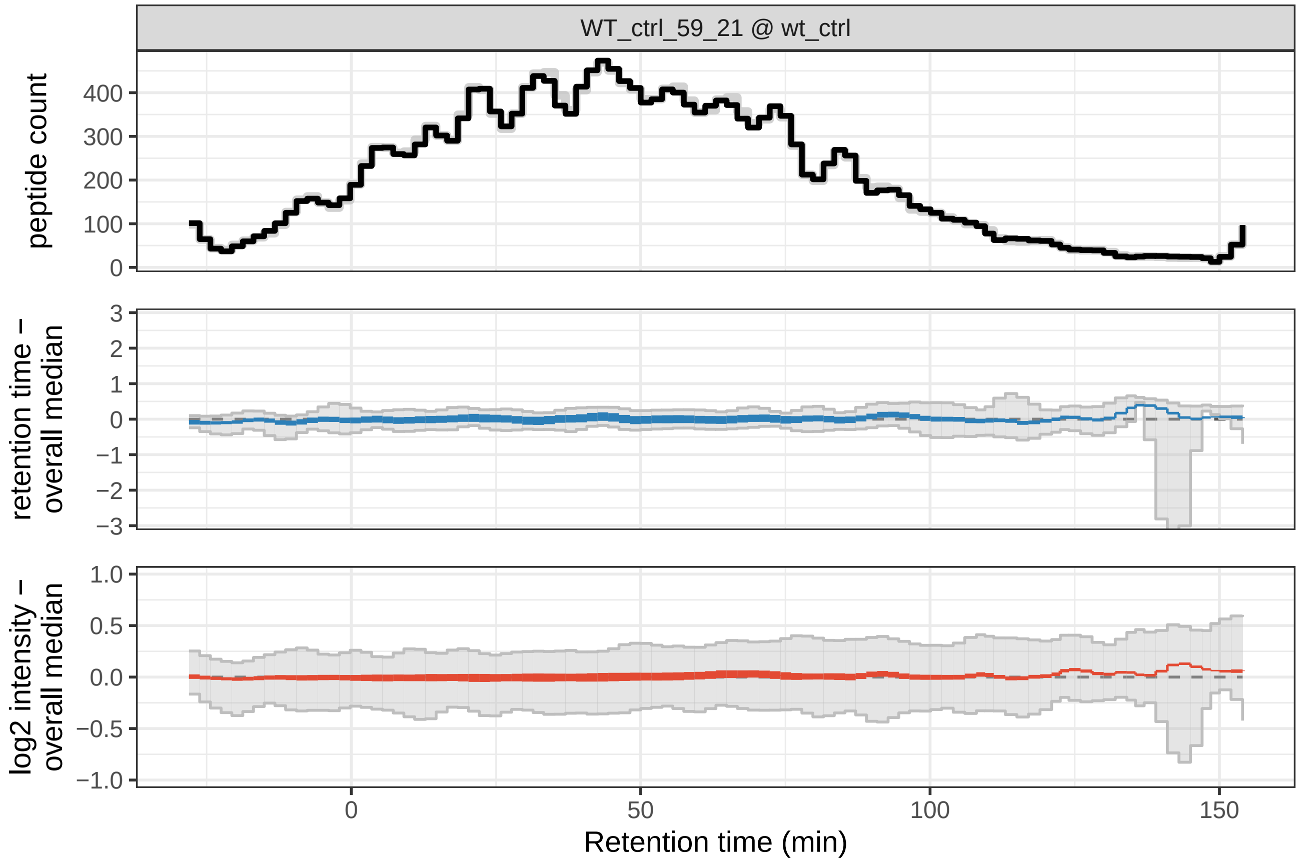
WT_ctrl_59_21 @ wt_ctrl
peptide count
retention time – overall median
log2 intensity – overall median
Retention time (min)

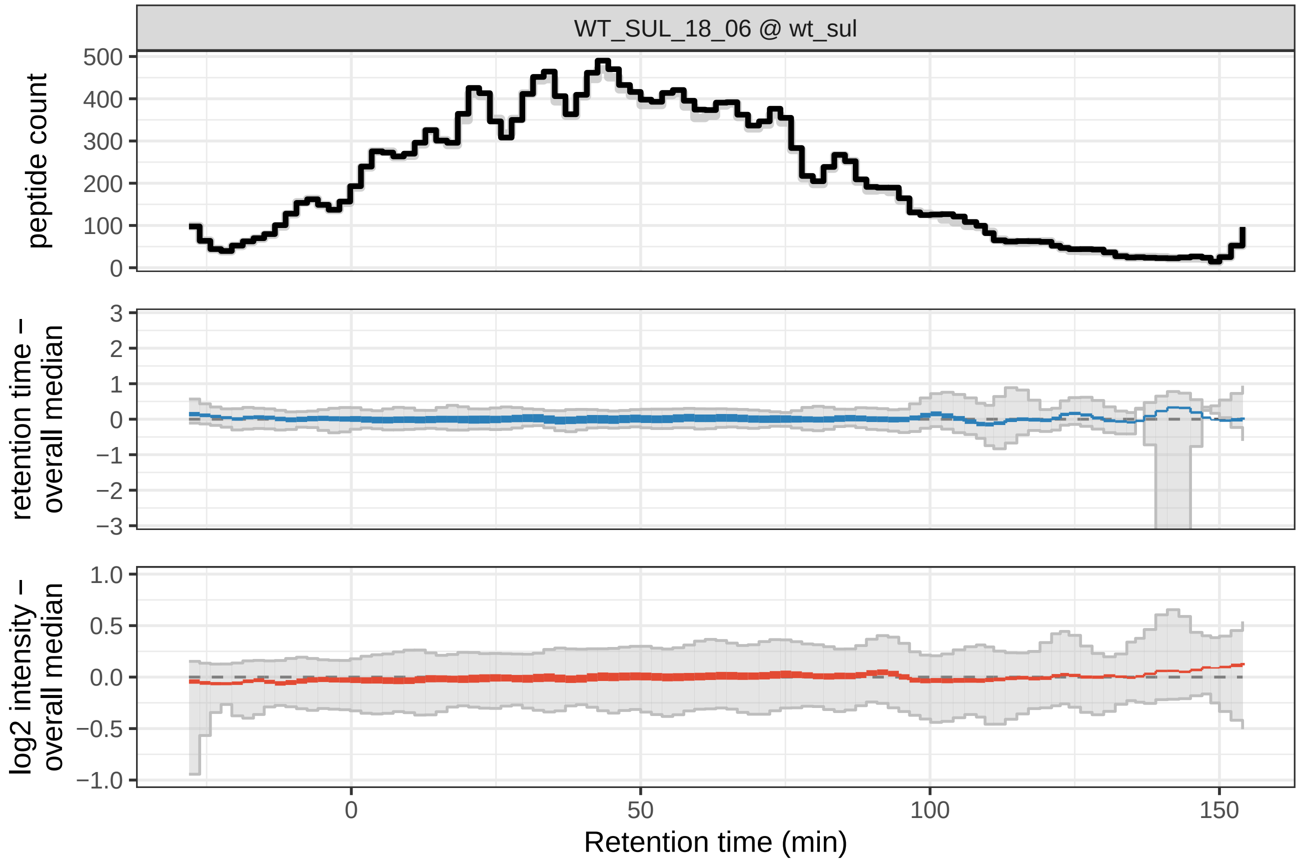
WT_SUL_18_06 @ wt_sul
peptide count
retention time – overall median
log2 intensity – overall median
Retention time (min)

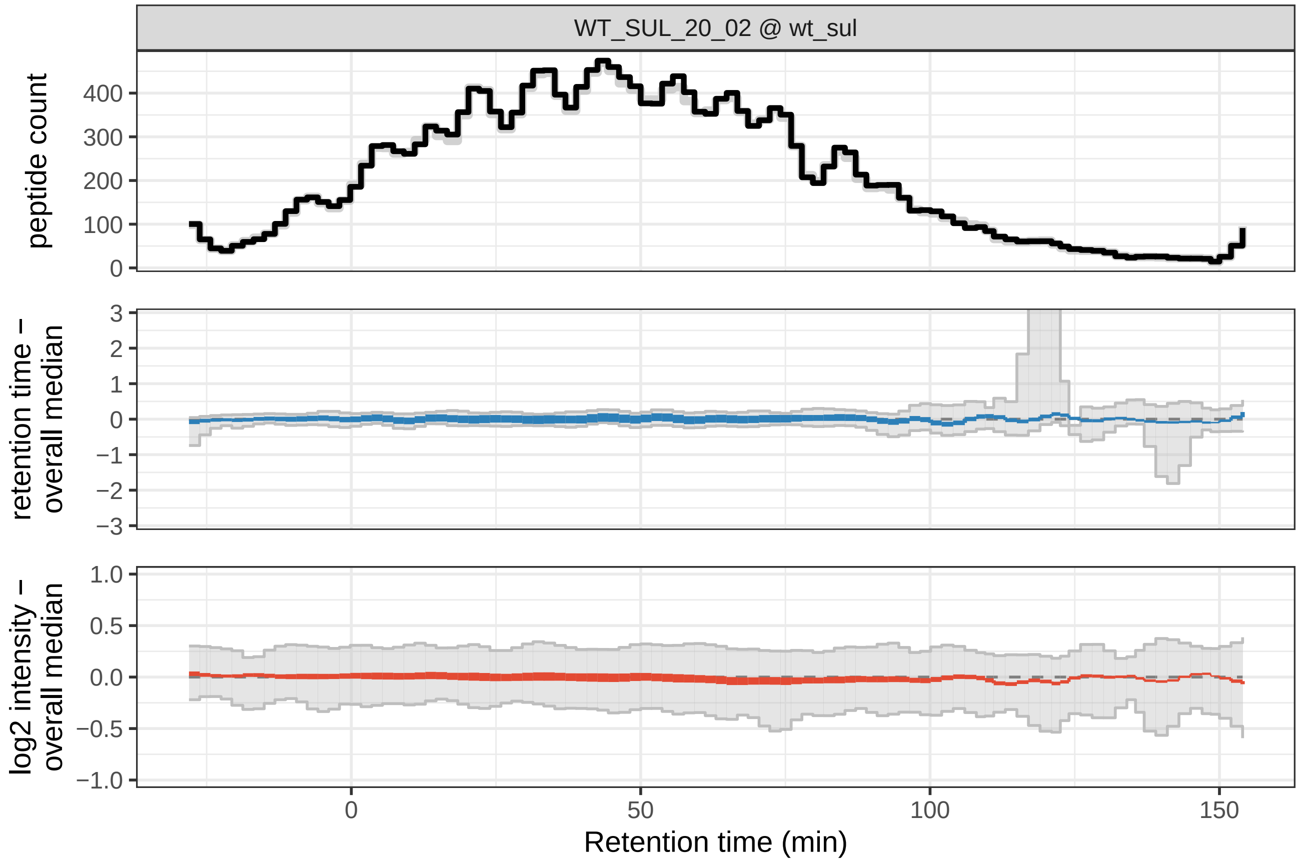
WT_SUL_20_02 @ wt_sul
peptide count
retention time – overall median
log2 intensity – overall median
Retention time (min)

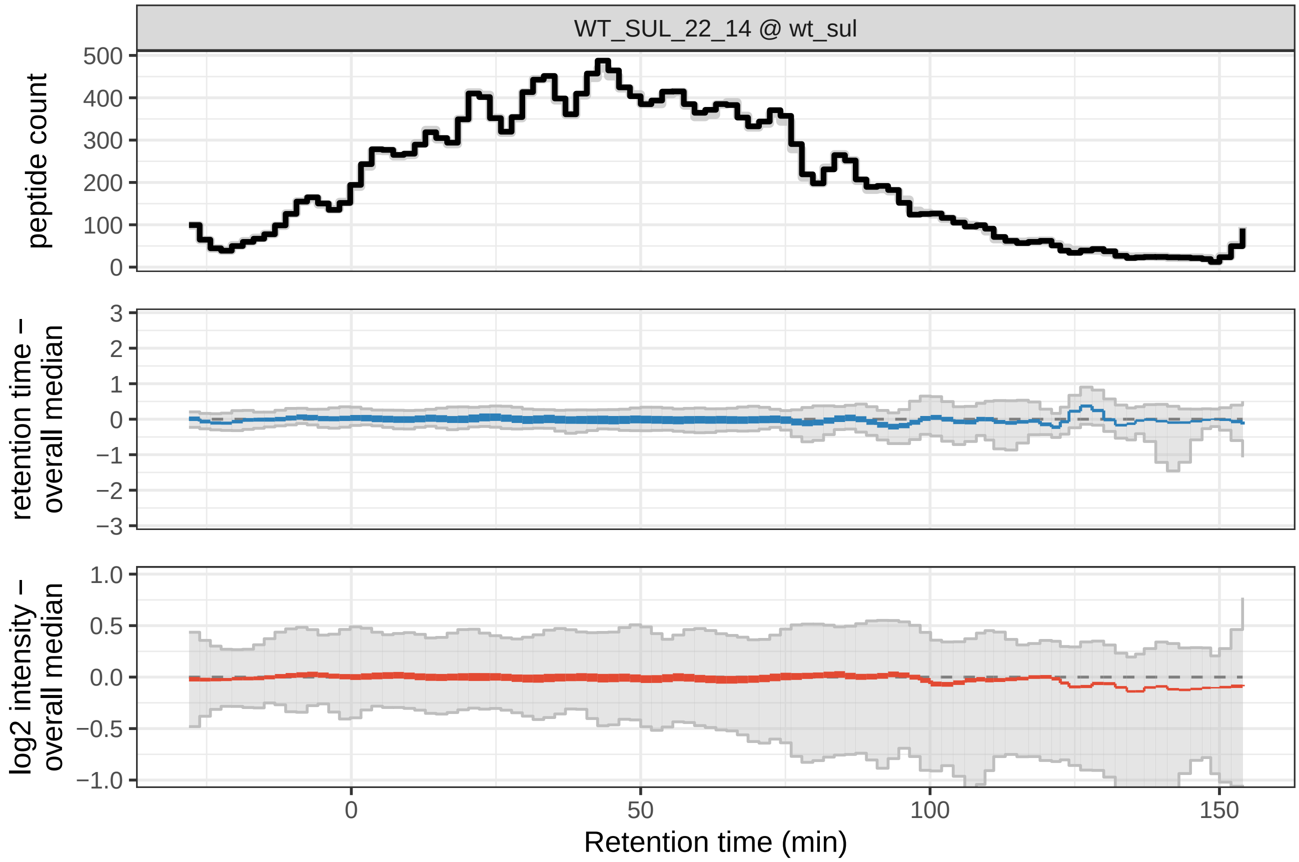
WT_SUL_22_14 @ wt_sul
peptide count
retention time – overall median
log2 intensity – overall median
Retention time (min)

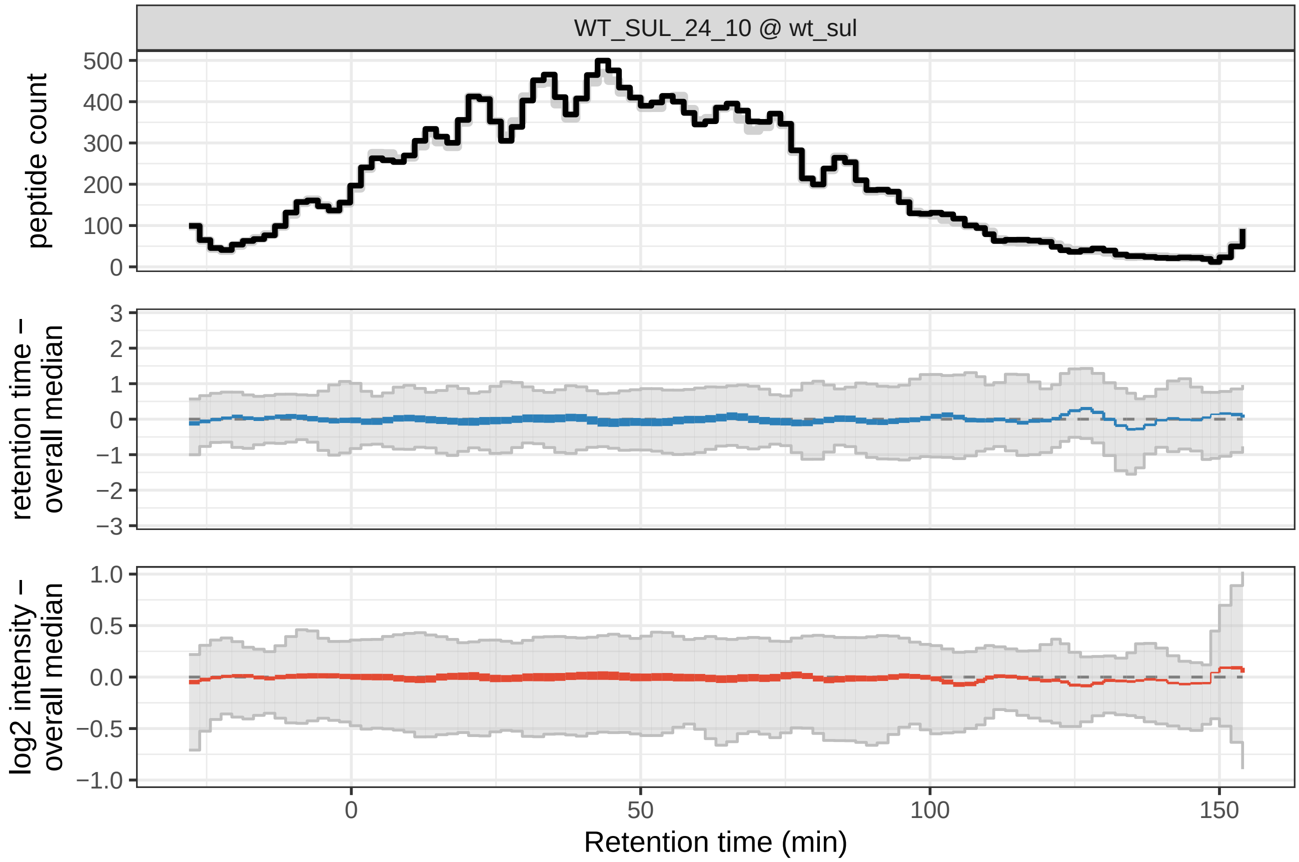
WT_SUL_24_10 @ wt_sul
peptide count
retention time – overall median
log2 intensity – overall median
Retention time (min)

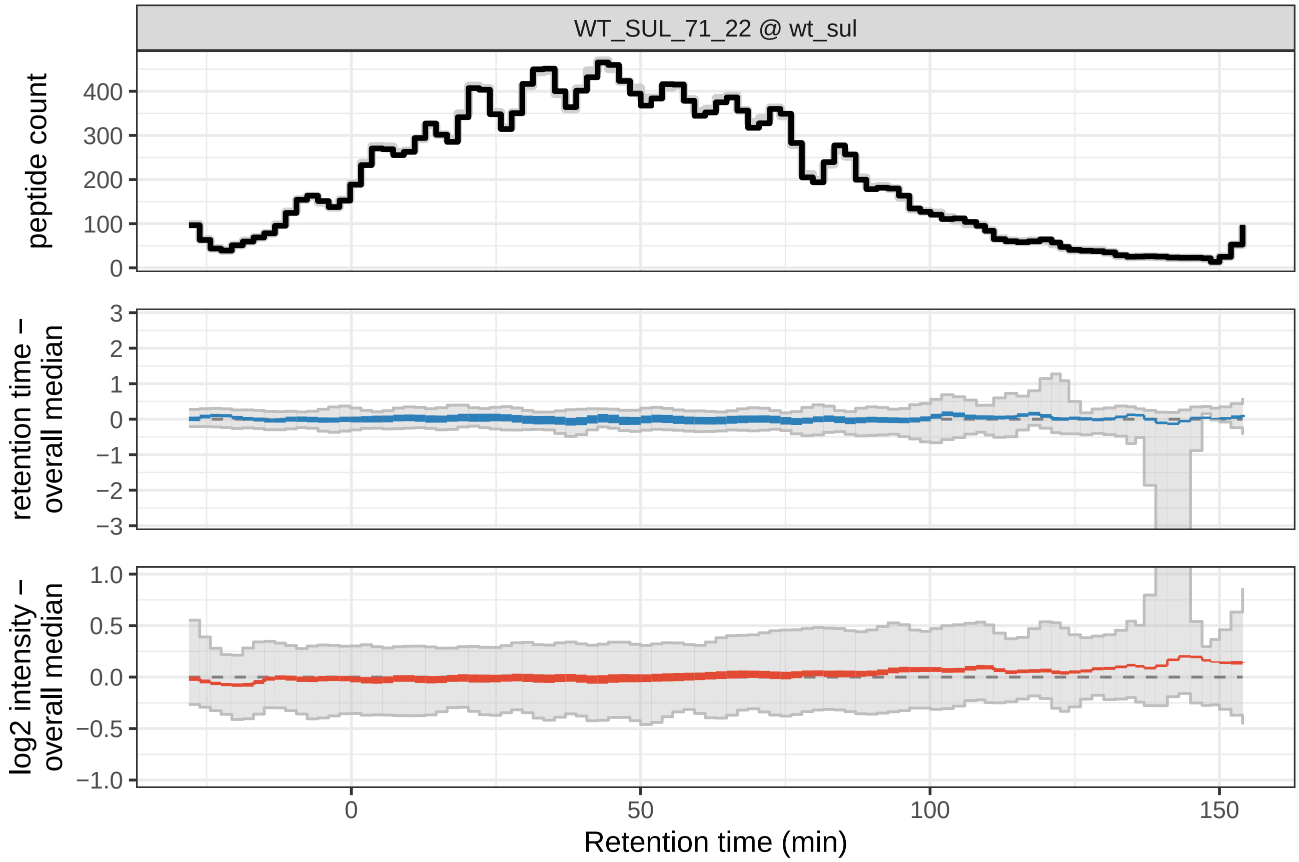
WT_SUL_71_22 @ wt_sul
peptide count
400
300
200
100
0
retention time –
overall median
3
2
1
0
−1
−2
−3
log2 intensity –
overall median
1.0
0.5
0.0
−0.5
−1.0
0
50
100
150
Retention time (min)

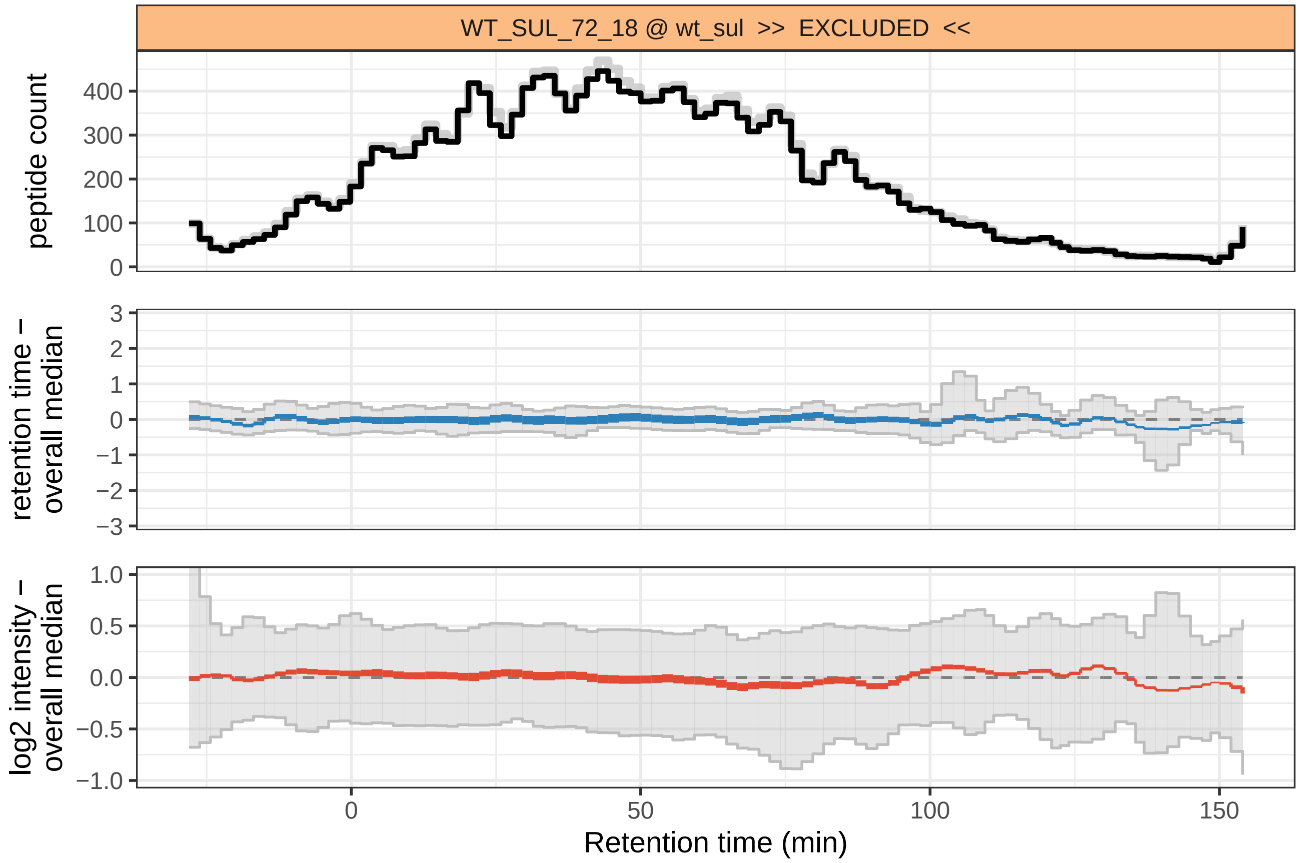
WT_SUL_72_18 @ wt_sul >> EXCLUDED <<
peptide count
400
300
200
100
0
retention time –
overall median
3
2
1
0
−1
−2
−3
log2 intensity –
overall median
1.0
0.5
0.0
−0.5
−1.0
0
50
100
150
Retention time (min)

1.6 variation among replicates

The reproducibility of replicate measurements is expressed in three different analyses. First, the difference between peptide intensities in each sample are compared to the mean value among all replicates (foldchange distributions). Next, the Coefficient of Variation (CoV) is used as a metric for reproducibility to explore how much the CoV within a sample group can be improved by removing a single sample (eg; if CoV strongly improved after removing sample s, it could be regarded as an outlier). Finally, the CoV within each sample group is visualized as a boxplot and a violin plot, figures commonly seen in proteomics literature and useful for comparing across experiments (of similar protocol).

1.6.1 within-group foldchange distributions

The foldchange of all peptides in a sample is compared to their respective mean value over all samples in the group. This visualizes how strongly each sample deviates from other samples in the same group which helps identify outlier samples. The same data was used as detailed in the "retention time" section above. Samples marked as 'exclude' in the provided sample metadata table are visualized as dashed lines.

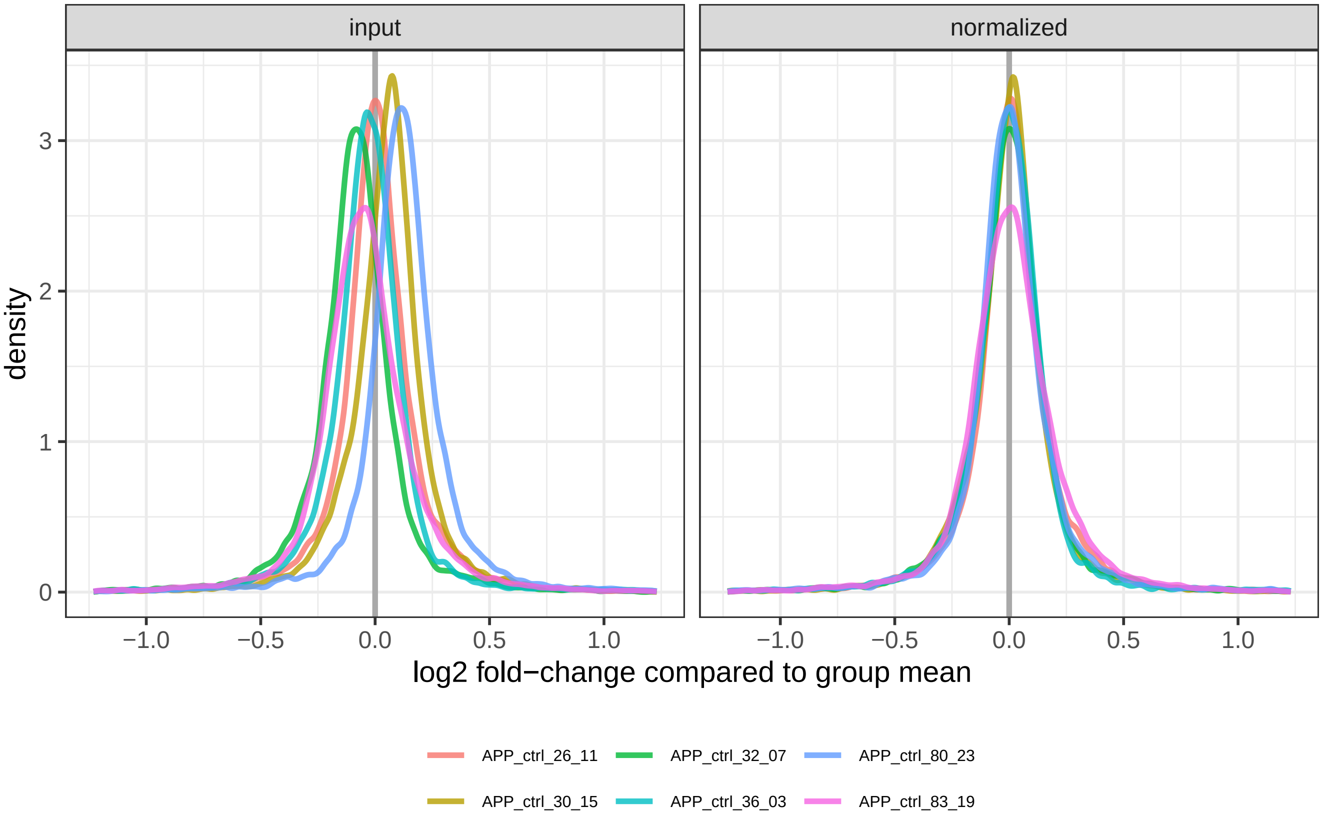

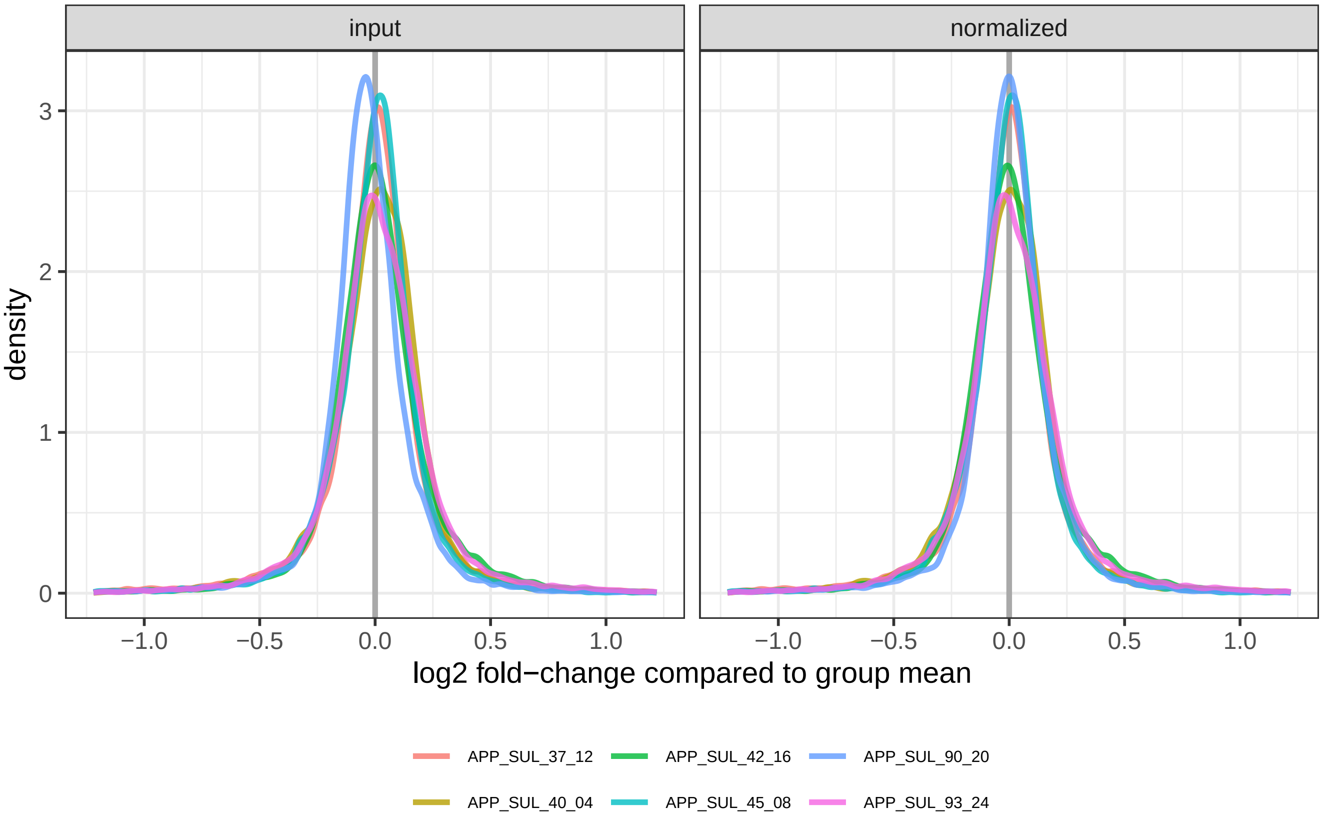
input
normalized
density
3
2
1
0
−1.0
−0.5
0.0
0.5
1.0
log2 fold−change compared to group mean
APP_SUL_37_12
APP_SUL_42_16
APP_SUL_90_20
APP_SUL_40_04
APP_SUL_45_08
APP_SUL_93_24

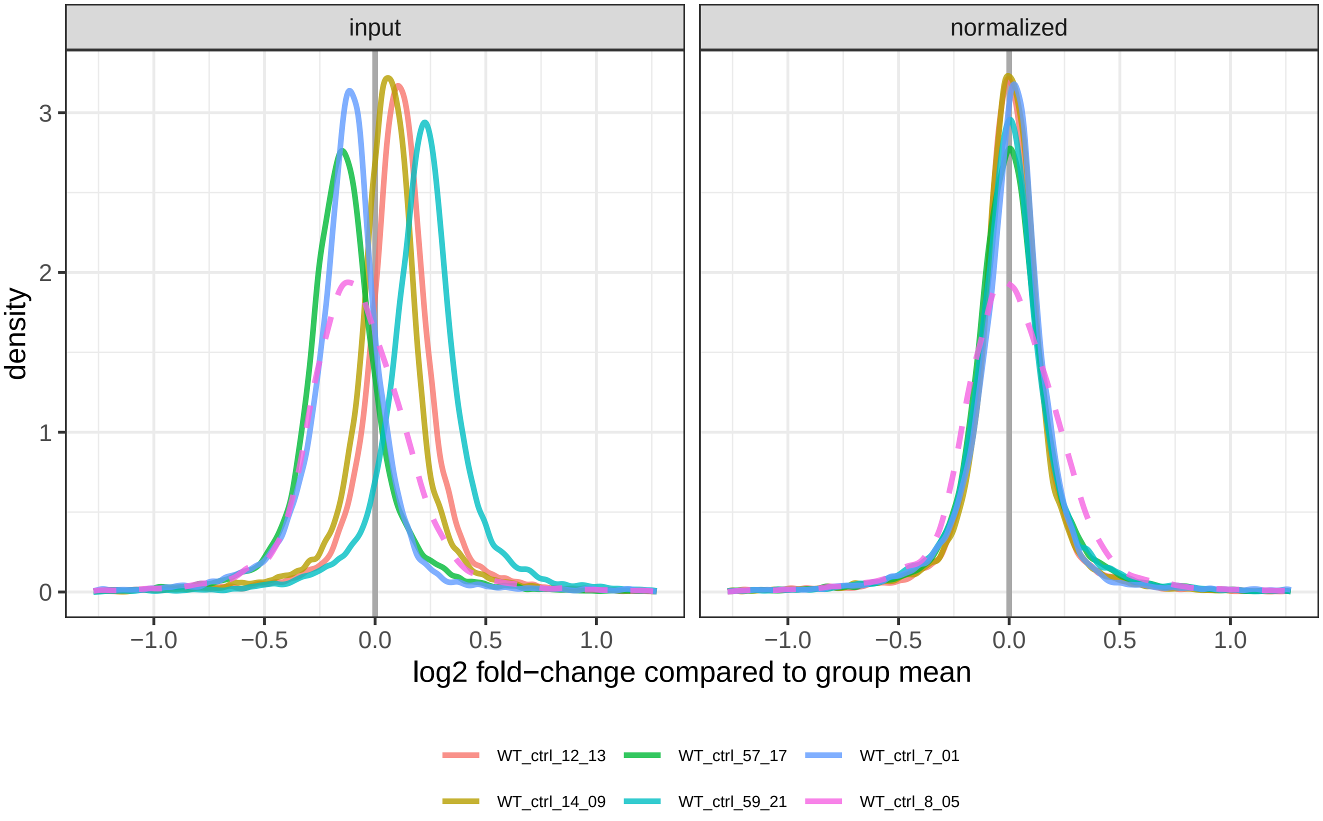
input
normalized
density
3
2
1
0
−1.0
−0.5
0.0
0.5
1.0
log2 fold−change compared to group mean
WT_ctrl_12_13
WT_ctrl_57_17
WT_ctrl_7_01
WT_ctrl_14_09
WT_ctrl_59_21
WT_ctrl_8_05

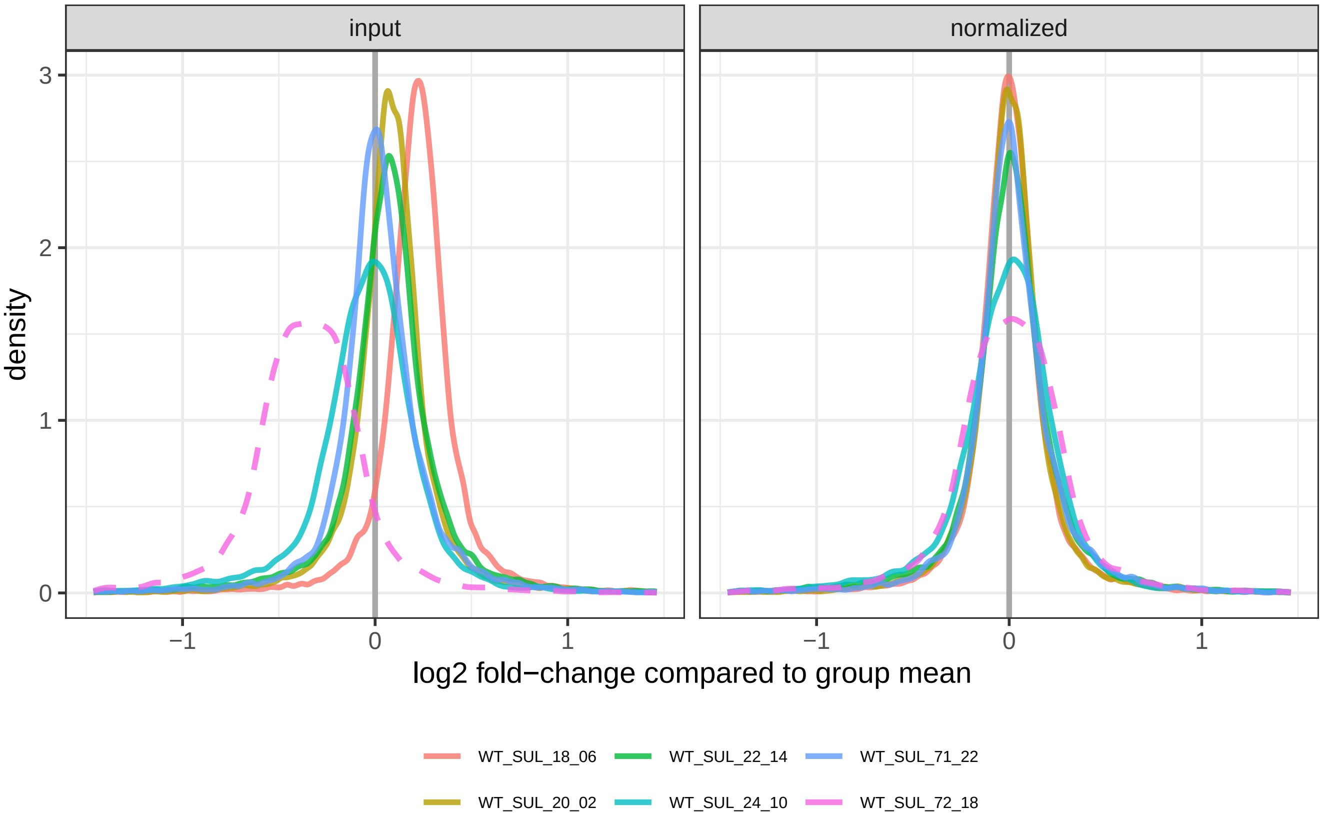

input
normalized
3
2
1
0
density
−1
0
1
log2 fold−change compared to group mean
WT_SUL_18_06
WT_SUL_22_14
WT_SUL_71_22
WT_SUL_20_02
WT_SUL_24_10
WT_SUL_72_18

1.6.2 CoV, leave-one-out

progress: leave-one-out CoV plot computations took 14 seconds The figures below describe the effect of removing a particular sample prior to within-group Coefficient of Variation (CoV) computation. The lower the CoV distribution is for a sample, the better reproducibility we get by excluding it. Only sample groups with at least 4 replicates can be used for this analysis, so 3 samples remain after leaving one out. Samples marked as 'exclude' in the provided sample metadata are included in these analyses (shown as dashed lines), and only peptides with at least 3 data points across replicates samples (after leave-one-out) are used for each CoV computation.

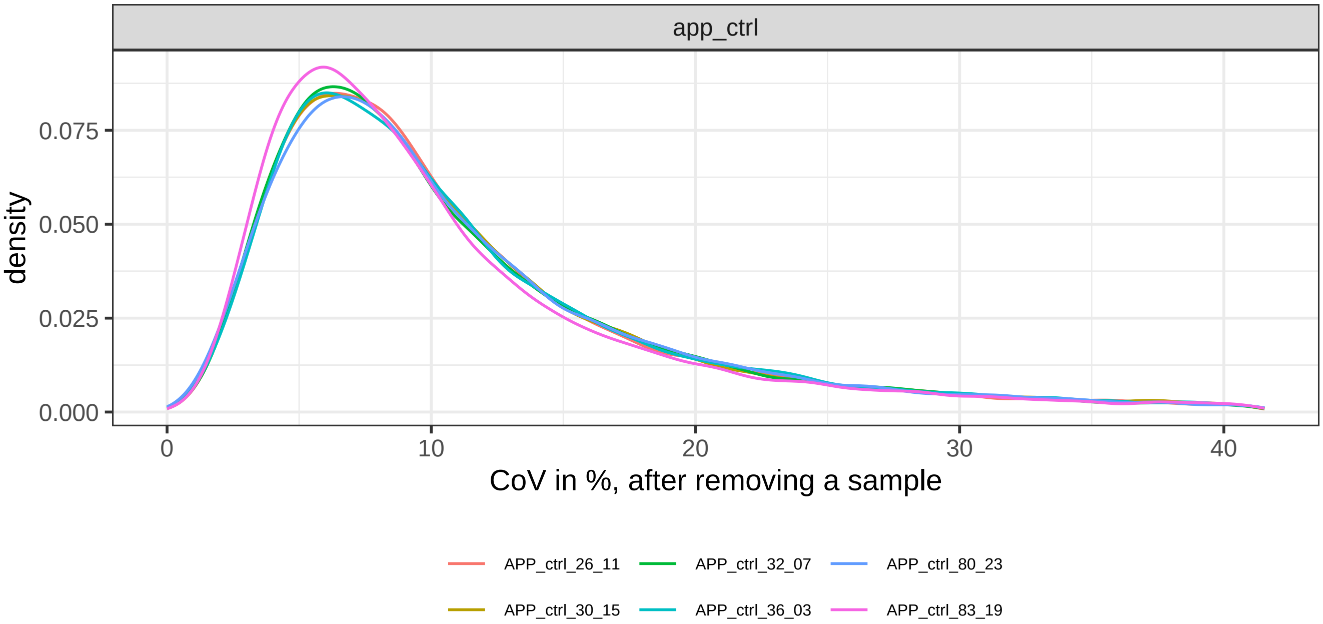

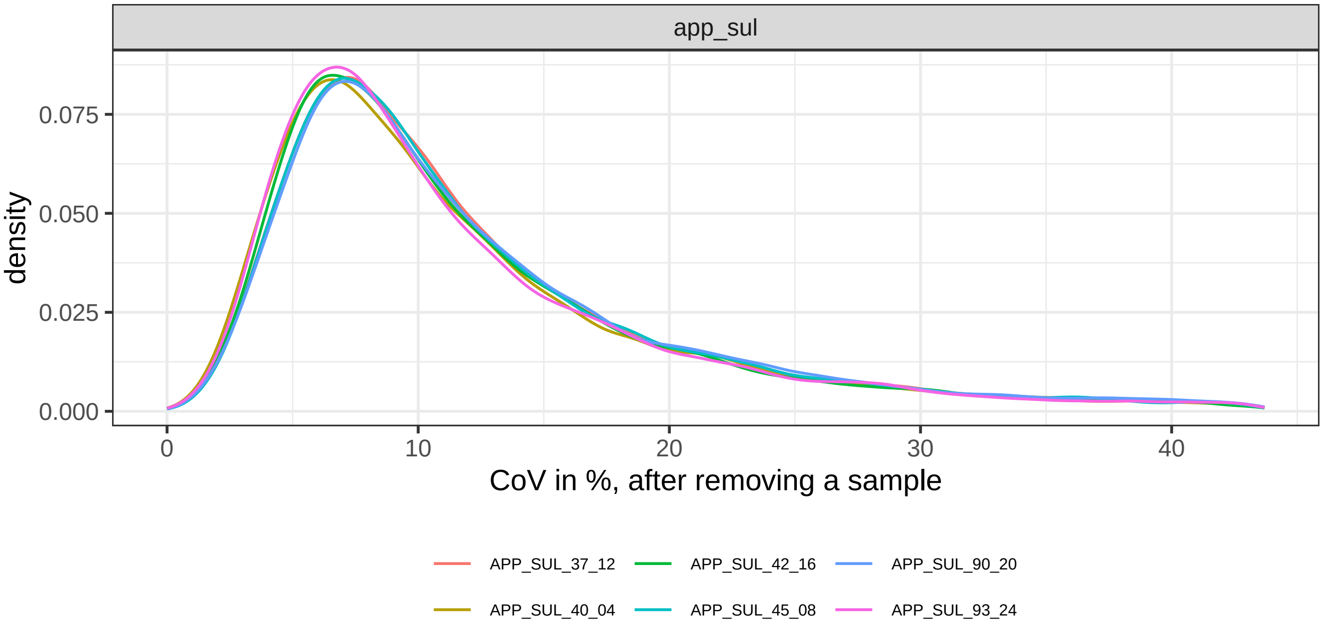

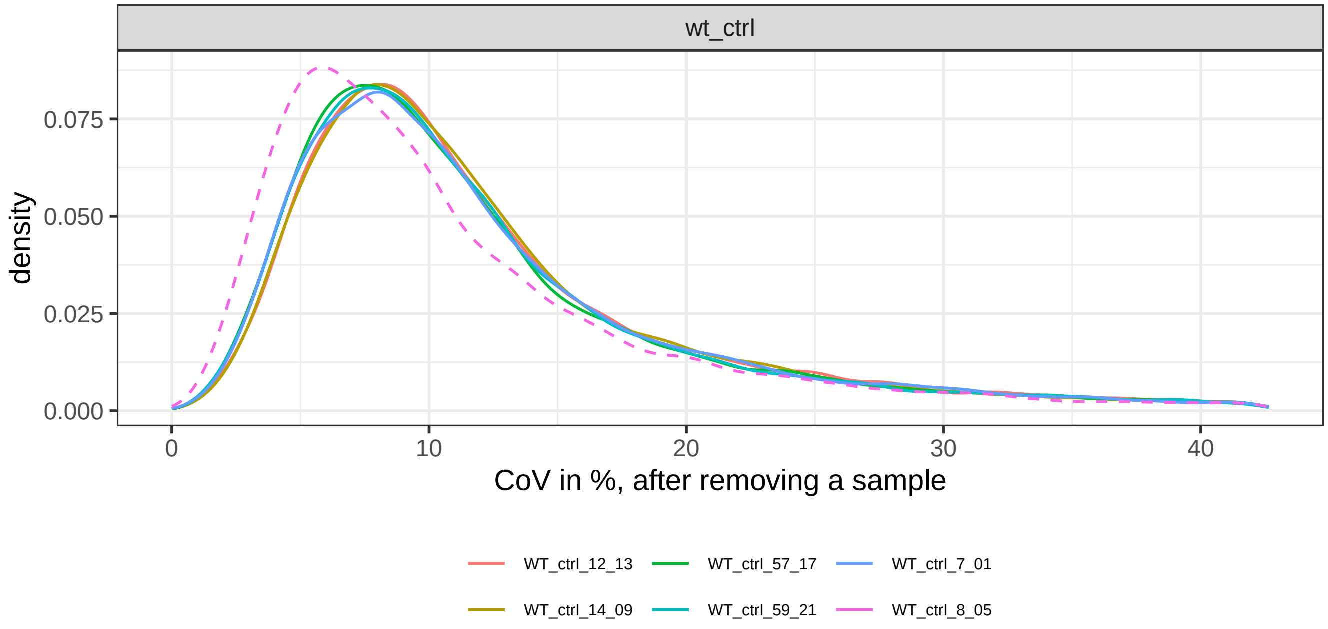
wt_ctrl
density
0.000
0.025
0.050
0.075
0
10
20
30
40
CoV in %, after removing a sample
WT_ctrl_12_13
WT_ctrl_57_17
WT_ctrl_7_01
WT_ctrl_14_09
WT_ctrl_59_21
WT_ctrl_8_05

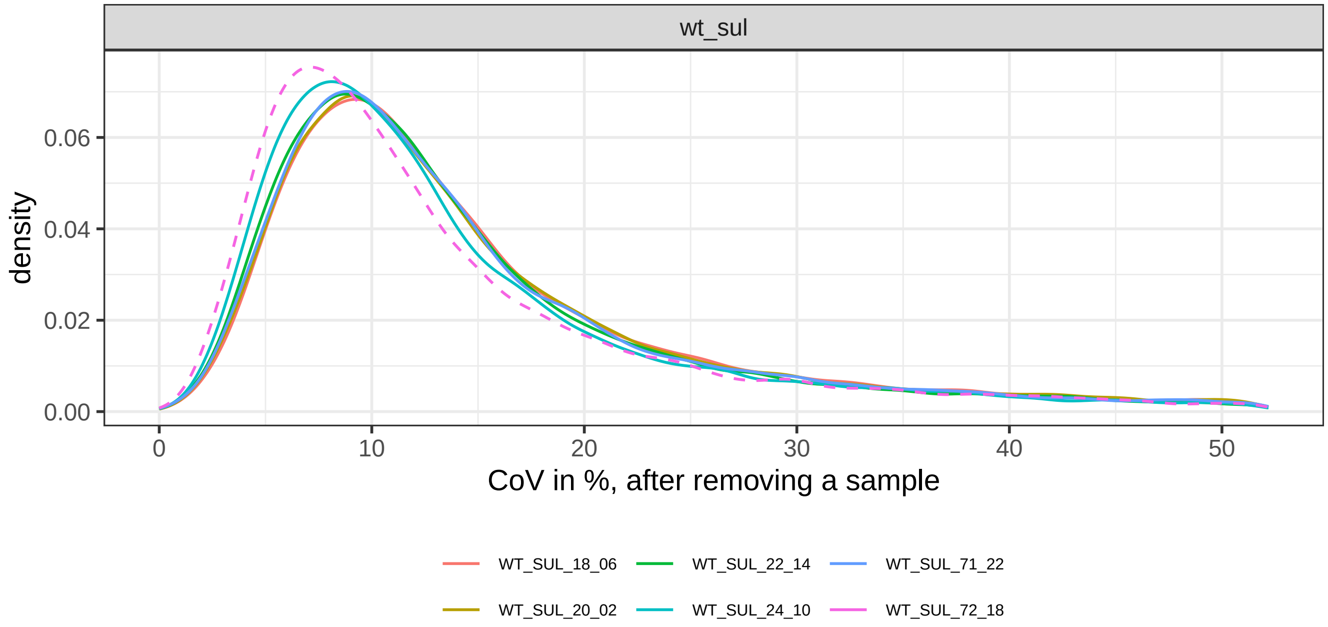
wt_sul
density
0.00
0.02
0.04
0.06
0
10
20
30
40
50
CoV in %, after removing a sample
WT_SUL_18_06
WT_SUL_22_14
WT_SUL_71_22
WT_SUL_20_02
WT_SUL_24_10
WT_SUL_72_18

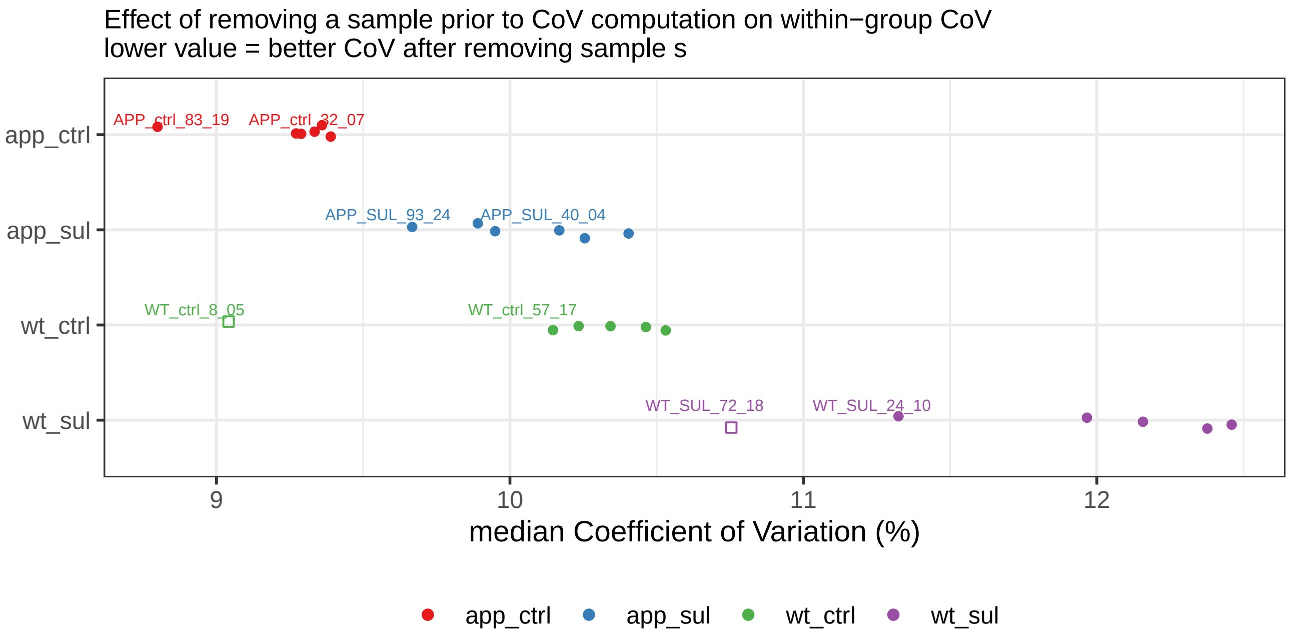
Effect of removing a sample prior to CoV computation on within−group CoV
lower value = better CoV after removing sample s
app_ctrl
app_sul
wt_ctrl
wt_sul
APP_ctrl_83_19
APP_ctrl_32_07
APP_SUL_93_24
APP_SUL_40_04
WT_ctrl_8_05
WT_ctrl_57_17
WT_SUL_72_18
WT_SUL_24_10
9
10
11
12
median Coefficient of Variation (%)
app_ctrl
app_sul
wt_ctrl
wt_sul

shortname	group	exclude	median CoV
APP_ctrl_83_19	app_ctrl	FALSE	8.8
APP_ctrl_32_07	app_ctrl	FALSE	9.3
APP_ctrl_26_11	app_ctrl	FALSE	9.3
APP_ctrl_30_15	app_ctrl	FALSE	9.3
APP_ctrl_80_23	app_ctrl	FALSE	9.4
APP_ctrl_36_03	app_ctrl	FALSE	9.4
APP_SUL_93_24	app_sul	FALSE	9.7
APP_SUL_40_04	app_sul	FALSE	9.9
APP_SUL_42_16	app_sul	FALSE	9.9
APP_SUL_37_12	app_sul	FALSE	10.2
APP_SUL_45_08	app_sul	FALSE	10.3
APP_SUL_90_20	app_sul	FALSE	10.4
WT_ctrl_8_05	wt_ctrl	TRUE	9.0
WT_ctrl_57_17	wt_ctrl	FALSE	10.1
WT_ctrl_59_21	wt_ctrl	FALSE	10.2
WT_ctrl_7_01	wt_ctrl	FALSE	10.3
WT_ctrl_12_13	wt_ctrl	FALSE	10.5
WT_ctrl_14_09	wt_ctrl	FALSE	10.5
WT_SUL_72_18	wt_sul	TRUE	10.8
WT_SUL_24_10	wt_sul	FALSE	11.3
WT_SUL_22_14	wt_sul	FALSE	12.0
WT_SUL_71_22	wt_sul	FALSE	12.2
WT_SUL_20_02	wt_sul	FALSE	12.4
WT_SUL_18_06	wt_sul	FALSE	12.5

Leave-one-out impact on within-group CoV (%)

1.6.3 Coefficient of Variation

The Coefficient of Variation (CoV) is a quality metric for the reproducibility of replicate measurements, here visualized using box- and violin-plots.

The same data was used as detailed in the "retention time" section above, with only peptides quantified in at least 3 replicates used for CoV computation in a group. Only samples that are NOT marked 'exclude' in the provided sample metadata and are in a sample group among at least 3 replicates are used for these figures.

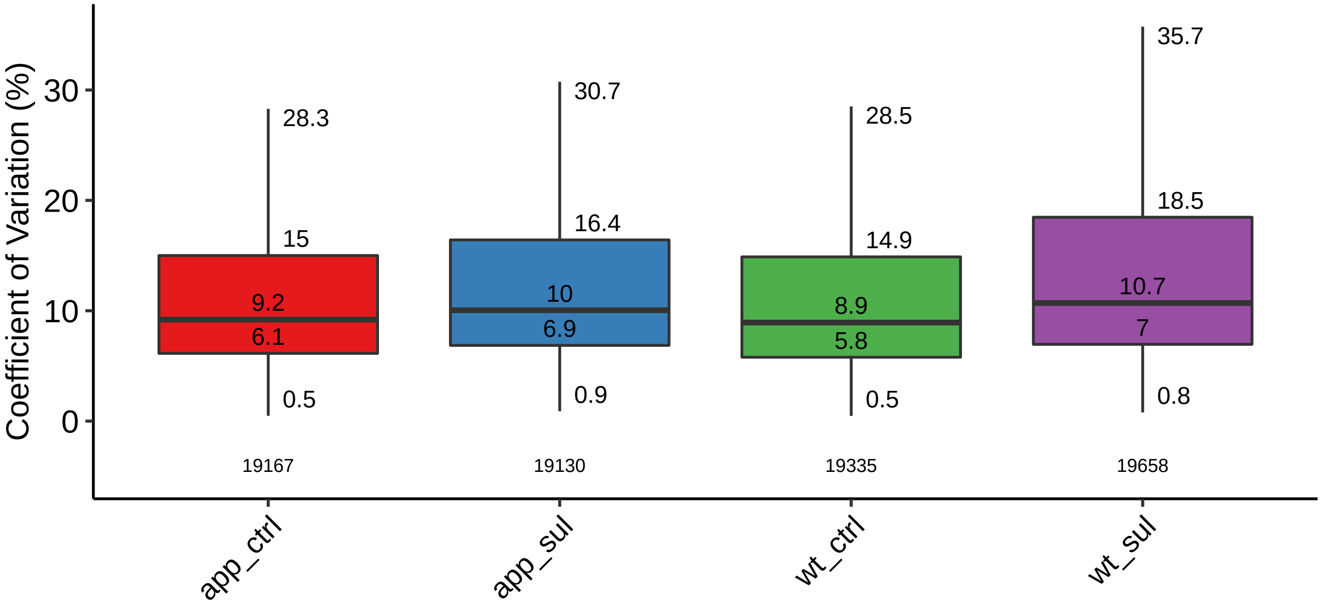

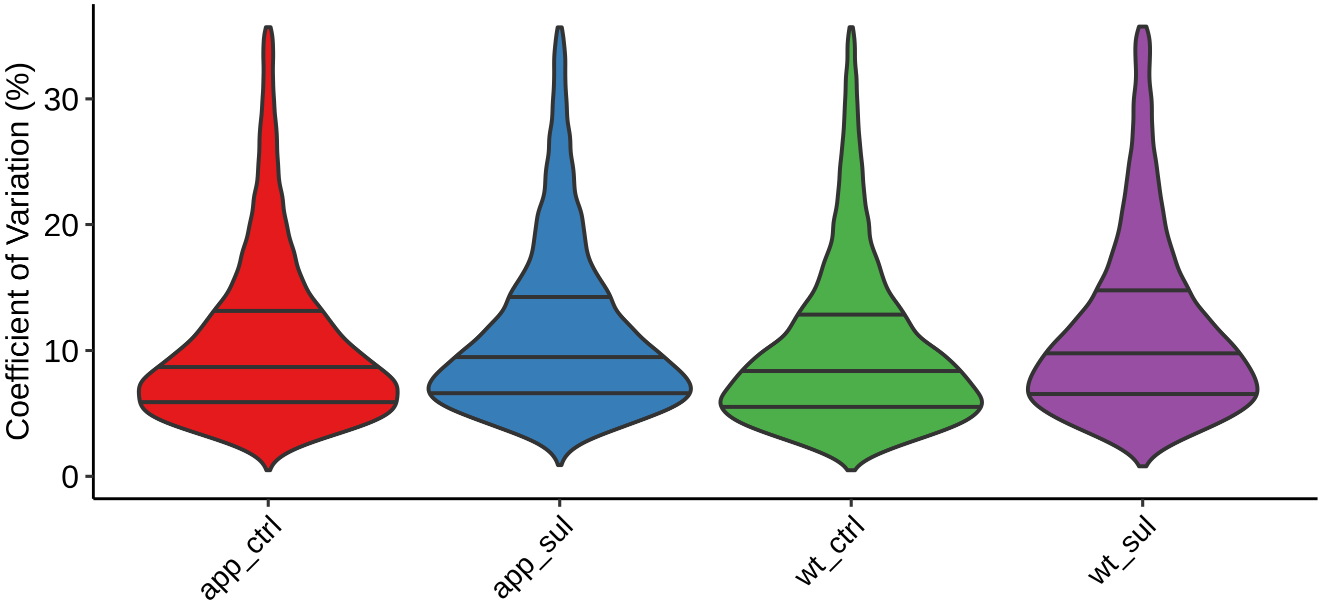

1.7 PCA

A visualization of the first three PCA dimensions illustrates sample clustering. The goal of these figures is to detect global effects from a quality control perspective, such as samples from the same experiment batch clustering together, not to be sensitive to a minor subset of differentially abundant proteins (for which specialized statistical models can be applied downstream).

If additional sample metadata was provided, such as experiment batch, sample-prep dates, gel, etc., multiple PCA figures will be generated with respective color-codings. Users are encouraged to provide relevant experiment information as sample metadata and use these figures to search for unexpected batch effects.

The pcaMethods R package is used here to perform the Probabilistic PCA (PPCA). The set of peptides used for this analysis consists of those peptides that pass your filter criteria in every sample group. If any samples are marked as 'exclude' in the provided sample metadata, an additional PCA plot is generated with these samples included (depicting the 'exclude' samples as square symbols).

Rationale behind data filter
As mentioned above, the aim of the PCA figures is to identify global effects. To achieve this, we compute sample distances on the subset of peptides identified in each group which prevents rarely detected peptides/proteins from having a disproportionate effect on sample clustering. This pertains not only to 'randomly detected contaminant proteins' but also to proteins with abundance levels near the detection limit, which may be detected in only a subset of samples (eg; some measurements will be more successful/sensitive than others).

Figure legends
The first 3 principle components compared visually (1 *vs* 2, 1 *vs* 3, 2 *vs* 3) on the rows. Left- and right-side panels on each row represent the same figure without and with sample labels. The principle components are shown on the axis labels together with their respective percentage of variance explained. Samples marked as 'exclude' in the provided sample metadata, if any, are visualized as square shapes.

PCA of all samples, including those flagged as 'exclude', using 17452 peptides

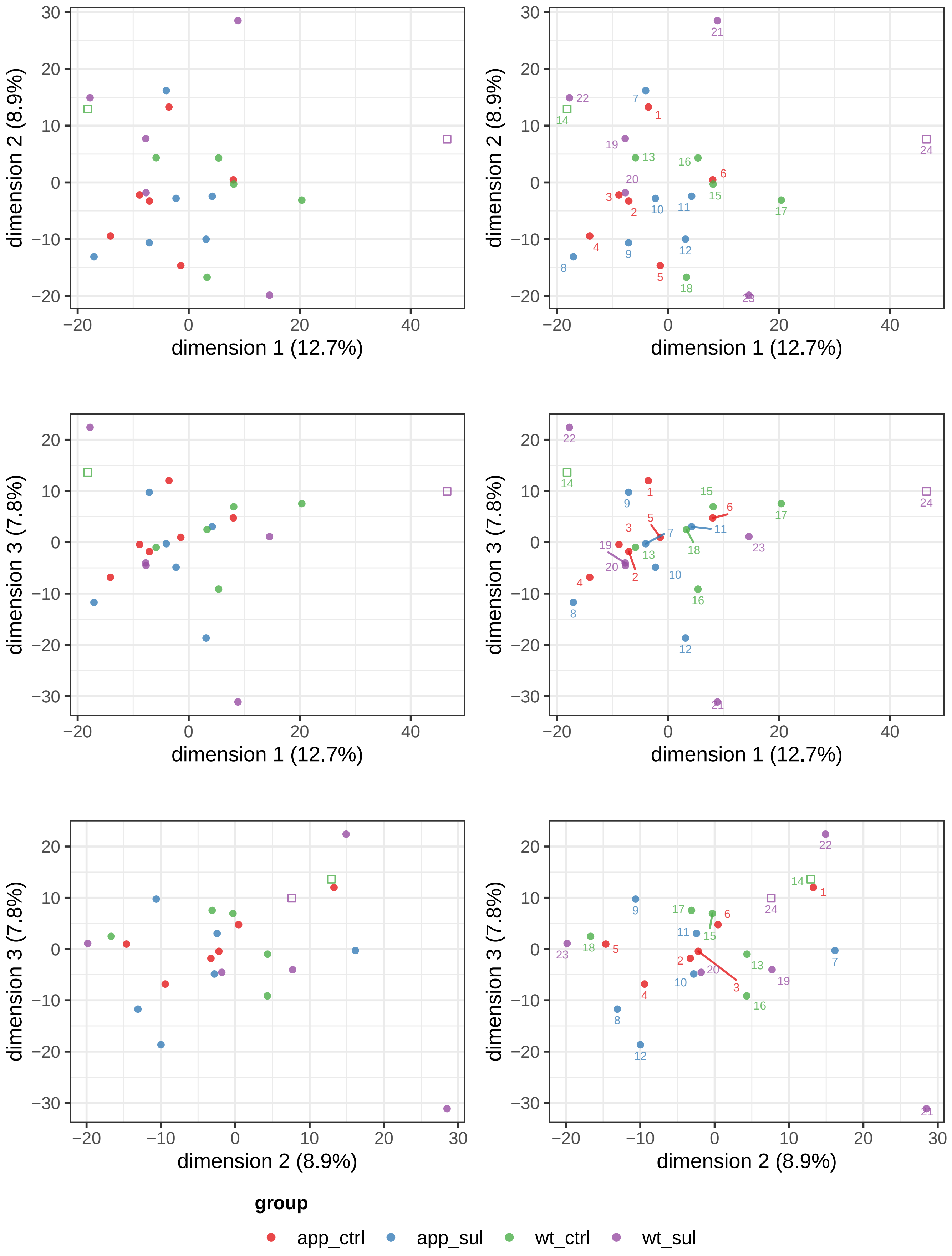

PCA only on samples not flagged as ‘exclude’, using 17695 peptides

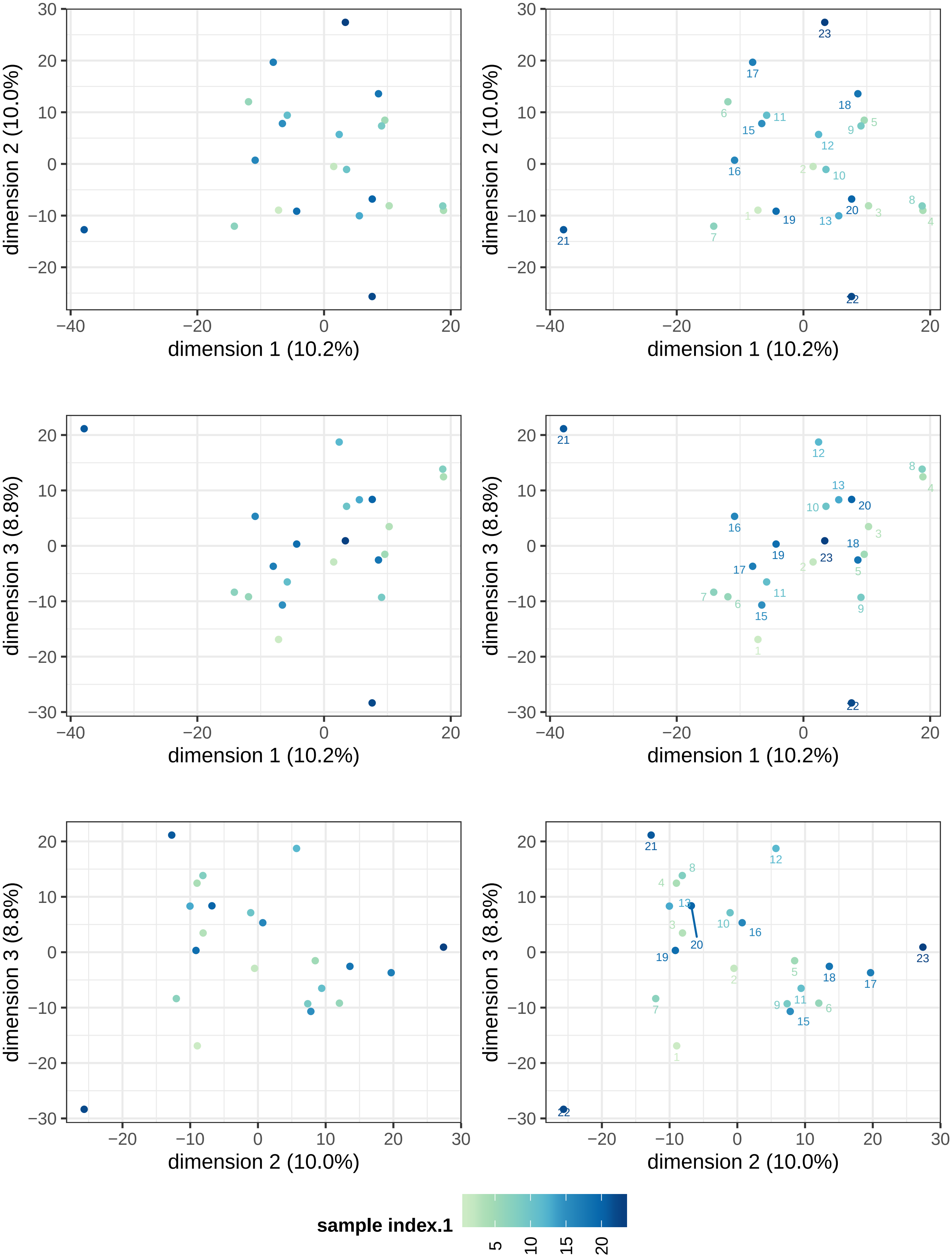

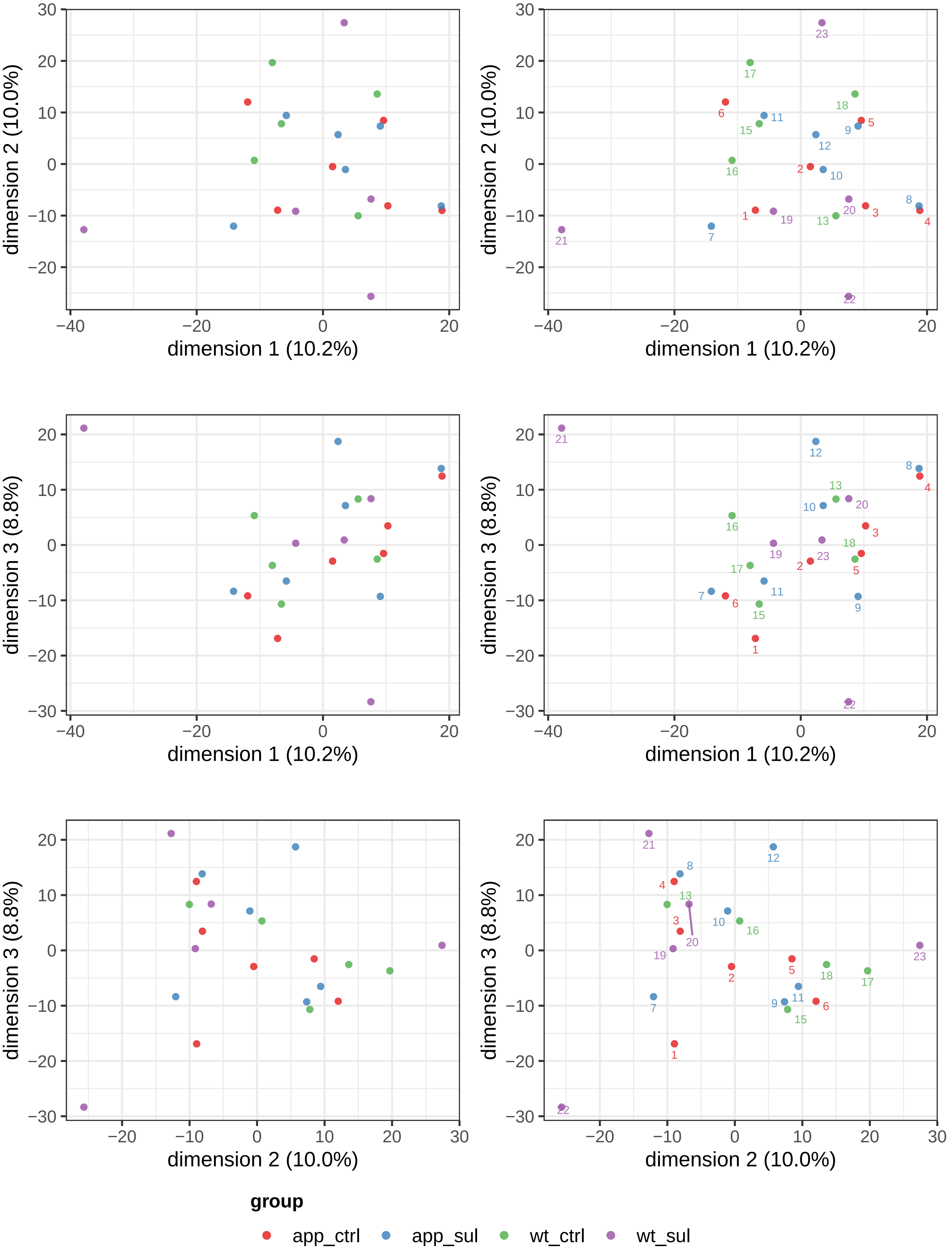
dimension 1 (10.2%)
dimension 2 (10.0%)
dimension 3 (8.8%)
group
app_ctrl
app_sul
wt_ctrl
wt_sul

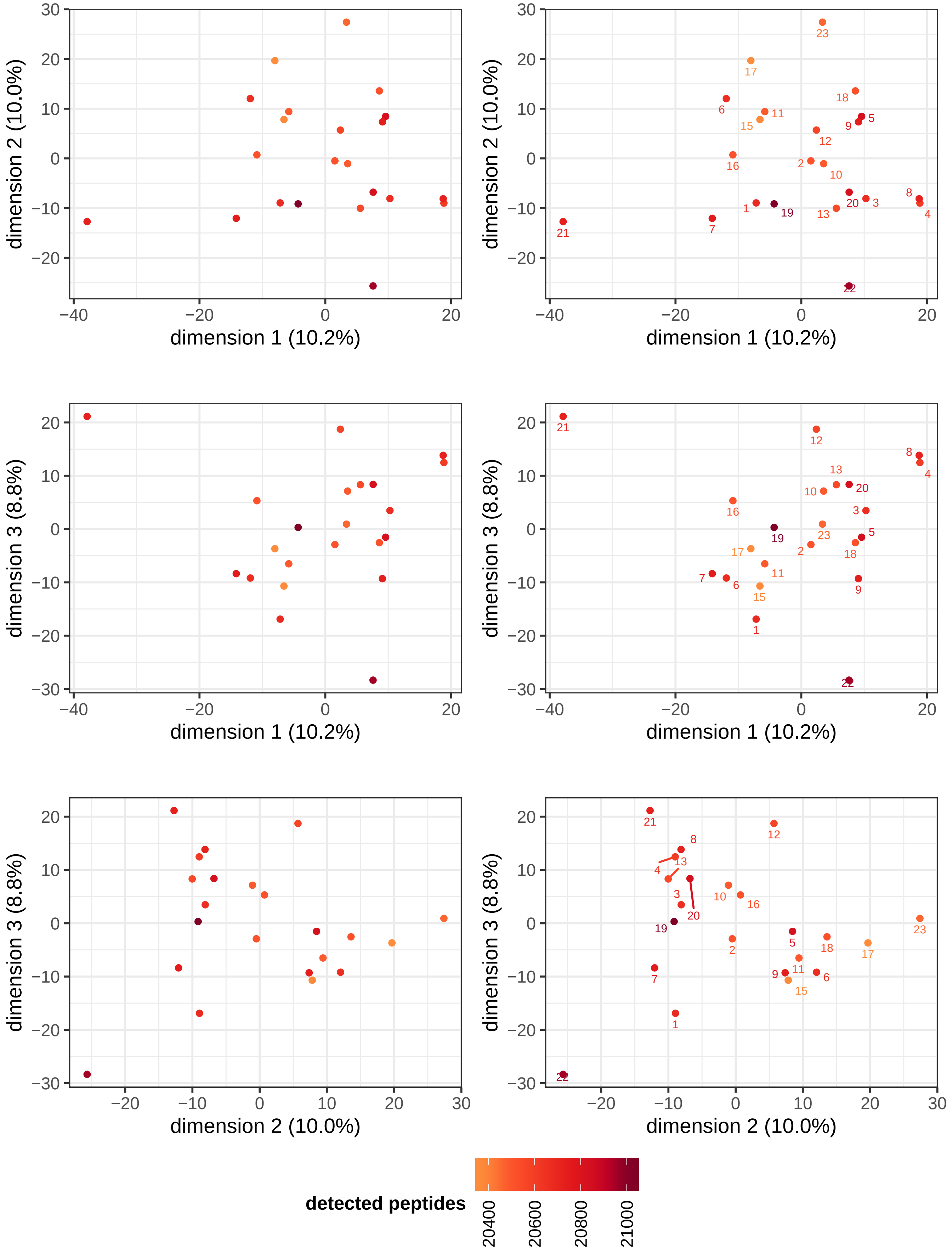

dimension 1 (10.2%)
dimension 2 (10.0%)
dimension 3 (8.8%)
detected peptides
20400
20600
20800
21000

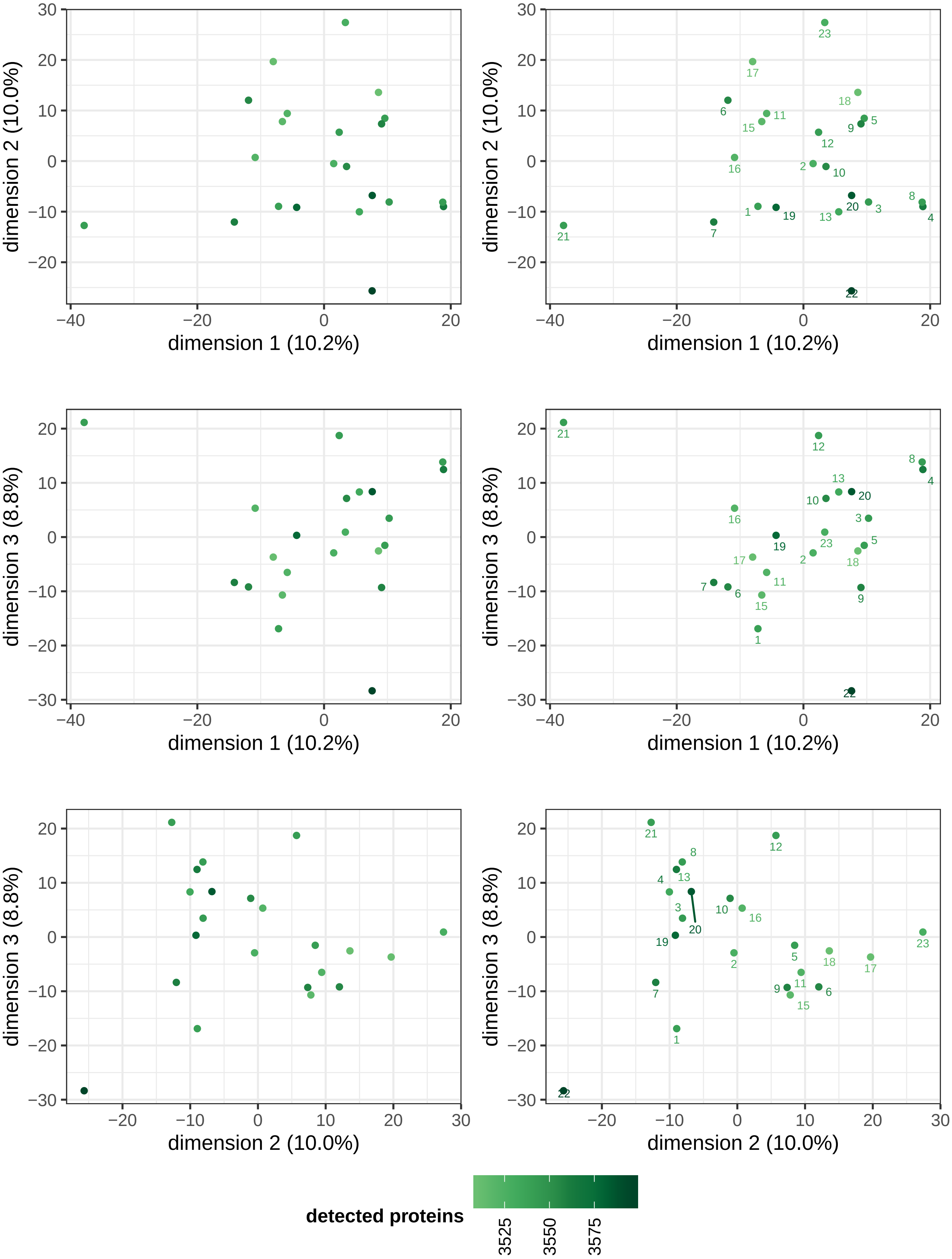
dimension 1 (10.2%)
dimension 2 (10.0%)
dimension 3 (8.8%)
detected proteins
3525
3550
3575

2 Differential abundance analysis

goal: maximize reliable features for quantification

In a pairwise analysis of two groups of samples, only peptides with N data-points in both groups are used for quantitative analysis (where N = defined by user settings). For example; if peptide *p* is consistently quantified in sample groups A and B but not in C/D/E, it can be used when comparing group A *versus* group B but should not be used in any other group comparisons. This approach is particularly suited to maximize the number of peptides used for statistical analysis in experimental designs with many sample groups.

A common alternative strategy is a global filtering approach where peptides are selected based on their properties in the overall dataset (eg; present in x% of samples or x% of replicates in all groups) and subsequentially the resulting data matrix is used for all downstream statistical analyses. In the example above where peptide *p* is present in a subset of sample groups, *p* would either be left out (not present in majority of samples in entire dataset) or erronously used when applying t-statistics to groups B and C (since *p* is not present in group C, it may differentially detected but there are no features available for quantitative analysis)

2.1 wt_ctrl vs wt_sul

- **user setting:** using 'filter by contrast' peptide filtering approach
- 24511 peptides in 3865 proteins remain in the current contrast after peptide filters and are used for the statistical analysis in this section
- qvalue threshold: 0.05
- log2 foldchange threshold: 0.2201748

2.1.1 volcano

The plot title shows the statistical model and contrast (sample groups in the comparison). Left- and right-side figure panels on each row represent the same figure without and with labels for the 25 proteins with lowest p-value.

Bottom figure panels have limited x- and y-axis. For datasets with a small number of strong outliers in p-value or fold-change, which may have a profound effect on the plot scales, this allows inspection of the remainder of the volcano plot without disproportionate influence by 'extreme' values.

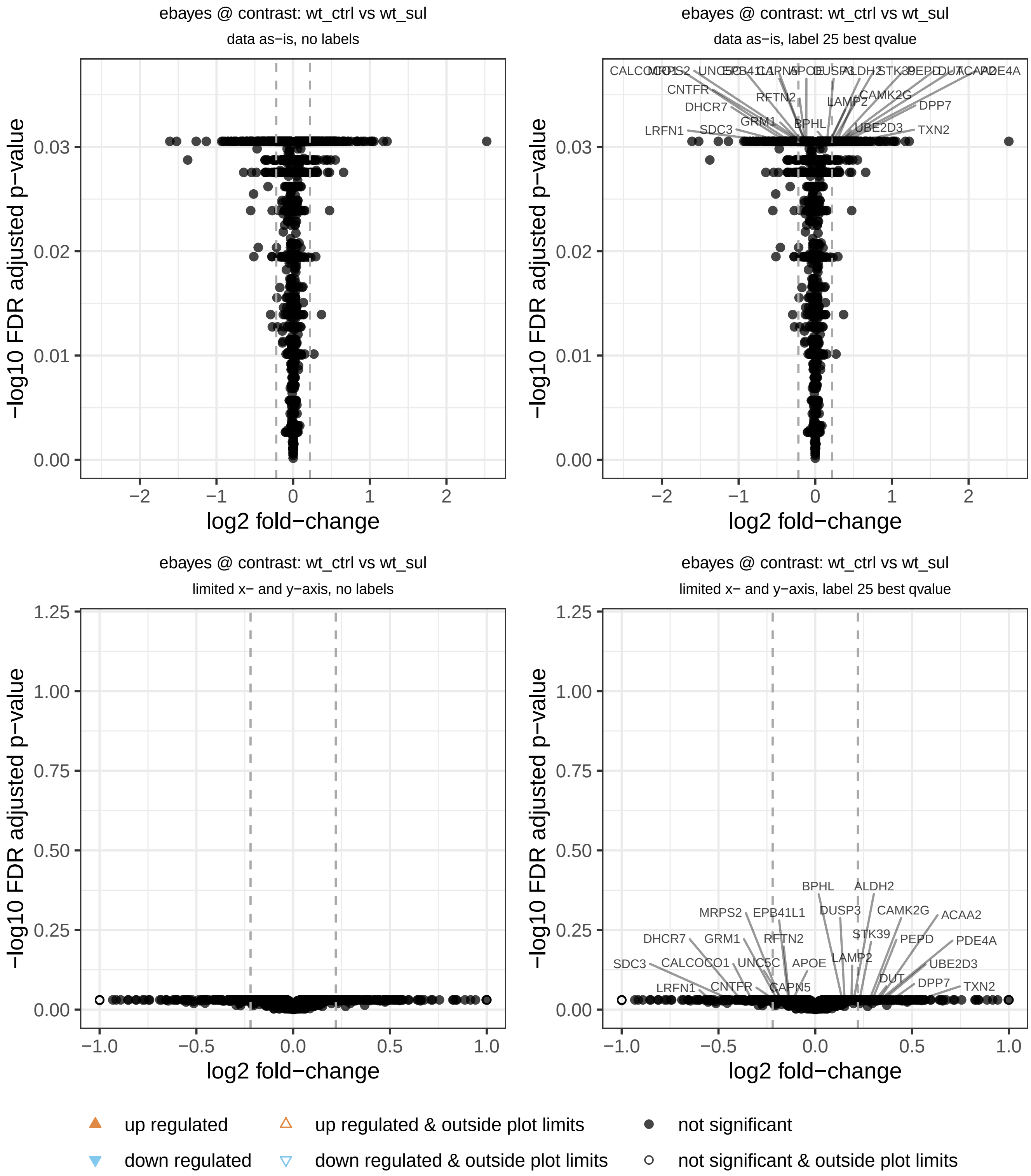
ebayes @ contrast: wt_ctrl vs wt_sul
data as−is, no labels
ebayes @ contrast: wt_ctrl vs wt_sul
data as−is, label 25 best qvalue
ebayes @ contrast: wt_ctrl vs wt_sul
limited x− and y−axis, no labels
ebayes @ contrast: wt_ctrl vs wt_sul
limited x− and y−axis, label 25 best qvalue
−log10 FDR adjusted p−value
log2 fold−change
BPHL
ALDH2
MRPS2
EPB41L1
DUSP3
CAMK2G
ACAA2
DHCR7
GRM1
RFTN2
STK39
PEPD
PDE4A
SDC3
CALCOCO1
UNC5C
APOE
LAMP2
UBE2D3
DUT
DPP7
TXN2
LRFN1
CNTFR
CAPN5
up regulated
up regulated & outside plot limits
not significant
down regulated
down regulated & outside plot limits
not significant & outside plot limits

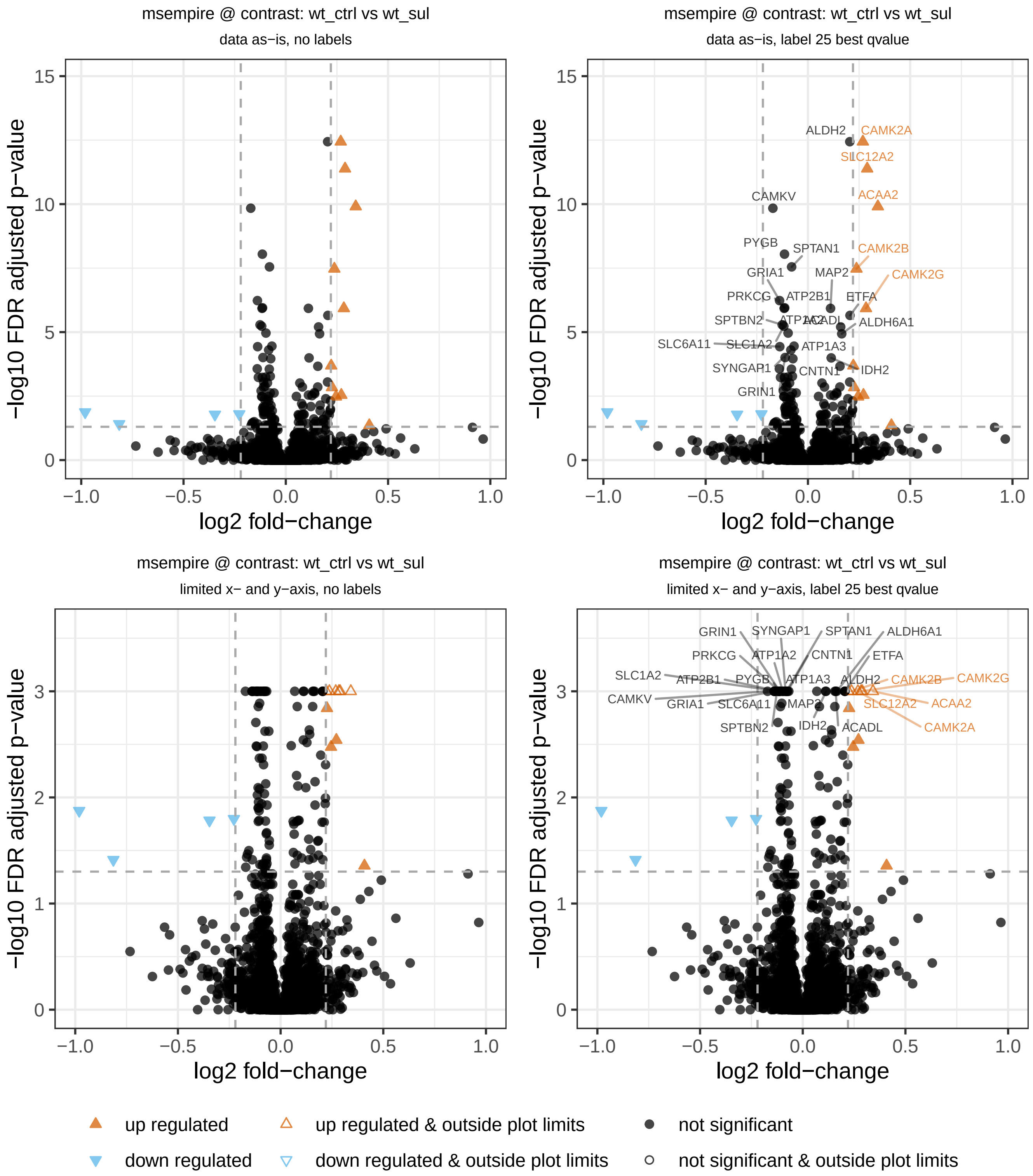
msempire @ contrast: wt_ctrl vs wt_sul
data as−is, no labels
msempire @ contrast: wt_ctrl vs wt_sul
data as−is, label 25 best qvalue
ALDH2
CAMK2A
SLC12A2
CAMKV
ACAA2
PYGB
SPTAN1
CAMK2B
GRIA1
MAP2
CAMK2G
PRKCG
ATP2B1
ETFA
SPTBN2
ATP1A2
ACADL
ALDH6A1
SLC6A11
SLC1A2
ATP1A3
SYNGAP1
CNTN1
IDH2
GRIN1
msempire @ contrast: wt_ctrl vs wt_sul
limited x− and y−axis, no labels
msempire @ contrast: wt_ctrl vs wt_sul
limited x− and y−axis, label 25 best qvalue
−log10 FDR adjusted p−value
log2 fold−change
up regulated
up regulated & outside plot limits
not significant
down regulated
down regulated & outside plot limits
not significant & outside plot limits

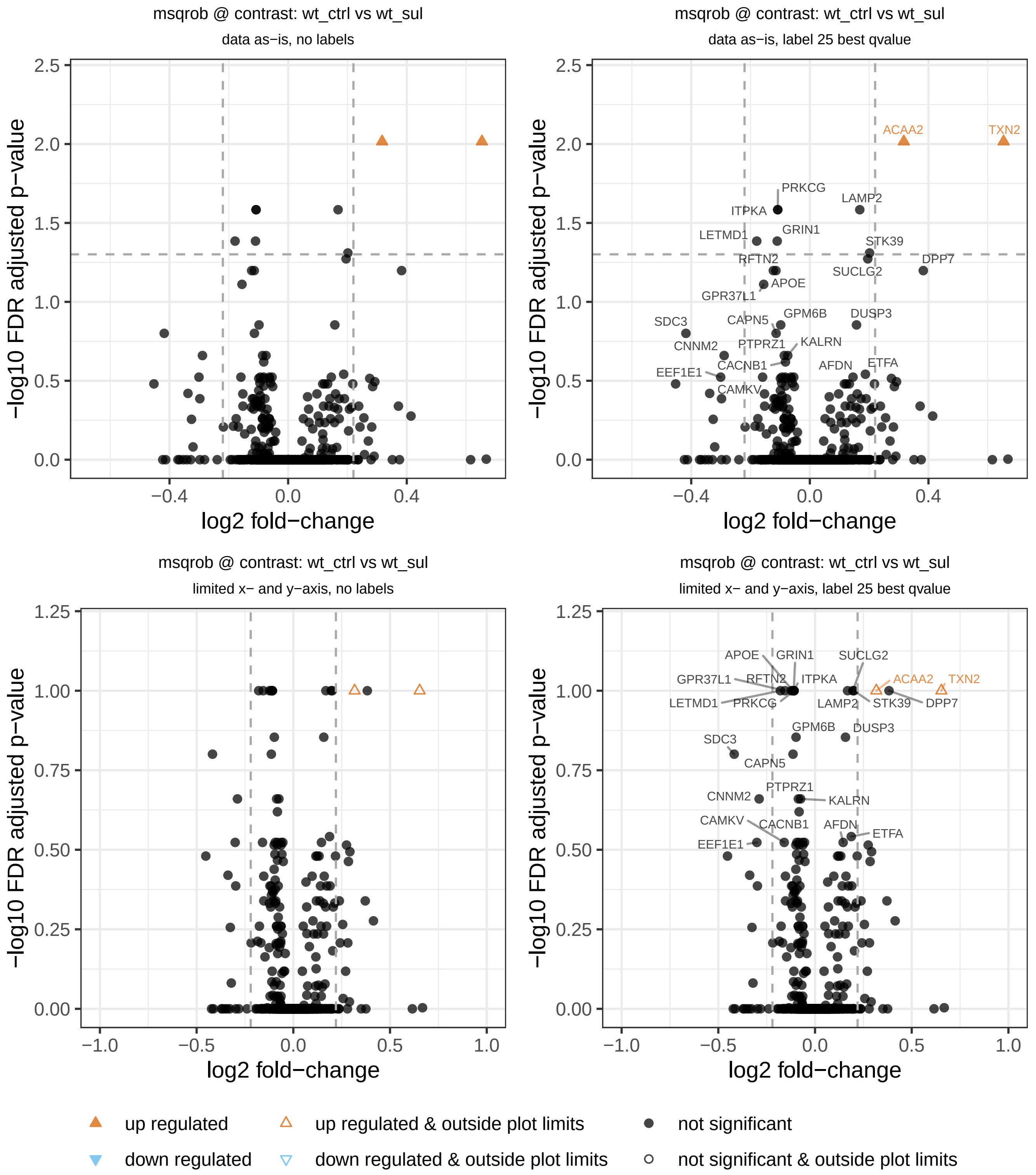
msqrob @ contrast: wt_ctrl vs wt_sul
data as−is, no labels
msqrob @ contrast: wt_ctrl vs wt_sul
data as−is, label 25 best qvalue
msqrob @ contrast: wt_ctrl vs wt_sul
limited x− and y−axis, no labels
msqrob @ contrast: wt_ctrl vs wt_sul
limited x− and y−axis, label 25 best qvalue
−log10 FDR adjusted p−value
log2 fold−change
ACAA2
TXN2
PRKCG
LAMP2
ITPKA
GRIN1
LETMD1
STK39
RFTN2
DPP7
SUCLG2
APOE
GPR37L1
SDC3
CAPN5
GPM6B
DUSP3
CNNM2
PTPRZ1
KALRN
CACNB1
AFDN
ETFA
EEF1E1
CAMKV
up regulated
down regulated
up regulated & outside plot limits
down regulated & outside plot limits
not significant
not significant & outside plot limits

2.1.2 foldchange distribution

Distributions of estimated foldchanges produced by the statistical models. If the mode is far from 0, consider alternative normalization strategies. Do note the scale on the x-axis, for some experiments the foldchanges are very low which in turn may exaggerate this figure.

note; the MSqRob model tends to assign zero (log)foldchange for proteins with minor difference between conditions where the model is very sure the null hypothesis cannot be rejected (shrinkage by the ridge regression model). As a result, many foldchanges will be zero and the density plot for MSqRob may look like a spike instead of the expected Gaussian shape observed in other models

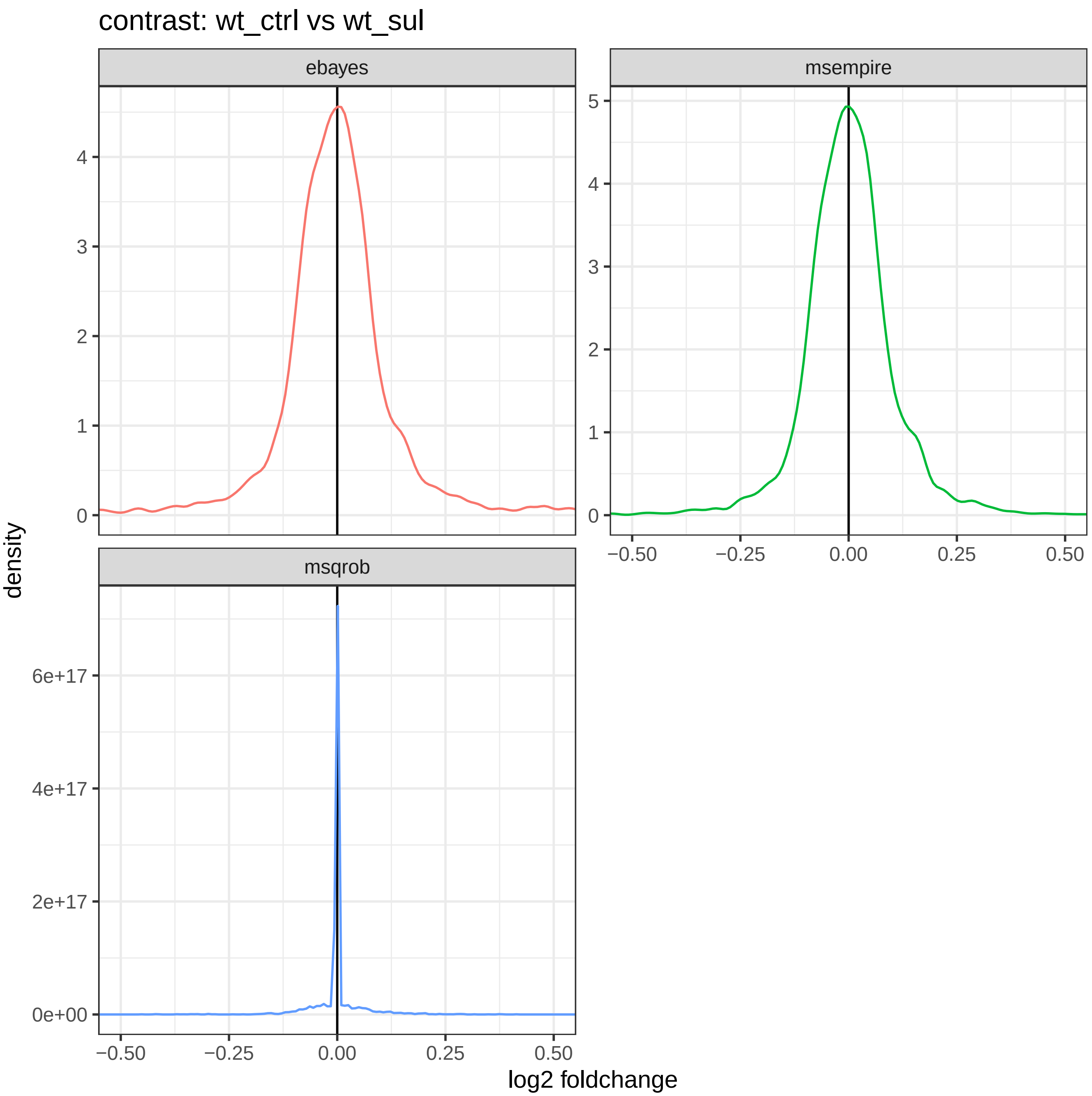

2.1.3 p-value distribution

Histogram of p-values computed by differential expression analysis algorithms, as-is, for quality-control inspection. The horizontal line indicates the expected counts assuming a uniform distribution (total number of p-values divided by number of histogram bins)

See further: https://www.ncbi.nlm.nih.gov/pmc/articles/PMC6164648/

See further: http://varianceexplained.org/statistics/interpreting-pvalue-histogram/

note; the MSqRob and MS-EmpiRe models often yield p-value distributions that show a large peak at p-value 1, these are typically proteins with estimated log foldchanges at/near zero where these models are very sure the null hypothesis cannot be rejected

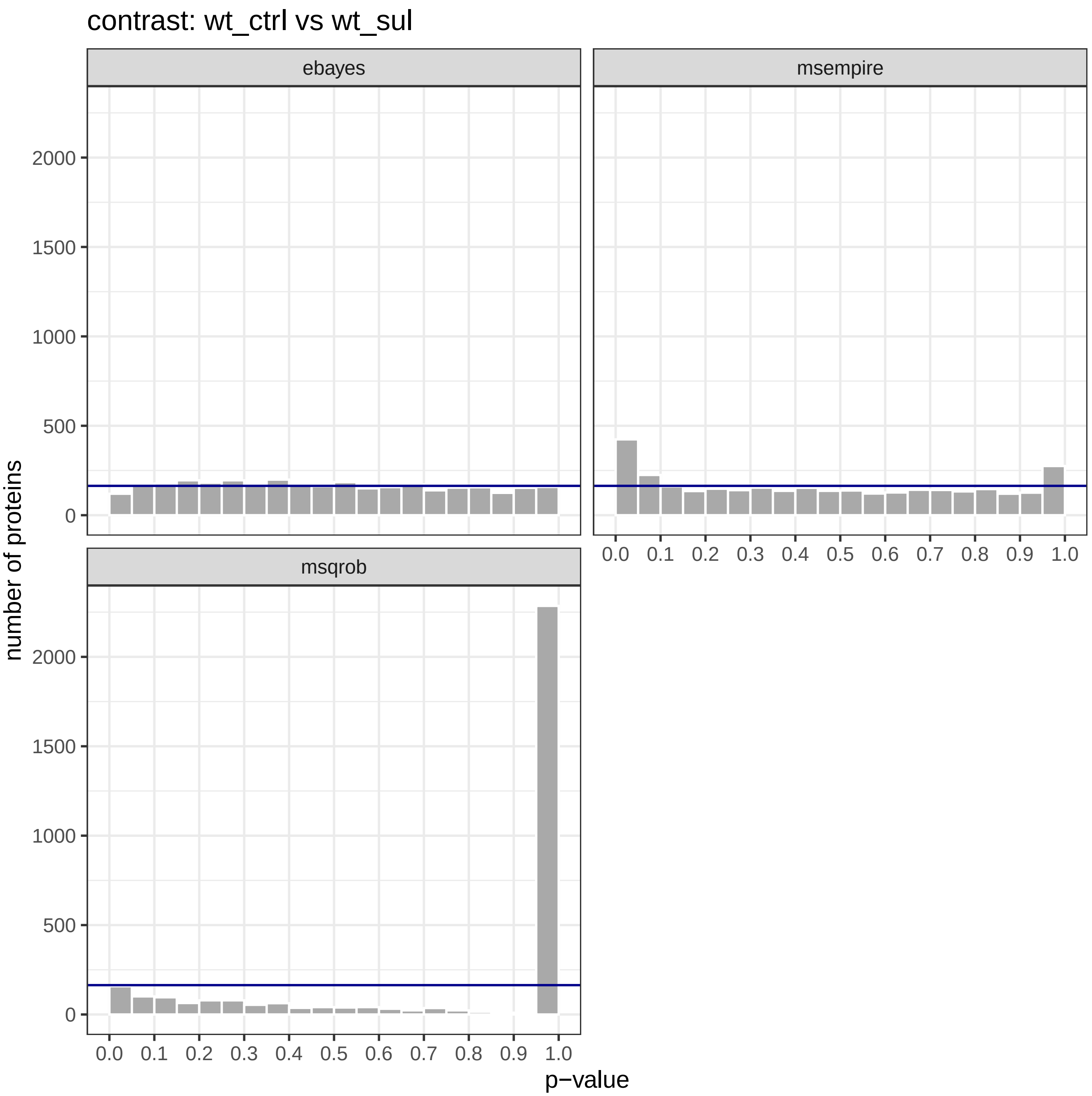

2.2 app_ctrl vs app_sul

- **user setting:** using 'filter by contrast' peptide filtering approach
- 24511 peptides in 3865 proteins remain in the current contrast after peptide filters and are used for the statistical analysis in this section
- qvalue threshold: 0.05
- log2 foldchange threshold: 0.1899503

2.2.1 volcano

The plot title shows the statistical model and contrast (sample groups in the comparison). Left- and right-side figure panels on each row represent the same figure without and with labels for the 25 proteins with lowest p-value.

Bottom figure panels have limited x- and y-axis. For datasets with a small number of strong outliers in p-value or fold-change, which may have a profound effect on the plot scales, this allows inspection of the remainder of the volcano plot without disproportionate influence by 'extreme' values.

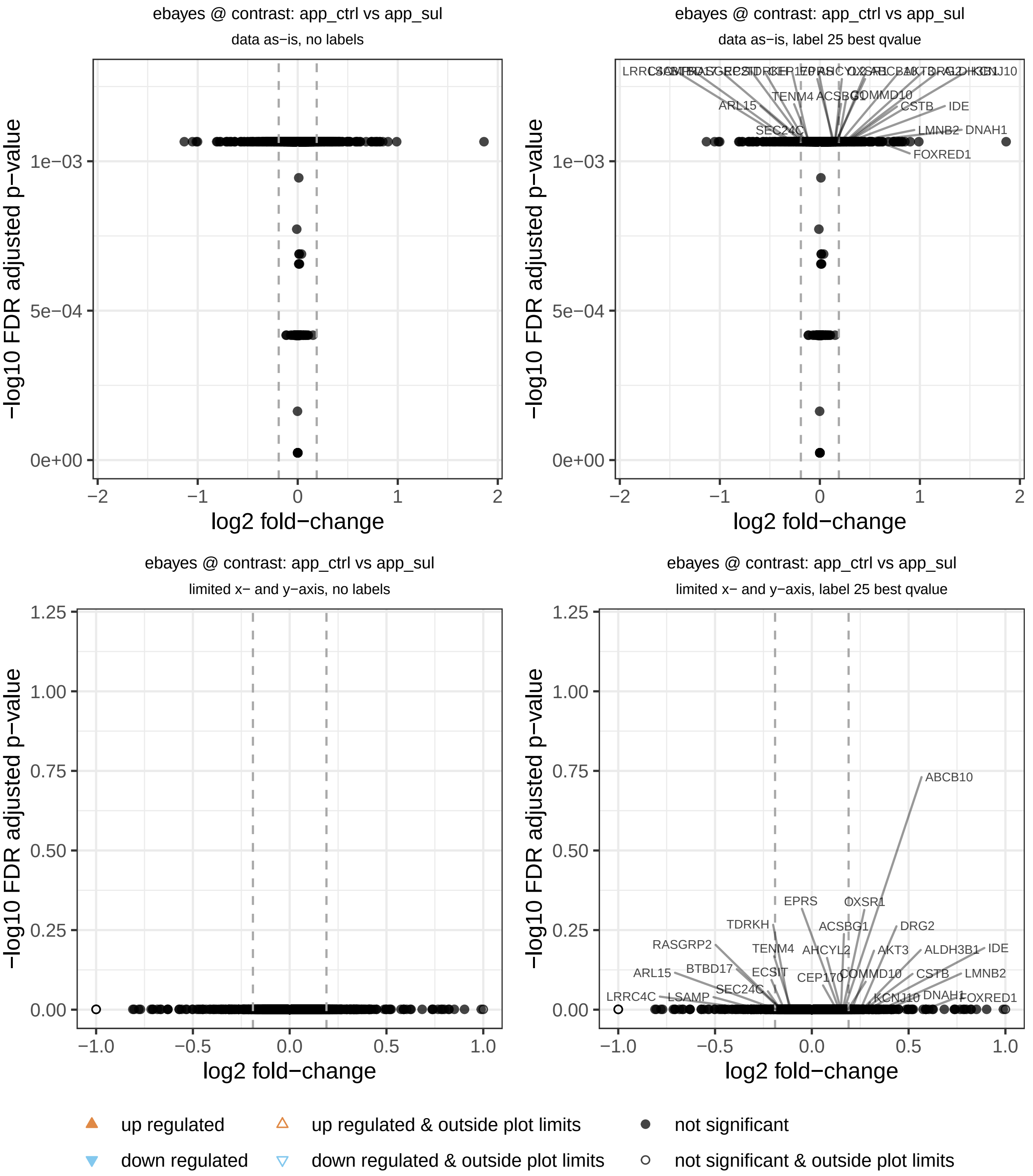

ebayes @ contrast: app_ctrl vs app_sul
data as−is, no labels
−log10 FDR adjusted p−value
log2 fold−change
1e−03
5e−04
0e+00
−2
−1
0
1
2
ebayes @ contrast: app_ctrl vs app_sul
data as−is, label 25 best qvalue
ARL15
TENM4
ACSBG1
COMMD10
CSTB
IDE
SEC24C
LMNB2
DNAH1
FOXRED1
ebayes @ contrast: app_ctrl vs app_sul
limited x− and y−axis, no labels
1.25
1.00
0.75
0.50
0.25
0.00
−1.0
−0.5
0.0
0.5
1.0
ebayes @ contrast: app_ctrl vs app_sul
limited x− and y−axis, label 25 best qvalue
ABCB10
EPRS
OXSR1
TDRKH
ACSBG1
DRG2
RASGRP2
TENM4
AHCYL2
AKT3
ALDH3B1
IDE
ARL15
BTBD17
ECSIT
CEP170
COMMD10
CSTB
LMNB2
SEC24C
LRRC4C
LSAMP
KCNJ10
DNAH1
FOXRED1
up regulated
down regulated
up regulated & outside plot limits
down regulated & outside plot limits
not significant
not significant & outside plot limits

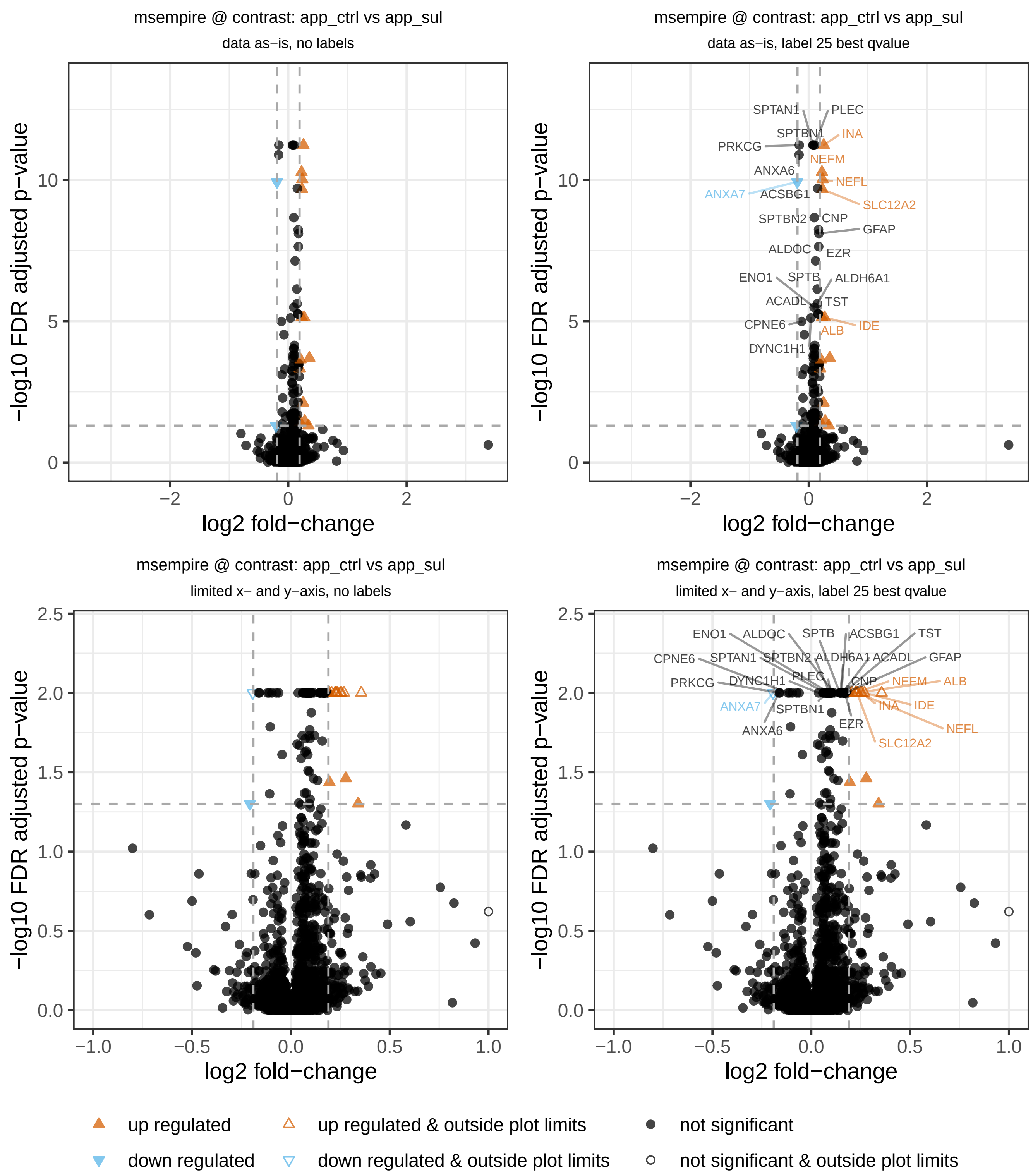
msempire @ contrast: app_ctrl vs app_sul
data as−is, no labels
msempire @ contrast: app_ctrl vs app_sul
data as−is, label 25 best qvalue
SPTAN1
PLEC
SPTBN1
INA
PRKCG
NEFM
ANXA6
NEFL
ANXA7
ACSBG1
SLC12A2
SPTBN2
CNP
GFAP
ALDOC
EZR
ENO1
SPTB
ALDH6A1
ACADL
TST
CPNE6
IDE
ALB
DYNC1H1
msempire @ contrast: app_ctrl vs app_sul
limited x− and y−axis, no labels
msempire @ contrast: app_ctrl vs app_sul
limited x− and y−axis, label 25 best qvalue
ENO1
ALDOC
SPTB
ACSBG1
TST
CPNE6
SPTAN1
SPTBN2
ALDH6A1
ACADL
GFAP
PRKCG
DYNC1H1
PLEC
CNP
NEFM
ALB
ANXA7
INA
IDE
SPTBN1
EZR
NEFL
ANXA6
SLC12A2
−log10 FDR adjusted p−value
log2 fold−change
up regulated
up regulated & outside plot limits
not significant
down regulated
down regulated & outside plot limits
not significant & outside plot limits

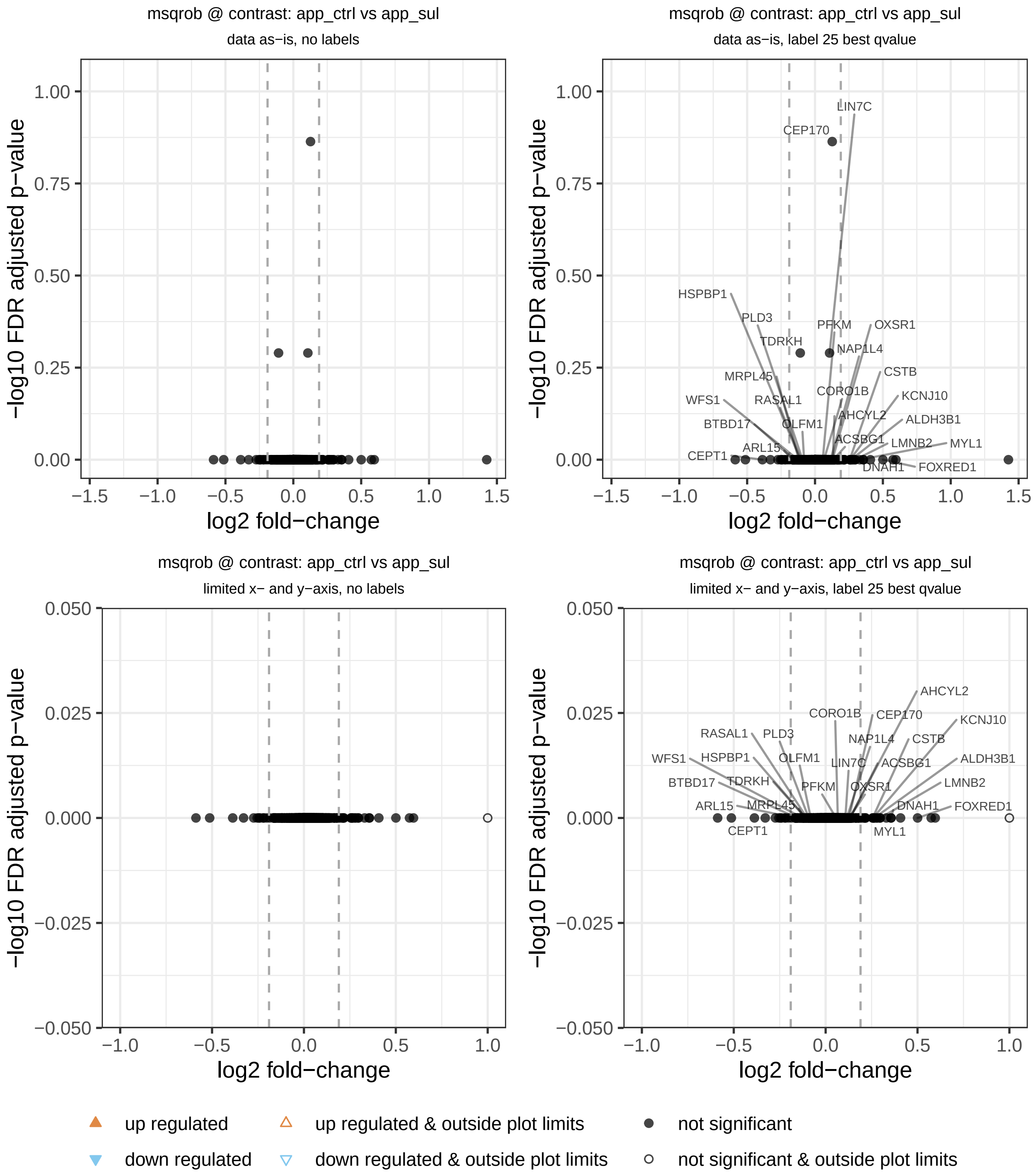
msqrob @ contrast: app_ctrl vs app_sul
data as−is, no labels
data as−is, label 25 best qvalue
limited x− and y−axis, no labels
limited x− and y−axis, label 25 best qvalue
−log10 FDR adjusted p−value
log2 fold−change
LIN7C
CEP170
HSPBP1
PLD3
TDRKH
PFKM
OXSR1
NAP1L4
MRPL45
CSTB
WFS1
RASAL1
CORO1B
KCNJ10
BTBD17
OLFM1
AHCYL2
ALDH3B1
ARL15
ACSBG1
LMNB2
MYL1
CEPT1
DNAH1
FOXRED1
up regulated
up regulated & outside plot limits
not significant
down regulated
down regulated & outside plot limits
not significant & outside plot limits

2.2.2 foldchange distribution

Distributions of estimated foldchanges produced by the statistical models. If the mode is far from 0, consider alternative normalization strategies. Do note the scale on the x-axis, for some experiments the foldchanges are very low which in turn may exaggerate this figure.

note; the MSqRob model tends to assign zero (log)foldchange for proteins with minor difference between conditions where the model is very sure the null hypothesis cannot be rejected (shrinkage by the ridge regression model). As a result, many foldchanges will be zero and the density plot for MSqRob may look like a spike instead of the expected Gaussian shape observed in other models

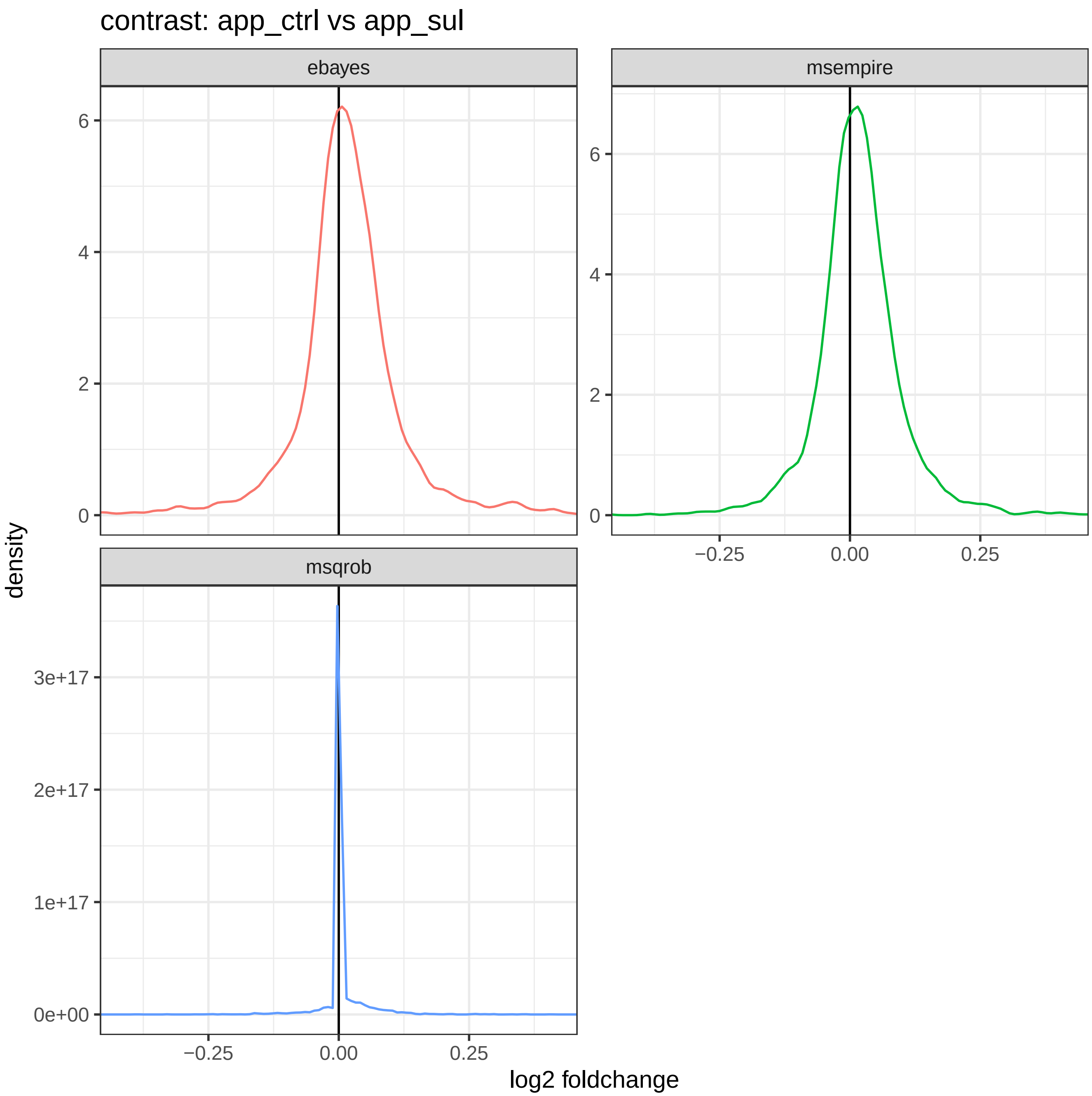

2.2.3 p-value distribution

Histogram of p-values computed by differential expression analysis algorithms, as-is, for quality-control inspection. The horizontal line indicates the expected counts assuming a uniform distribution (total number of p-values divided by number of histogram bins)

See further: https://www.ncbi.nlm.nih.gov/pmc/articles/PMC6164648/

See further: http://varianceexplained.org/statistics/interpreting-pvalue-histogram/

note; the MSqRob and MS-EmpiRe models often yield p-value distributions that show a large peak at p-value 1, these are typically proteins with estimated log foldchanges at/near zero where these models are very sure the null hypothesis cannot be rejected

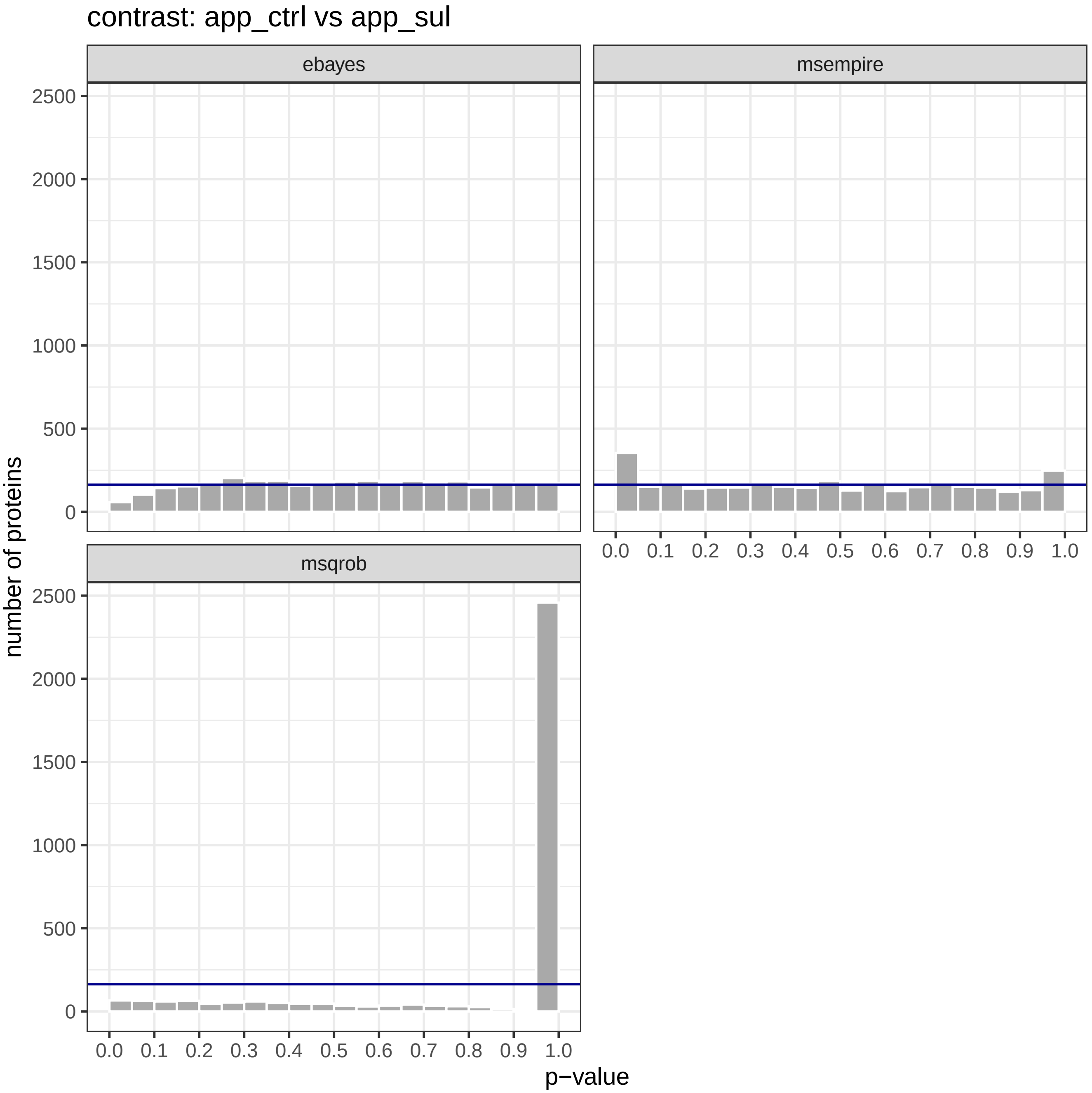

2.3 wt_ctrl vs app_ctrl

- **user setting:** using 'filter by contrast' peptide filtering approach
- 24511 peptides in 3865 proteins remain in the current contrast after peptide filters and are used for the statistical analysis in this section
- qvalue threshold: 0.05
- log2 foldchange threshold: 0.2013298

2.3.1 volcano

The plot title shows the statistical model and contrast (sample groups in the comparison). Left- and right-side figure panels on each row represent the same figure without and with labels for the 25 proteins with lowest p-value.

Bottom figure panels have limited x- and y-axis. For datasets with a small number of strong outliers in p-value or fold-change, which may have a profound effect on the plot scales, this allows inspection of the remainder of the volcano plot without disproportionate influence by 'extreme' values.

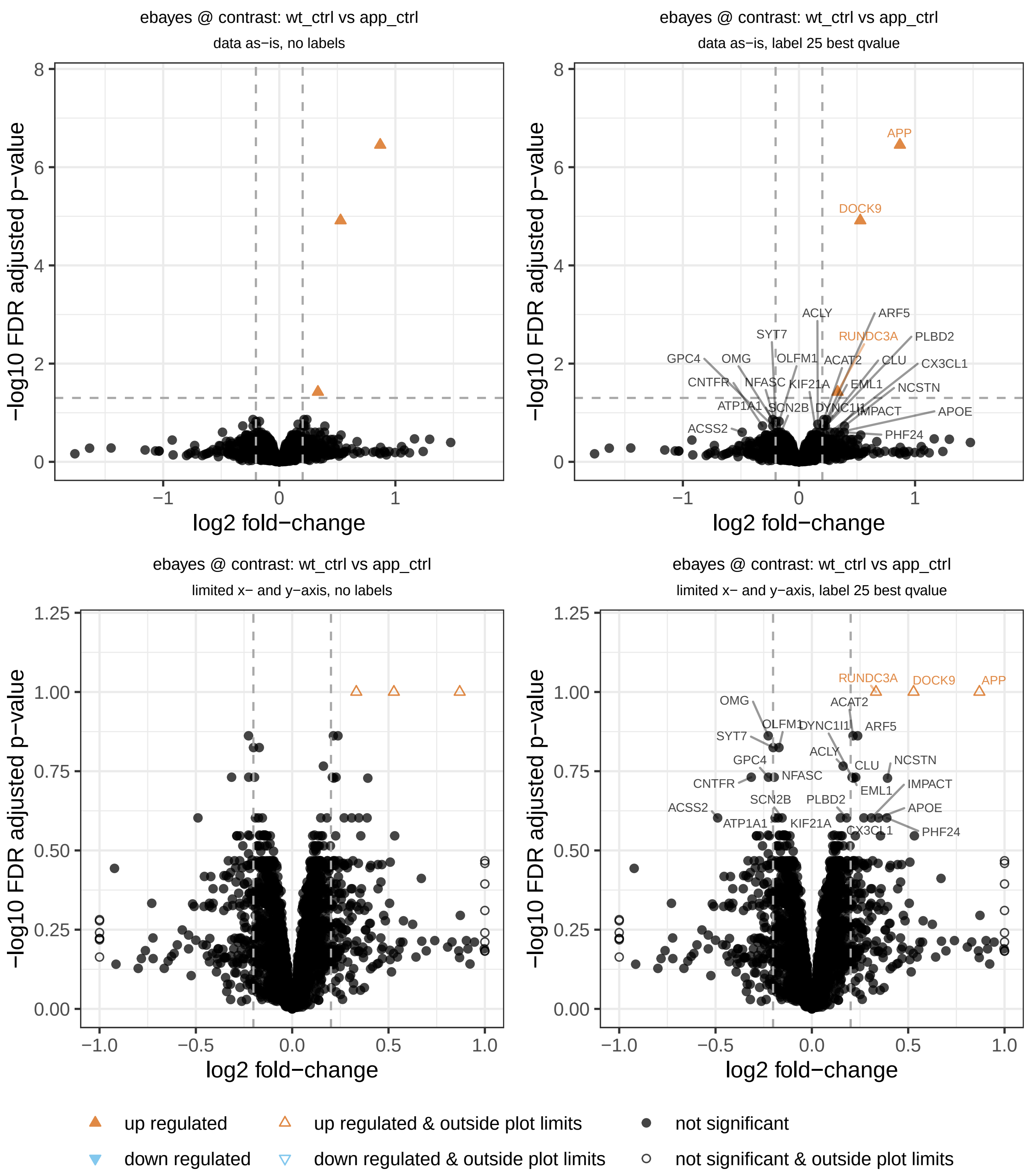
ebayes @ contrast: wt_ctrl vs app_ctrl
data as−is, no labels
−log10 FDR adjusted p−value
log2 fold−change
ebayes @ contrast: wt_ctrl vs app_ctrl
data as−is, label 25 best qvalue
APP
DOCK9
ACLY
ARF5
SYT7
RUNDC3A
PLBD2
GPC4
OMG
OLFM1
ACAT2
CLU
CX3CL1
CNTFR
NFASC
KIF21A
EML1
NCSTN
ATP1A1
SCN2B
DYNC1I1
IMPACT
APOE
ACSS2
PHF24
ebayes @ contrast: wt_ctrl vs app_ctrl
limited x− and y−axis, no labels
ebayes @ contrast: wt_ctrl vs app_ctrl
limited x− and y−axis, label 25 best qvalue
up regulated
up regulated & outside plot limits
not significant
down regulated
down regulated & outside plot limits
not significant & outside plot limits

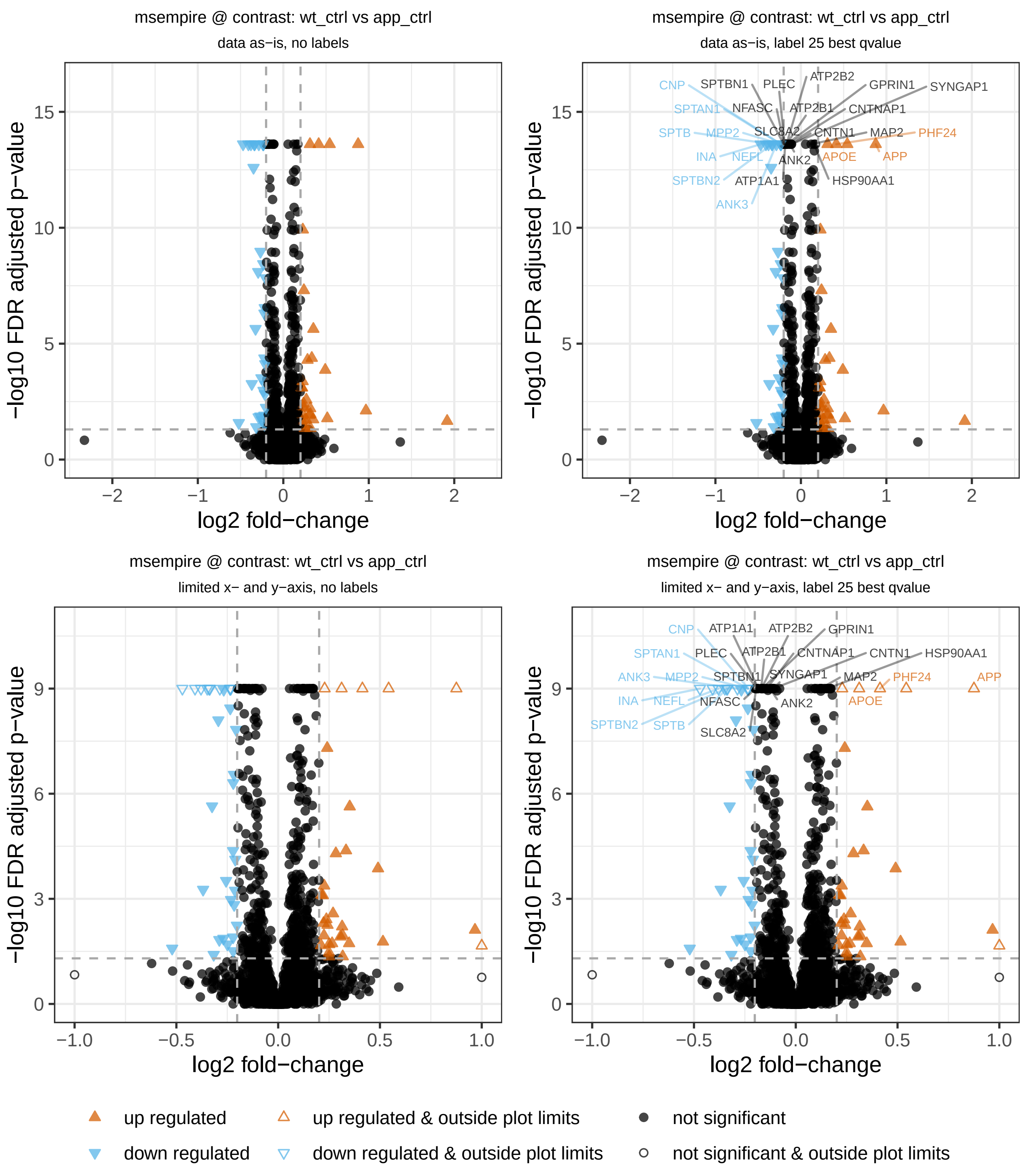
msempire @ contrast: wt_ctrl vs app_ctrl
data as−is, no labels
−log10 FDR adjusted p−value
log2 fold−change
msempire @ contrast: wt_ctrl vs app_ctrl
data as−is, label 25 best qvalue
CNP
SPTBN1
PLEC
ATP2B2
GPRIN1
SYNGAP1
SPTAN1
NFASC
ATP2B1
CNTNAP1
SPTB
MPP2
SLC8A2
CNTN1
MAP2
PHF24
INA
NEFL
ANK2
APOE
APP
SPTBN2
ATP1A1
HSP90AA1
ANK3
msempire @ contrast: wt_ctrl vs app_ctrl
limited x− and y−axis, no labels
msempire @ contrast: wt_ctrl vs app_ctrl
limited x− and y−axis, label 25 best qvalue
up regulated
up regulated & outside plot limits
not significant
down regulated
down regulated & outside plot limits
not significant & outside plot limits

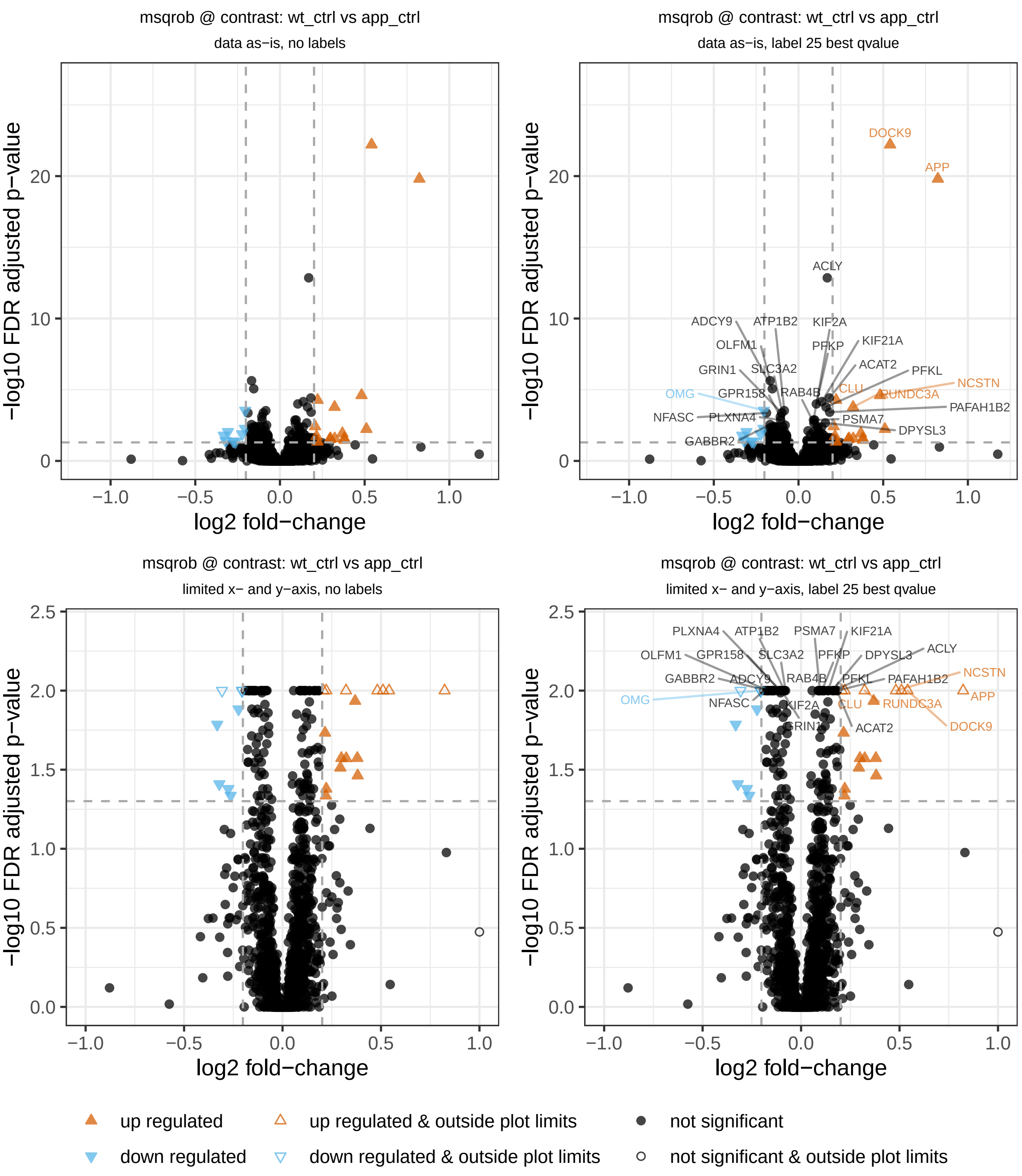
msqrob @ contrast: wt_ctrl vs app_ctrl
data as−is, no labels
−log10 FDR adjusted p−value
log2 fold−change
msqrob @ contrast: wt_ctrl vs app_ctrl
data as−is, label 25 best qvalue
DOCK9
APP
ACLY
ADCY9
ATP1B2
KIF2A
OLFM1
PFKP
KIF21A
ACAT2
PFKL
GRIN1
SLC3A2
NCSTN
OMG
GPR158
RAB4B
CLU
RUNDC3A
PAFAH1B2
NFASC
PLXNA4
PSMA7
DPYSL3
GABBR2
−log10 FDR adjusted p−value
log2 fold−change
msqrob @ contrast: wt_ctrl vs app_ctrl
limited x− and y−axis, no labels
−log10 FDR adjusted p−value
log2 fold−change
msqrob @ contrast: wt_ctrl vs app_ctrl
limited x− and y−axis, label 25 best qvalue
PLXNA4
ATP1B2
PSMA7
KIF21A
ACLY
OLFM1
GPR158
SLC3A2
PFKP
DPYSL3
GABBR2
ADCY9
RAB4B
PFKL
PAFAH1B2
NCSTN
OMG
NFASC
KIF2A
CLU
RUNDC3A
APP
GRIN1
ACAT2
DOCK9
−log10 FDR adjusted p−value
log2 fold−change
up regulated
up regulated & outside plot limits
not significant
down regulated
down regulated & outside plot limits
not significant & outside plot limits

2.3.2 foldchange distribution

Distributions of estimated foldchanges produced by the statistical models. If the mode is far from 0, consider alternative normalization strategies. Do note the scale on the x-axis, for some experiments the foldchanges are very low which in turn may exaggerate this figure.

note; the MSqRob model tends to assign zero (log)foldchange for proteins with minor difference between conditions where the model is very sure the null hypothesis cannot be rejected (shrinkage by the ridge regression model). As a result, many foldchanges will be zero and the density plot for MSqRob may look like a spike instead of the expected Gaussian shape observed in other models

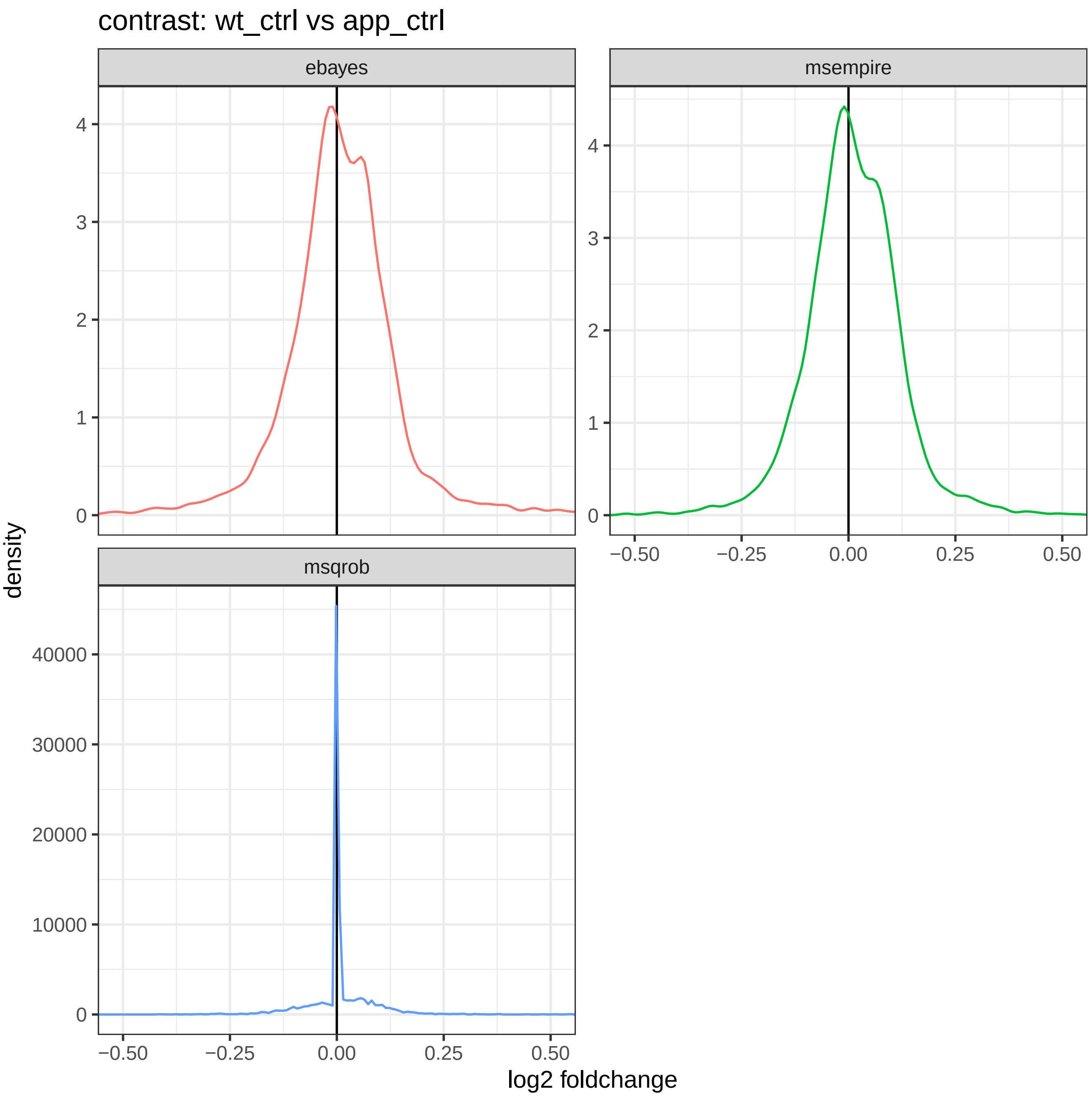

2.3.3 p-value distribution

Histogram of p-values computed by differential expression analysis algorithms, as-is, for quality-control inspection. The horizontal line indicates the expected counts assuming a uniform distribution (total number of p-values divided by number of histogram bins)

See further: https://www.ncbi.nlm.nih.gov/pmc/articles/PMC6164648/

See further: http://varianceexplained.org/statistics/interpreting-pvalue-histogram/

note; the MSqRob and MS-EmpiRe models often yield p-value distributions that show a large peak at p-value 1, these are typically proteins with estimated log foldchanges at/near zero where these models are very sure the null hypothesis cannot be rejected

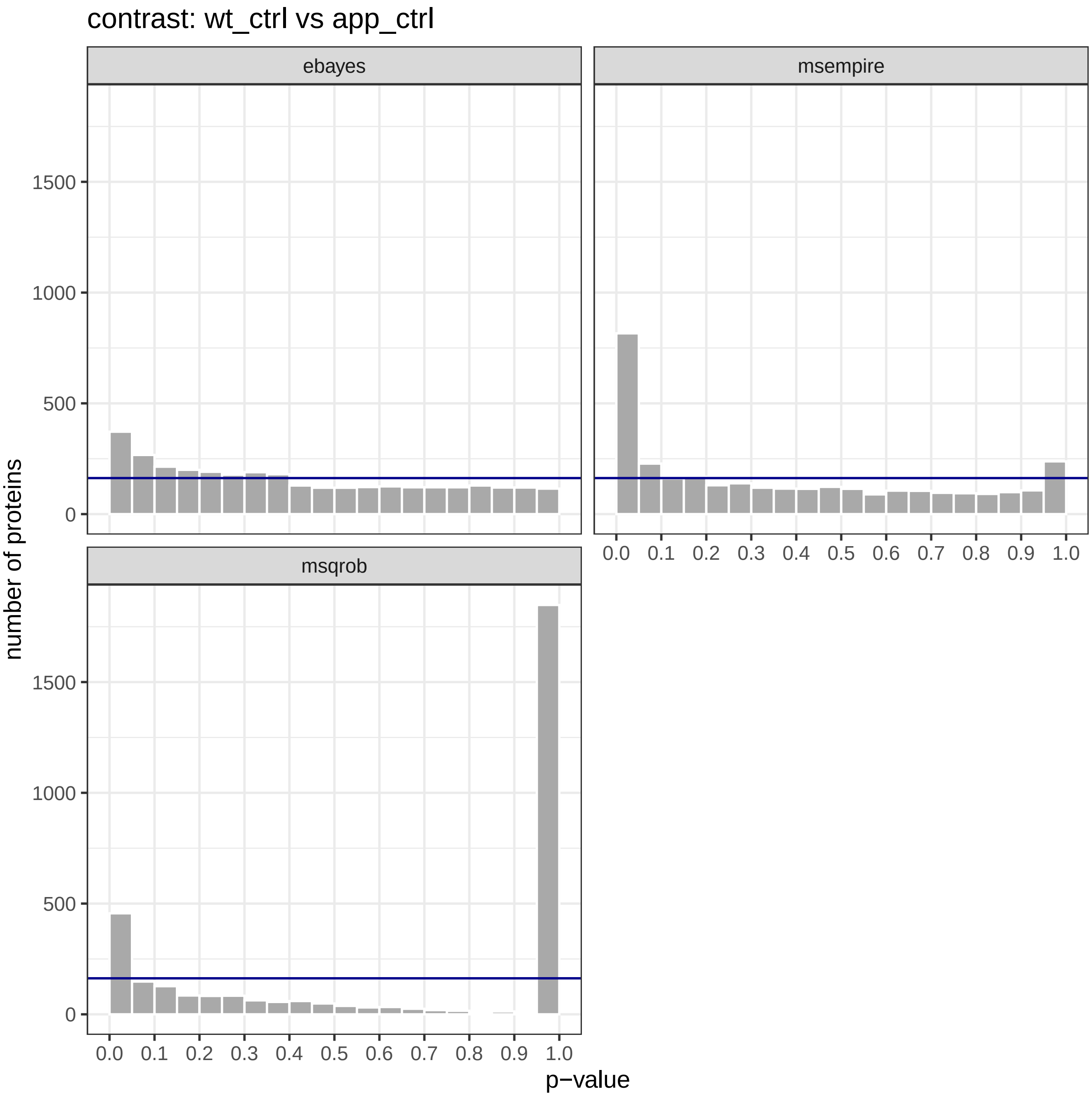

2.4 wt_sul vs app_sul

- **user setting:** using 'filter by contrast' peptide filtering approach
- 24511 peptides in 3865 proteins remain in the current contrast after peptide filters and are used for the statistical analysis in this section
- qvalue threshold: 0.05
- log2 foldchange threshold: 0.2233733

2.4.1 volcano

The plot title shows the statistical model and contrast (sample groups in the comparison). Left- and right-side figure panels on each row represent the same figure without and with labels for the 25 proteins with lowest p-value.

Bottom figure panels have limited x- and y-axis. For datasets with a small number of strong outliers in p-value or fold-change, which may have a profound effect on the plot scales, this allows inspection of the remainder of the volcano plot without disproportionate influence by 'extreme' values.

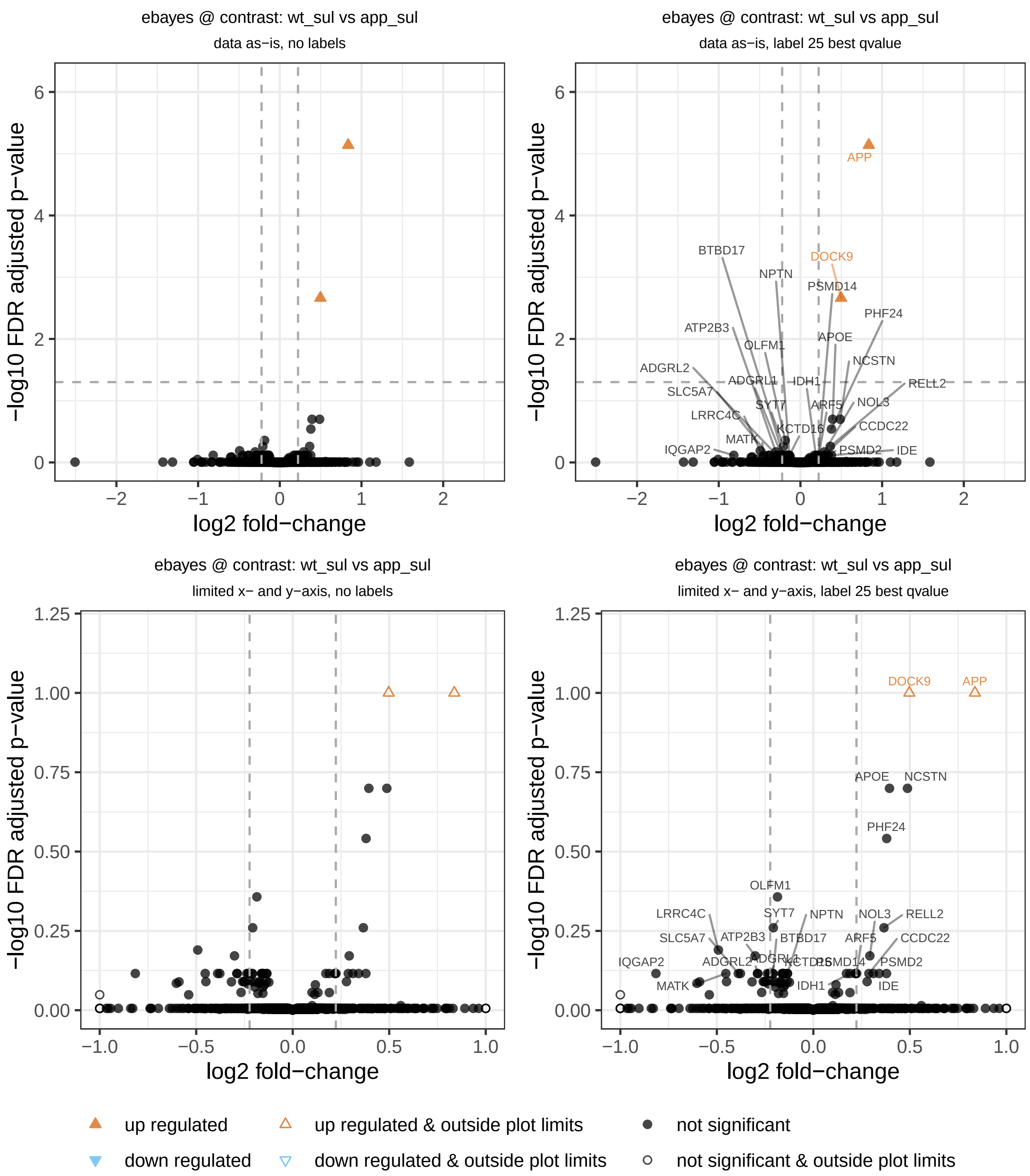
ebayes @ contrast: wt_sul vs app_sul
data as−is, no labels
−log10 FDR adjusted p−value
log2 fold−change
ebayes @ contrast: wt_sul vs app_sul
data as−is, label 25 best qvalue
−log10 FDR adjusted p−value
log2 fold−change
APP
DOCK9
BTBD17
NPTN
PSMD14
PHF24
ATP2B3
APOE
OLFM1
NCSTN
ADGRL2
ADGRL1
IDH1
RELL2
SLC5A7
SYT7
ARF5
NOL3
LRRC4C
CCDC22
KCTD16
MATK
IQGAP2
PSMD2
IDE
ebayes @ contrast: wt_sul vs app_sul
limited x− and y−axis, no labels
−log10 FDR adjusted p−value
log2 fold−change
ebayes @ contrast: wt_sul vs app_sul
limited x− and y−axis, label 25 best qvalue
−log10 FDR adjusted p−value
log2 fold−change
DOCK9
APP
APOE
NCSTN
PHF24
OLFM1
LRRC4C
SYT7
NPTN
NOL3
RELL2
SLC5A7
ATP2B3
BTBD17
ARF5
CCDC22
IQGAP2
ADGRL2
ADGRL1
KCTD16
PSMD14
PSMD2
MATK
IDH1
IDE
up regulated
up regulated & outside plot limits
not significant
down regulated
down regulated & outside plot limits
not significant & outside plot limits

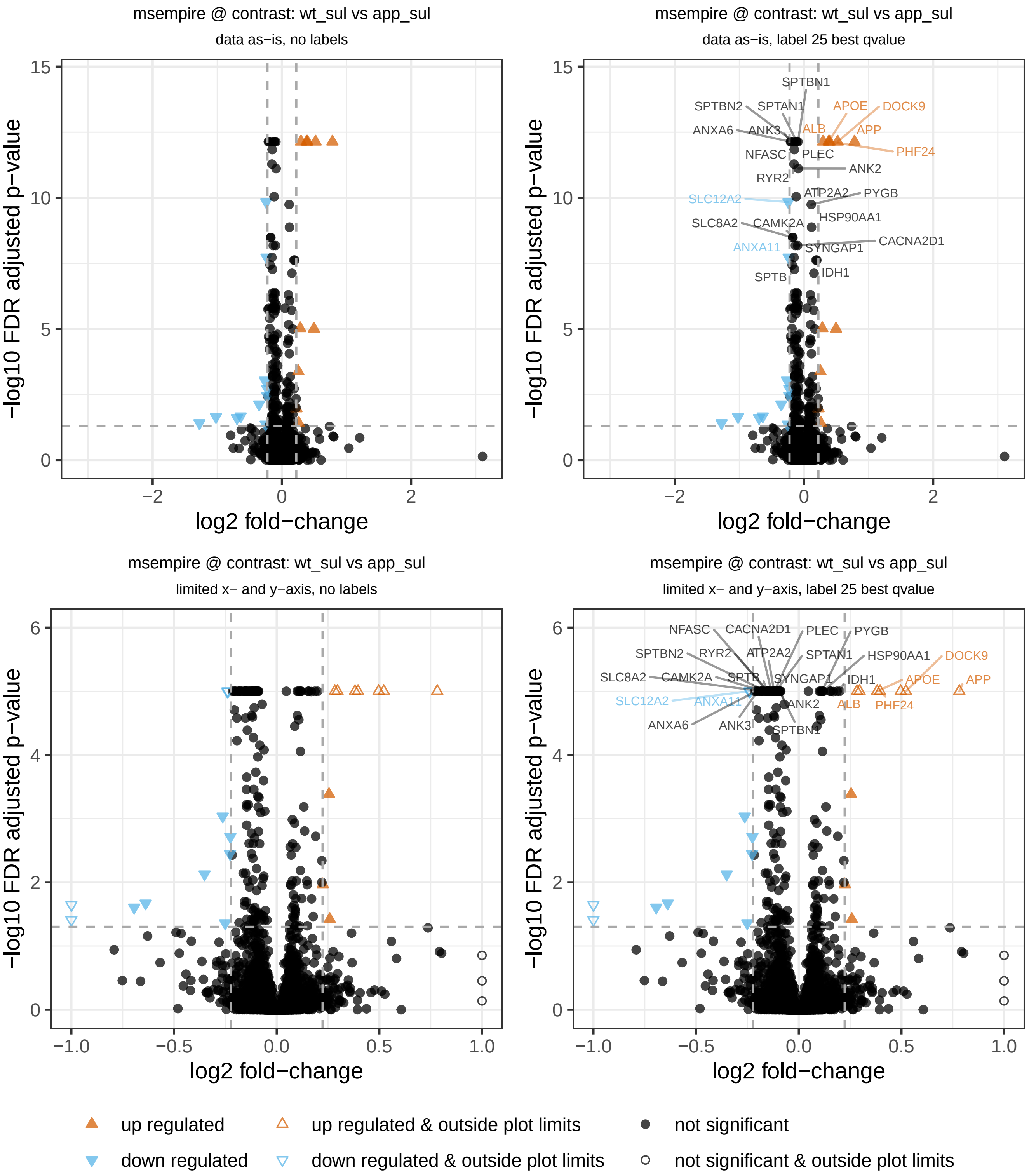
msempire @ contrast: wt_sul vs app_sul
data as−is, no labels
msempire @ contrast: wt_sul vs app_sul
data as−is, label 25 best qvalue
msempire @ contrast: wt_sul vs app_sul
limited x− and y−axis, no labels
msempire @ contrast: wt_sul vs app_sul
limited x− and y−axis, label 25 best qvalue
−log10 FDR adjusted p−value
log2 fold−change
SPTBN1
SPTBN2
SPTAN1
APOE
DOCK9
ANXA6
ANK3
ALB
APP
PHF24
NFASC
PLEC
ANK2
RYR2
ATP2A2
PYGB
SLC12A2
HSP90AA1
SLC8A2
CAMK2A
ANXA11
SYNGAP1
CACNA2D1
SPTB
IDH1
up regulated
down regulated
up regulated & outside plot limits
down regulated & outside plot limits
not significant
not significant & outside plot limits

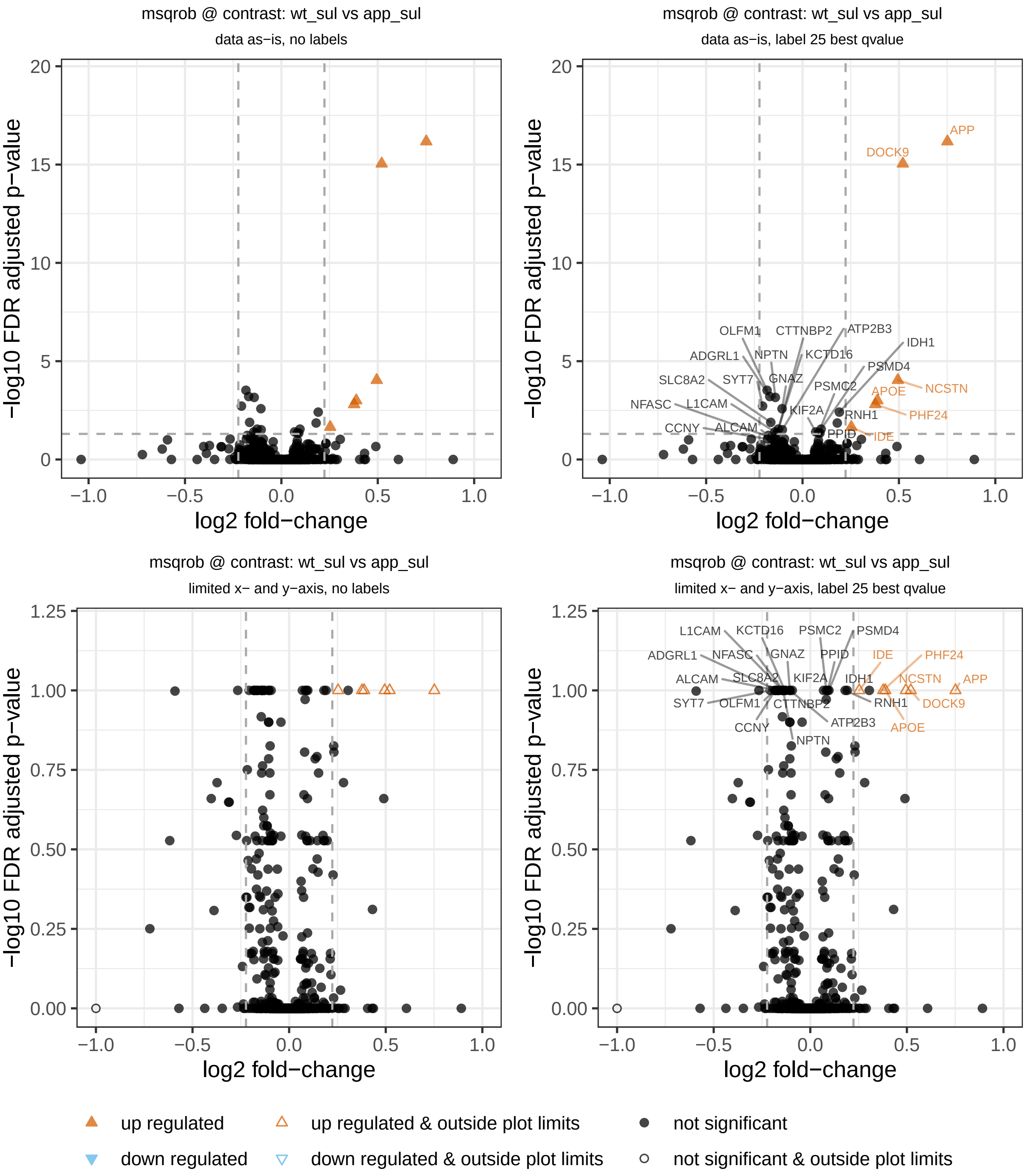
msqrob @ contrast: wt_sul vs app_sul
data as−is, no labels
msqrob @ contrast: wt_sul vs app_sul
data as−is, label 25 best qvalue
msqrob @ contrast: wt_sul vs app_sul
limited x− and y−axis, no labels
msqrob @ contrast: wt_sul vs app_sul
limited x− and y−axis, label 25 best qvalue
−log10 FDR adjusted p−value
log2 fold−change
APP
DOCK9
OLFM1
CTTNBP2
ATP2B3
IDH1
ADGRL1
NPTN
KCTD16
PSMD4
SLC8A2
SYT7
GNAZ
PSMC2
NCSTN
APOE
NFASC
L1CAM
KIF2A
RNH1
PHF24
CCNY
ALCAM
PPID
IDE
up regulated
up regulated & outside plot limits
not significant
down regulated
down regulated & outside plot limits
not significant & outside plot limits

2.4.2 foldchange distribution

Distributions of estimated foldchanges produced by the statistical models. If the mode is far from 0, consider alternative normalization strategies. Do note the scale on the x-axis, for some experiments the foldchanges are very low which in turn may exaggerate this figure.

note; the MSqRob model tends to assign zero (log)foldchange for proteins with minor difference between conditions where the model is very sure the null hypothesis cannot be rejected (shrinkage by the ridge regression model). As a result, many foldchanges will be zero and the density plot for MSqRob may look like a spike instead of the expected Gaussian shape observed in other models

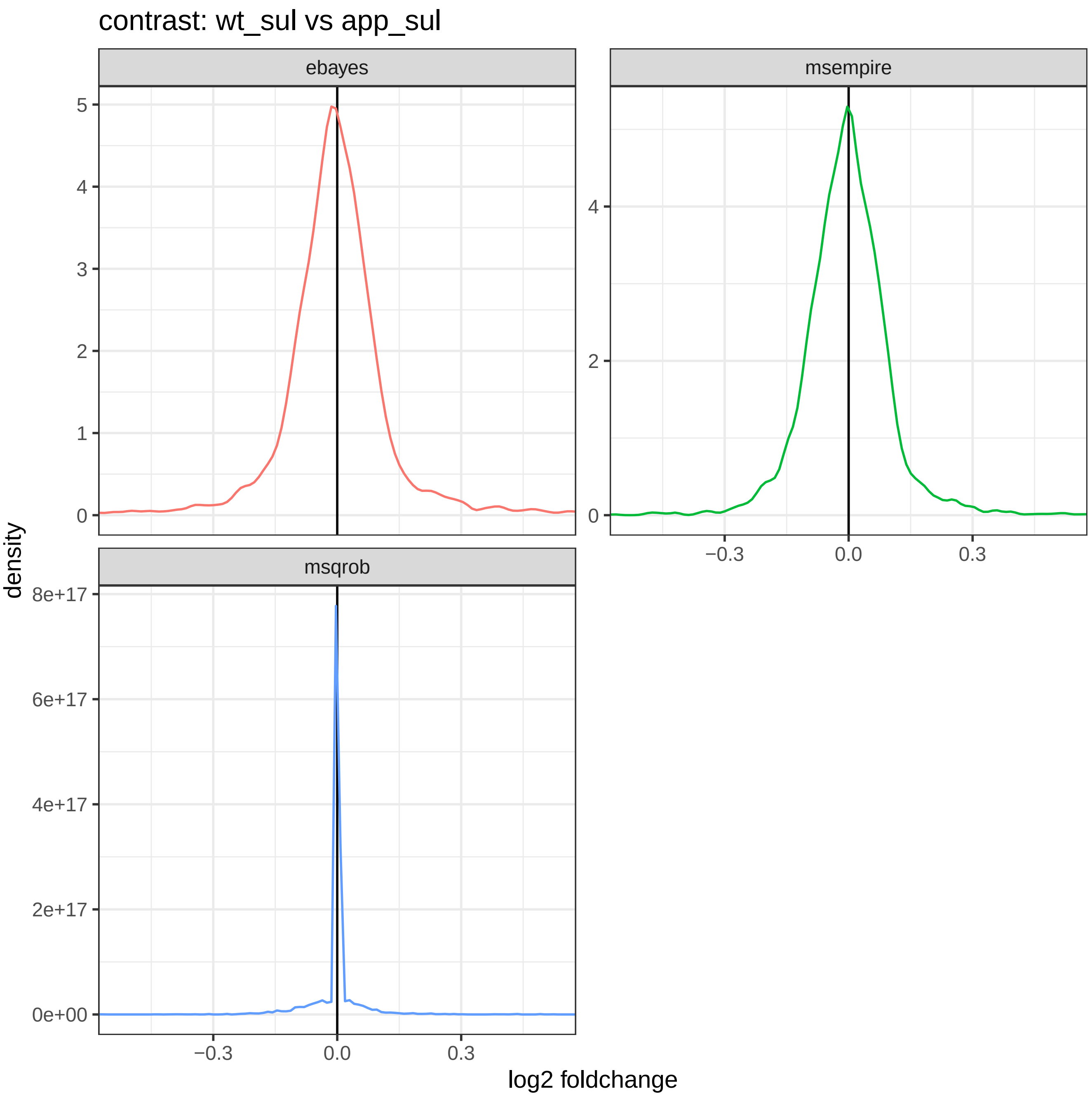

2.4.3 p-value distribution

Histogram of p-values computed by differential expression analysis algorithms, as-is, for quality-control inspection. The horizontal line indicates the expected counts assuming a uniform distribution (total number of p-values divided by number of histogram bins)

See further: https://www.ncbi.nlm.nih.gov/pmc/articles/PMC6164648/

See further: http://varianceexplained.org/statistics/interpreting-pvalue-histogram/

note; the MSqRob and MS-EmpiRe models often yield p-value distributions that show a large peak at p-value 1, these are typically proteins with estimated log foldchanges at/near zero where these models are very sure the null hypothesis cannot be rejected

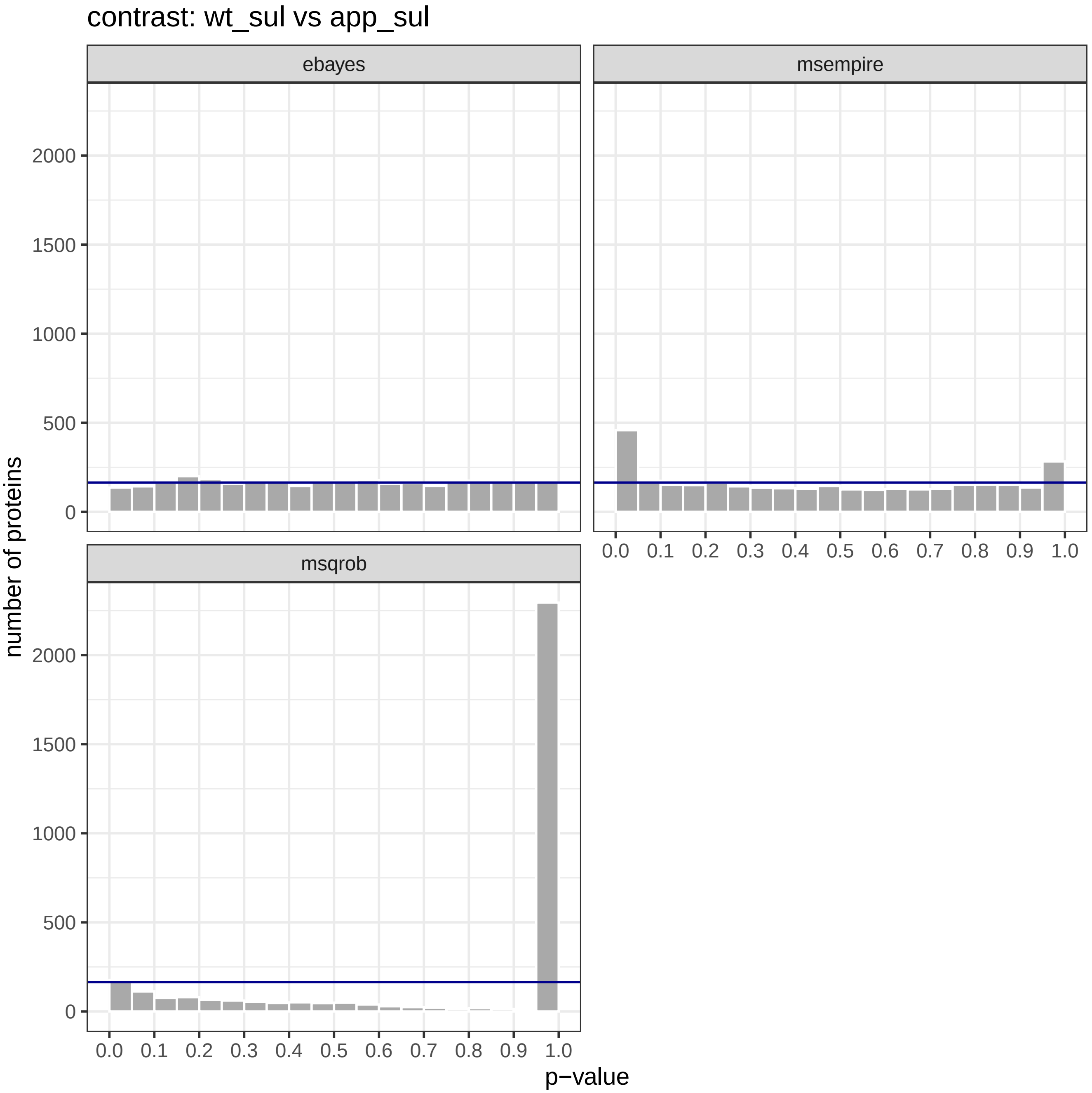

3 Summary of statistical results

Number of proteins found statistically significant in each comparison of sample groups.

contrast	algorithm	#tested	#significant
app__ctrl vs app__sul	ebayes	3267	0
app__ctrl vs app__sul	msempire	3267	15
app__ctrl vs app__sul	msqrob	3267	0
wt__ctrl vs app__ctrl	ebayes	3252	3
wt__ctrl vs app__ctrl	msempire	3252	63
wt__ctrl vs app__ctrl	msqrob	3252	24
wt__ctrl vs wt__sul	ebayes	3281	0
wt__ctrl vs wt__sul	msempire	3281	14
wt__ctrl vs wt__sul	msqrob	3281	2
wt__sul vs app__sul	ebayes	3285	2
wt__sul vs app__sul	msempire	3285	21
wt__sul vs app__sul	msqrob	3285	6

Table 1: Significant hits

4 log

reading Spectronaut report…

24544/26101 precursors remain after selecting the 'best' precursor for each modified sequence

Spectronaut report contains malformed protein IDs (pipe symbol replaced by whitespace), fixing now…

22 / 23293 unique target (plain)sequences were not in the provided spectral library and thus removed

9782/9783 protein accessions and 3865/3866 protein groups were mapped to provided fasta file(s)

filtered proteins: tr|IRT|IRT__KITBiognosysiRTKitFusion

1/3866 proteins matching your filters were removed from the dataset

using 7 threads for multiprocessing

differential abundance analysis for contrast: wt__ctrl vs wt__sul

using data from peptide filter: filter by contrast

log2 foldchange threshold estimated by bootstrap analysis: 0.220

differential abundance analysis for contrast: app__ctrl vs app__sul

using data from peptide filter: filter by contrast

log2 foldchange threshold estimated by bootstrap analysis: 0.190

differential abundance analysis for contrast: wt__ctrl vs app__ctrl

using data from peptide filter: filter by contrast

log2 foldchange threshold estimated by bootstrap analysis: 0.201

differential abundance analysis for contrast: wt__sul vs app__sul

using data from peptide filter: filter by contrast

log2 foldchange threshold estimated by bootstrap analysis: 0.223

5 R command history

This shows the history commands from your R script that starts this pipeline, thereby automatically documenting the parameters/settings used. All lines of executed code since (last) importing data using this R package are shown.

Using this feature

Do not use RStudio's `source` option to execute our pipeline since it will only write `source(...yourscript.R)` to the session history, and consequentially that is all you see in this 'code log'. Instead, select all lines in your script (`control + A`) and then "run" the selected code (either click the run button in RStudio, or use `control + enter`). All lines shown in this section are the same as shown in the RStudio 'History' pane (a tab on the top-right of its UI).

```
dataset = import_dataset_spectronaut(
  filename = "C:/Users/Frank/Downloads/20200525_163724_20200519_christina_Report (1).tsv",
  confidence_threshold = 0.01,
  use_normalized_intensities = FALSE,
  do_plot = TRUE
)
dataset = update_protein_mapping_from_maxquant(dataset,
  path_maxquant = "C:/DATA/remco_speclib_20190301_HC_P2_Full mouse proteome 201804",
  remove_shared = TRUE
)
dataset = import_fasta(dataset,
  files = c(
    "C:/DATA/Remco P2 lib 2018_04 fasta/UP000000589_10090.fasta",
    "C:/DATA/Remco P2 lib 2018_04 fasta/UP000000589_10090_additional.fasta"
  )
)
dataset = remove_proteins_by_name(dataset,
  remove_irt_peptides = TRUE
)
dataset = import_sample_metadata(dataset,
  filename = "C:/Users/Frank/Downloads/samples_input.xlsx"
)
dataset = setup_contrasts(dataset,
  contrast_list = list(
    c(
      "wt_ctrl",
      "wt_sul"
    ), c(
      "app_ctrl",
      "app_sul"
    ), c(
      "wt_ctrl",
      "app_ctrl"
    ),
    c("wt_sul", "app_sul")
  )
)
dataset = analysis_quickstart(dataset,
  filter_min_detect = 4,
  filter_fraction_detect = 0.75,
  filter_by_contrast = TRUE,
  filter_topn_peptides = 0,
  filter_min_peptide_per_prot = 1,
  norm_algorithm = "vwmb",
  dea_algorithm = c(
    "ebayes",
    "msempire", "msqrob"
  ),
  dea_qvalue_threshold = 0.05,
  dea_log2foldchange_threshold = NA,
  output_qc_report = TRUE,
  output_peptide_plots = "none",
  output_abundance_tables = FALSE,
  output_dir = "C:/Users/Frank/Downloads/",
  output_within_timestamped_subdirectory = TRUE
)
```

6 R session info

The computer system and versioning of all R packages used to run this analysis are shown below to facilitate, in combination with the previous section, reproducibility.

Table 2: System

setting	value
version	R version 3.6.1 (2019-07-05)
os	Windows 10 x64
system	x86_64, mingw32
ui	RStudio
language	(EN)
collate	English_United States.1252
ctype	English_United States.1252
tz	Europe/Berlin
date	2020-05-30

Table 3: Attached packages

package	loadedversion	source
dplyr	0.8.4	CRAN (R 3.6.2)
ggplot2	3.2.1	CRAN (R 3.6.1)
msdap	0.1.7.2	NA
rlang	0.4.6	CRAN (R 3.6.3)
testthat	2.3.2	CRAN (R 3.6.3)
tibble	2.1.3	CRAN (R 3.6.1)
tidyr	1.0.2	CRAN (R 3.6.2)

Table 4: Packages that are not attached

package	loadedversion	source
affy	1.64.0	Bioconductor
affyio	1.56.0	Bioconductor
askpass	1.1	CRAN (R 3.6.1)
assertthat	0.2.1	CRAN (R 3.6.1)
backports	1.1.5	CRAN (R 3.6.1)
Biobase	2.46.0	Bioconductor
BiocGenerics	0.32.0	Bioconductor
BiocManager	1.30.10	CRAN (R 3.6.3)
BiocParallel	1.20.1	Bioconductor
bit	1.1-14	CRAN (R 3.6.0)
bit64	0.9-7	CRAN (R 3.6.0)
boot	1.3-24	CRAN (R 3.6.2)
callr	3.4.3	CRAN (R 3.6.3)
cli	2.0.2	CRAN (R 3.6.3)
codetools	0.2-16	CRAN (R 3.6.1)
colorspace	1.4-1	CRAN (R 3.6.1)
cowplot	1.0.0	CRAN (R 3.6.1)
crayon	1.3.4	CRAN (R 3.6.1)

package	loadedversion	source
data.table	1.12.8	CRAN (R 3.6.2)
desc	1.2.0	CRAN (R 3.6.1)
devtools	2.3.0	CRAN (R 3.6.3)
digest	0.6.25	CRAN (R 3.6.1)
doParallel	1.0.15	CRAN (R 3.6.1)
ellipsis	0.3.0	CRAN (R 3.6.1)
evaluate	0.14	CRAN (R 3.6.1)
fansi	0.4.1	CRAN (R 3.6.2)
farver	2.0.3	CRAN (R 3.6.2)
forcats	0.4.0	CRAN (R 3.6.1)
foreach	1.4.8	CRAN (R 3.6.2)
formatR	1.7	CRAN (R 3.6.1)
fs	1.3.1	CRAN (R 3.6.1)
ggpubr	0.2.5	CRAN (R 3.6.2)
ggrepel	0.8.1	CRAN (R 3.6.1)
ggsignif	0.6.0	CRAN (R 3.6.1)
glue	1.4.1	CRAN (R 3.6.3)
gridExtra	2.3	CRAN (R 3.6.1)
gtable	0.3.0	CRAN (R 3.6.1)
gtools	3.8.1	CRAN (R 3.6.0)
hms	0.5.3	CRAN (R 3.6.2)
htmltools	0.4.0	CRAN (R 3.6.1)
impute	1.60.0	Bioconductor
IRanges	2.20.2	Bioconductor
iterators	1.0.12	CRAN (R 3.6.1)
knitr	1.28	CRAN (R 3.6.2)
labeling	0.3	CRAN (R 3.6.0)
lattice	0.20-40	CRAN (R 3.6.1)
lazyeval	0.2.2	CRAN (R 3.6.1)
lifecycle	0.1.0	CRAN (R 3.6.1)
limma	3.42.2	Bioconductor
lme4	1.1-21	CRAN (R 3.6.1)
magrittr	1.5	CRAN (R 3.6.1)
MALDIquant	1.19.3	CRAN (R 3.6.1)
MASS	7.3-51.5	CRAN (R 3.6.2)
Matrix	1.2-18	CRAN (R 3.6.2)
matrixStats	0.55.0	CRAN (R 3.6.1)
memoise	1.1.0	CRAN (R 3.6.1)
minqa	1.2.4	CRAN (R 3.6.1)
msEmpiRe	0.1.0	Github (zimmerlab/MS-EmpiRe@ae985a9)
MSnbase	2.12.0	Bioconductor
munsell	0.5.0	CRAN (R 3.6.1)
mzID	1.24.0	Bioconductor
mzR	2.20.0	Bioconductor
ncdf4	1.17	CRAN (R 3.6.1)
nlme	3.1-144	CRAN (R 3.6.2)
nloptr	1.2.1	CRAN (R 3.6.1)
openxlsx	4.1.4	CRAN (R 3.6.2)
packrat	0.5.0	CRAN (R 3.6.3)
patchwork	1.0.0	CRAN (R 3.6.3)
pcaMethods	1.78.0	Bioconductor

package	loadedversion	source
pdftools	2.3	CRAN (R 3.6.1)
pillar	1.4.3	CRAN (R 3.6.2)
pkgbuild	1.0.6	CRAN (R 3.6.1)
pkgconfig	2.0.3	CRAN (R 3.6.1)
pkgload	1.0.2	CRAN (R 3.6.1)
plyr	1.8.5	CRAN (R 3.6.2)
preprocessCore	1.48.0	Bioconductor
prettyunits	1.1.1	CRAN (R 3.6.2)
pROC	1.16.1	CRAN (R 3.6.2)
processx	3.4.2	CRAN (R 3.6.2)
ProtGenerics	1.18.0	Bioconductor
ps	1.3.2	CRAN (R 3.6.2)
purrr	0.3.3	CRAN (R 3.6.1)
qpdf	1.1	CRAN (R 3.6.1)
R6	2.4.1	CRAN (R 3.6.1)
RColorBrewer	1.1-2	CRAN (R 3.6.0)
Rcpp	1.0.3	CRAN (R 3.6.1)
readr	1.3.1	CRAN (R 3.6.1)
remotes	2.1.1	CRAN (R 3.6.2)
reshape2	1.4.3	CRAN (R 3.6.1)
rmarkdown	2.1	CRAN (R 3.6.2)
rprojroot	1.3-2	CRAN (R 3.6.1)
rstudioapi	0.11	CRAN (R 3.6.2)
S4Vectors	0.24.3	Bioconductor
scales	1.1.0	CRAN (R 3.6.1)
sessioninfo	1.1.1	CRAN (R 3.6.1)
stringi	1.4.6	CRAN (R 3.6.2)
stringr	1.4.0	CRAN (R 3.6.1)
styler	1.3.2	CRAN (R 3.6.2)
tidyselect	1.0.0	CRAN (R 3.6.2)
tinytex	0.20	CRAN (R 3.6.2)
usethis	1.6.1	CRAN (R 3.6.3)
utf8	1.1.4	CRAN (R 3.6.1)
vctrs	0.2.3	CRAN (R 3.6.2)
viridis	0.5.1	CRAN (R 3.6.1)
viridisLite	0.3.0	CRAN (R 3.6.1)
vsn	3.54.0	Bioconductor
withr	2.1.2	CRAN (R 3.6.1)
xfun	0.12	CRAN (R 3.6.2)
XML	3.98-1.20	CRAN (R 3.6.0)
xtable	1.8-4	CRAN (R 3.6.1)
yaml	2.2.1	CRAN (R 3.6.2)
zip	2.0.4	CRAN (R 3.6.1)
zlibbioc	1.32.0	Bioconductor
